# Hygiene requirements for cleaning and disinfection of surfaces: recommendation of the Commission for Hospital Hygiene and Infection Prevention (KRINKO) at the Robert Koch Institute

**DOI:** 10.3205/dgkh000468

**Published:** 2024-03-05

**Authors:** Commission for Hospital Hygiene and Infection Prevention (KRINKO)

**Affiliations:** 1 Robert Koch Institute, Berlin, Germany

**Keywords:** disinfecting surface cleaning, surface disinfection, infection prevention and control, health care facilities, recommendation, Commission for Hospital Hygiene and Infection Prevention at the Robert Koch Institute, KRINKO

## Abstract

This recommendation of the Commission for Hospital Hygiene and Infection Prevention (KRINKO) addresses not only hospitals, but also outpatient health care facilities and compiles current evidence.

The following criteria are the basis for the indications for cleaning and disinfection: Infectious bioburden and tenacity of potential pathogens on surfaces and their transmission routes, influence of disinfecting surface cleaning on the rate of nosocomial infections, interruption of cross infections due to multidrug-resistant organisms, and outbreak control by disinfecting cleaning within bundles. The criteria for the selection of disinfectants are determined by the requirements for effectiveness, the efficacy spectrum, the compatibility for humans and the environment, as well as the risk potential for the development of tolerance and resistance. Detailed instructions on the organization and implementation of cleaning and disinfection measures, including structural and equipment requirements, serve as the basis for their implementation. Since the agents for surface disinfection and disinfecting surface cleaning have been classified as biocides in Europe since 2013, the regulatory consequences are explained. As possible addition to surface disinfection, probiotic cleaning, is pointed out. In an informative appendix (only in German), the pathogen characteristics for their acquisition of surfaces, such as tenacity, infectious dose and biofilm formation, and the toxicological and ecotoxicological characteristics of microbicidal agents as the basis for their selection are explained, and methods for the evaluation of the resulting quality of cleaning or disinfecting surface cleaning are presented.

## Abstract (de)

In Deutschland werden die Anforderungen an die Hygiene im Gesundheitswesen in Form von Empfehlungen von der Kommission für Krankenhaushygiene und Infektionsprävention (KRINKO) veröffentlicht. Die KRINKO und ihre ehrenamtliche Arbeit legitimieren sich aus dem Auftrag nach § 23 des Infektionsschutzgesetzes (IfSG).

Die deutsche Originalfassung dieses Dokuments wurde im Oktober 2022 veröffentlicht und wird jetzt auf Englisch der internationalen Fachöffentlichkeit zur Verfügung gestellt.

Das Dokument enthält Empfehlungen für die Reinigung und Desinfektion von Oberflächen in Krankenhäusern und ambulanten medizinischen Einrichtungen. Anhand folgender Kriterien werden die Indikationen zur Reinigung und Desinfektion begründet: Infektiöser Bioburden und Tenazität potentieller Pathogene auf Oberflächen sowie deren Übertragungsmöglichkeiten, Einfluss der desinfizierenden Flächenreinigung auf die Rate nosokomialer Infektionen, Unterbrechung von Kreuzinfektionen durch multiresistente Erreger sowie Ausbruchkontrolle durch desinfizierende Flächenreinigung als Bestandteil von Interventionsbündeln. Als Kriterien zur Auswahl der Desinfektionsmittel werden die Wirkungsanforderungen, das Wirkspektrum, die Verträglichkeit für Mensch und Umwelt sowie das Risikopotential für die Entwicklung von Toleranz und Resistenz dargestellt. Detaillierte Hinweise zur Organisation und Durchführung der Reinigung und Desinfektion einschließlich baulicher und apparativer Voraussetzungen dienen als Grundlage für die Umsetzung der Maßnahmen. Da die Mittel zur Flächendesinfektion und desinfizierenden Flächenreinigung seit 2013 in Europa als Biozid eingestuft sind, werden die sich daraus ergebenden regulativen Konsequenzen erläutert. Als mögliche Ergänzung zur Flächendesinfektion wird auf die probiotische Reinigung hingewiesen. In einem informativen Anhang (nicht ins Englische übersetzt; als *Electronic Supplementary Material* in der Onlineversion der deutschen Originalfassung dieser Empfehlung) werden die für die Akquirierung von Flächen relevanten Erregermerkmale Tenazität, infektiöse Dosis und Biofilmbildung, die zur Auswahl der Mittel dienenden toxikologische und ökotoxikologische Merkmale mikrobizider Wirkstoffe sowie Methoden zur Bewertung der Ergebnisqualität der Reinigung bzw. desinfizierenden Flächenreinigung erläutert.

## Table of contents

1 Introduction

1.1 Objectives

1.2 Scope of application

1.3 Relation to other KRINKO recommendations, Medical Device law, Biocidal Products Regulation and the list of disinfectants

2 Risk assessment of surfaces and prevention potential of surface cleaning, disinfecting surface cleaning and surface disinfection

2.1 Risk differentiation of near-patient (high-touch) and patient-remote (low-touch) surfaces

2.2 Occurrence and tenacity of nosocomial pathogens in the patient environment and reduction of the pathogen load through disinfection measures

2.3 Infection epidemiological studies

2.4 Conclusions

3 Surface cleaning, disinfecting surface cleaning and surface disinfection

3.1 Definitions

3.2 Effectiveness of surface cleaning and disinfecting surface cleaning

4 Cleaning and disinfection measures in different risk areas

4.1 Assignment of rooms to risk areas

4.2 Information on risks when applying surface disinfectants

5 Requirements for surface cleaning and disinfection procedures and selection criteria

5.1 Efficacy and activity spectrum

5.2 Contact time

5.3 Preventing the spread of pathogens

5.4 Prevention of selection and development of resistance

5.5 Risks to humans and the environment

5.6 Waste disposal

5.7 Staff protection

5.8 Fire protection

5.9 Conclusions

6 Procedures for surface cleaning, disinfecting surface cleaning and surface disinfection

6.1 Methods using chemical disinfectants

6.2 Non-contact equipment-based procedures

6.3 Probiotic cleaning methods

7 Building and equipment requirements

7.1 Rooms and furnishings

7.2 Requirements for surfaces in medical facilities with regard to cleaning and disinfection

7.3 Equipment requirements

7.4 Material compatibility with cleaning and disinfection procedures

7.5 Anti-adhesive and antimicrobially effective surfaces

8 Quality assurance

8.1 Requirements for staff, human and material resources

8.2 Hygiene plan

8.3 Implementation of monitoring

9 Recommendations

Abbreviations

References

### Categories within the recommendations of the Commission for Hospital Hygiene and Infection Prevention (KRINKO)

The recommendations given in the following document are based on the current categories of the recommendations of the Commission for Hospital Hygiene and Infection Prevention (KRINKO) from 2010 [1] . These are listed in Table 1 (Tab. 1). to provide information to the interested, non-German

#### Legal notice

This translation is intended solely to provide information to the interested, non-German-reading public. Any discrepancies or differences that may arise in translation of the official German version of the recommendation of the Commission for Hospital Hygiene and Infection Prevention (KRINKO) “Anforderungen an die Hygiene bei der Reinigung und Desinfektion von Flächen” (Bundesgesundheitsbl 2022; 65:1074–1115, https://doi.org/10.1007/ s00103-022-03576-1) are not binding and have no legal effect. 

The erratum (28/9/2023) of Tab. 4 the official German version (Bundesgesundheitsbl 2023; 66:1302–1303, https://doi.org/10.1007/s00103-023-03770-9) has already been considered in this translation.

#### Legal notice in German

**Rechtlicher Hinweis**

Rechtlich bindend ist die deutsche Originalfassung dieser Empfehlung der Kommission für Krankenhaushygiene und Infektionsprävention (KRINKO) „Anforderungen an die Hygiene bei der Reinigung und Desinfektion von Flächen“ (Bundesgesundheitsbl 2022; 65:1074–1115, https://doi.org/10.1007/s00103-022-03576-1). Die englische Fassung dient der Information der internationalen Fachöffentlichkeit. Das Erratum vom 28.09.2023 (Bundesgesundheitsbl 2023; 66:1302–1303, https://doi. org/10.1007/s00103-023-03770-9) zur Tab. 4 der deutschen Originalfassung ist in dieser Übersetzung implementiert worden.

#### Electronic supplementary material (only online and in German)

Additional electronic supplementary material is available in the online version of the German original recommendation “Hygiene Requirements for the Cleaning and Disinfection of Surfaces” of the Commission for Hospital Hygiene and Infection Prevention (KRINKO) at the Robert Koch Institute (https://doi.org/10.1007/s00103-022- 03576-1) as informative appendix (only in German).

This informative appendix is also attached to this article (see Attachment 1; only in German).

## 1 Introduction

This document updates and expands the 2004 recommendation of the Commission for Hospital Hygiene and Infection Prevention (KRINKO) on hygiene requirements for the cleaning and disinfection of surfaces. To limit the number of references to a minimum, apart from a few exceptions, only sources published after the KRINKO recommendation of 2004 or not included in this recommendation have been included. If statements are based on references in the 2004 recommendation, this KRINKO recommendation is cited as the source. To reflect the complexity of the issue, several new terms have been introduced since the 2004 recommendation (see section 3.1), and a greater distinction has been made between surface cleaning, disinfecting surface cleaning and surface disinfection. At the same time, the evidence basis was reviewed and partially re-evaluated. 

Compared to humans (patients, staff, visitors) as a source of infection and transmission, and as opposed to insufficiently processed medical devices (MDs) as a source of contamination, the significance of microbial contamination or colonisation (biofilms) of inanimate surfaces indoors as a source of nosocomial infections (NIs) has been the subject of less extensive scientific research; in individual cases, the causal link often cannot be demonstrated. This explains the different assessments of the importance of disinfecting surface cleaning or surface disinfection as a standard precaution measure. A paradigm shift has now also taken place in Anglo-American countries [2] due to the increasing awareness of epidemiological associations between the occurrence of pathogens in the patient’s environment, the tenacity and transmissibility of pathogens to patients, and the effectiveness of disinfecting surface cleaning in the context of outbreak management and terminal disinfection. There is international consensus on the necessity of disinfecting surface cleaning according to the indications [3], [4], [5], [6], [7], [8]. This is also reflected in the fact that both re-views and guidelines on the prevention of the transmission of, e.g., *Clostridioides (C.) difficile*, methicillin-resistant *Staphylococcus (S.) aureus* (MRSA) strains and noroviruses, recommend controlled disinfecting surface cleaning as part of the prevention strategy [2], [9], [10], [11], [12], [13], [14], [15], [16], [17], [18], [19], [20], which applies even more explicitly in outbreak situations [21], [22] (see also section 2.3).

### 1.1 Objectives

As an element of standard precaution measures, disinfecting surface cleaning or surface disinfection is intended to prevent nosocomially infected surfaces from becoming reservoirs for pathogens and also limit or eliminate the further spread of pathogens via contaminated surfaces during patient care and treatment [11], [23], [24], [25], [26], [27], [28], [29], [30], [31], [32]. Disinfecting surface cleaning also removes impurities (e.g., blood, secretions, excretions) [33], which additionally contributes to visual cleanliness. If the outbreak involves surfaces, targeted surface disinfection, often as part of a series of measures (bundle strategy), is essential for the rapid termination of the outbreak. For aseptic activities, surface disinfection ensures the required pathogen-free environment [34]. Disinfecting surface cleaning or surface disinfection also contributes to protecting staff health, especially in the case of targeted surface disinfection after potential contamination with pathogens that are relevant to infection, also for immune competent staff.

**Figure 1:**
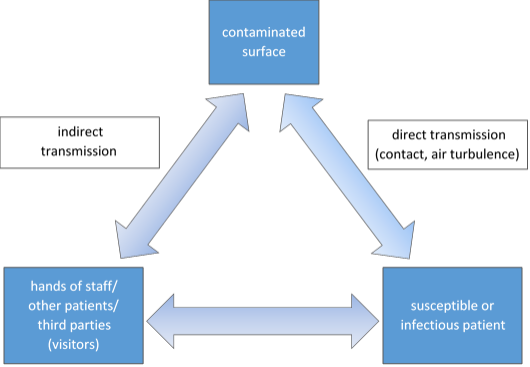
Transmission routes starting from contaminated surfaces

Pathogens may be spread from contaminated areas via the following routes (also see Figure 1 (Fig. 1)):

- Via hands (skin or gloved hands) of staff, other patients and third parties (e.g., visitors) directly to the patient in the event of non-compliance with hand hygiene [35],
- starting from contaminated areas to other areas and from there spread by hand [36],
- through patient contact with the contaminated surface (hands, bare feet when leaving the bed, skin contact, e.g., during diagnostics),
- from clipboards placed on a contaminated surface,
- depending on the pathogen, also aerogenically by air turbulence involving dust and soiled, contaminated surfaces with subsequent sedimentation on other surfaces.

Although hands are the main source of exogenous transmission of NIs, accounting for 5–20% in Europe [37], surfaces can also be a relevant source of contamination [38], [39]. In this respect, hand hygiene [40] and indication-based surface hygiene need to be complementary in terms of standard precaution measures for the prevention of NIs. If the necessity of disinfecting surface cleaning/surface disinfection is unclear in any given case, biocide should be applied after weighing the risk of infection against the risk from handling the hazardous substance.

### 1.2 Scope of application

The following recommendations apply to inpatient and – on a risk-adapted basis – to outpatient health care facilities, including rescue services and qualified ambulance transport, as well as to the nursing and medical care of residents in inpatient-care facilities, but also to areas not used by patients (e.g., processing units for MDs), to eliminate the spread of pathogens (e.g., cleaning rooms, equipment rooms, changing rooms) or in work areas where a low-pathogen environment is required. However, the measures listed can also be applied to specific situations in the home, e.g., in outpatient nursing care, adapted to local conditions.

In ward and milk kitchens as well as in central hospital kitchens, low-pathogen environmental conditions are required; in this case, the requirements of food law apply [41] (for practical implementation, see [42], [43], [44].

### 1.3 Relation to other KRINKO recommendations, the Medical Device law, the Biocidal Products Regulation and to the German list of disinfectants

**KRINKO recommendations:** Surface disinfection, or rather disinfecting surface cleaning, is addressed in almost all KRINKO recommendations, either with respect to surfaces in rooms (work surfaces, furniture, floors, sanitary areas) or the disinfection of surfaces of non-critical MDs (e.g., incubators, monitors, keyboards, device-side operating surfaces, baby scales). Due to the more extensive specific contents on surface disinfection, the following KRINKO recommendations should additionally be referred to: Prevention of surgical site infections (SSIs) [45], infection prevention in the context of the care and treatment of patients with communicable diseases [46], infection prevention requirements for medical care of immunosuppressed patients [47], hygiene measures for *Clostridioides difficile* infections (CDIs) [48], recommendations for the prevention and control of methicillin-resistant* Staphylococcus aureus* (MRSA) strains in medical and nursing facilities [49], hygiene measures for infections or colonisation with multidrug-resistant gram-negative rods [50], hygiene measures for the prevention of infection with enterococci with specific antibiotic resistances [51], and hygiene requirements for waste-water transporation systems in medical facilities [52]. A number of recommendations classify surface disinfection as a standard precaution measure.

**Biocidal Products Regulation (BPR):** As a general rule, surface disinfectants are classified as biocidal products (product type 2) and must undergo an approval procedure, unless they fall under the legal regulations of MDs (see Article 2 of EU Regulation No. 528/2012 [53]). The German Federal Institute for Occupational Safety and Health (BAuA) provides a list of biocidal products that are available on the German market and may be used in Germany in accordance with an ongoing decision-making process [54]. The BPR specifies a two-stage approval procedure for biocidal products, which comprises an active substance approval and a biocidal product approval. With placement of an approved active substance on the “Union list”, the deadline is set by which manufacturers must seek approval of products containing that active substance from the competent authority of a member state or from the European Chemicals Agency (ECHA), if the product is to continue to be marketed. The decisive date for the product approval application is the most recent authorisation date for the active substance included in the respective product mentioned in the “Union list”. This means that a large number of products will still be covered by the current transitional provisions, i.e., without a complete authorisation procedure, for many years to come. The approval process defines requirements for the efficacy and controlled quality of the product. It also requires an assessment of human and animal health risks as well as environmental risks arising from the use of the product. To provide proof of efficacy, primarily European standards of the European Committee for Standardization, Technical Committee (CEN TC) 216 for disinfectants and antiseptics are to be applied, in order to establish a uniform standard in all EU states. The assessment of approved products takes place according to Regulation (EU) No 528/2012 [53] criteria, which are considered as the minimum requirements for biocides and have been developed by the European standardisation bodies. This assessment does not focus on the actual on-site infection risks. According to the standard DIN EN 14885 [55], which is classified under the European standardisation project TC 216, there are no binding specifications for replicating the test results, meaning that one test run with the respective test organism per specified standard suffices. If necessary, it is possible to go beyond the minimum standards of disinfectant approval in terms of efficacy at the national level in accordance with the European BPR [56]. By the same token, it should be noted that the requirements for the state-of-the-art production of disinfectants also exceed the regulatory requirements of the Biocide Regulation.

Irrespective of the type of approval, users can be confident that the efficacy required for use is guaranteed; if necessary, individual examination of expert reports, including the test reports, may provide further indications for use. To confirm that the required efficacy is met, manufacturer-independent lists can be used, as they take the conditions of use into account (see the disinfectant list of the Association for Applied Hygiene e. V. (VAH) in this section for explanations).

**Medical Device law: **Disinfectants may be classified as MDs if their intended use is declared to apply to specified MDs [57], [58], [59]. A disinfectant may also be declared as both a MD and a biocide (dual-use claim). This means that there are several possible requirement profiles. Surface disinfectants declared for the processing of MDs must be approved as MDs and fulfill the requirements of the Medical Device Regulation (MDR). Compliance of the MD with the essential safety and performance requirements is confirmed by the manufacturer with the CE marking and a declaration of conformity. Disinfectants for MDs not only require a declaration of efficacy, but also a declaration of compatibility with the MD and, for example, a declaration of storage stability after opening.

**List of disinfectants of the Association for Applied Hygiene e. V. (VAH, Verbund für Angewandte Hygiene)** **[60]: **This list includes all products that have a valid VAH certificate at the time of publication. This certificate is only issued when the product satisfies the efficacy requirements published by the Disinfectants Commission. This involves the appraisals and test reports being submitted to an evaluation procedure conducted by manufacturer-independent experts. The evidence of efficacy for the respective intended use as well as the stated concentrations and contact times are based on at least two expert opinions, with the associated test reports on investigations based on sound scientific test methods developed by the VAH [61], [62] or the German Association for the Control of Viral Diseases (DVV, Deutschen Vereinigung zur Bekämpfung der Viruskrankheiten e. V.) [63], [64], or test methods that comply with the relevant European standards. In certain instances, the VAH and DVV test methods place higher demands on the proof of efficacy of disinfectants than the European standards, e.g., by testing additional test organisms (see Tab. 7 in the informative appendix to this recommendation, Attachment 1), to ensure that quantifiable results are obtained and record the neutralisation effects of additional dilutions. This is of practical importance for applications in particularly sensitive areas. The tests not only examine the antimicrobial efficacy of the product, but also consider the formulation (e.g., foams) and the application method, if appropriate published test methods are available [65], [66], [67], [68], [69], [70], [71], [72]. Wherever possible, practical tests need to be performed. In the 4-Field Test conducted according to DIN EN 16615 [70] and VAH method 14.2 [61], [62], for instance, effective surface disinfection of a dried contamination is tested by wiping with a disinfectant-soaked cloth. For products used with any non-specified cloth, an effect of the cloth material on effectiveness cannot be excluded. The efficacy tests of the products must be performed and documented in accordance with the requirements of the VAH by testing laboratories that are independent of the manufacturer and whose competence has been demonstrated, e.g., by participation in interlaboratory tests and/or accreditation according to DIN EN ISO/IEC 17025 [73]. The VAH list offers the option of selecting disinfectants based on independent certifications.

**Disinfectant list of the German Society of Veterinary Medicine e. V. (DVG):** The DVG disinfectant list [74] is issued to cover the use of chemical disinfection procedures in the food sector, animal husbandry and veterinary practice. The list for the animal sector also includes antiparasitic products and procedures.

**List of disinfectants and disinfection methods tested and approved by the Robert Koch Institute (RKI):** According to section 18 (1) of the Infection Protection Act (Infektionsschutzgesetz, IfSG) [75], only agents and procedures that are included in the list of disinfectants and disinfection procedures tested and approved by the RKI [76] may be used for disinfection measures ordered by the public authorities. The disinfectant lists of the RKI and VAH differ considerably, particularly with respect to the information on surface disinfection. This can be attributed to the different functions of the lists themselves and, thus, to the different testing methods and evaluation criteria. The VAH list is primarily geared towards routine disinfection, where the RKI list primarily towards officially ordered disinfection (which is generally only used in specific cases, e.g., outbreaks or when dealing with specific pathogens). The basic test methods used for surface disinfectants differ in terms of the type of experimental soiling and test specimens used as well as in the choice of test organisms. The RKI tests are based on experimental soiling of test objects with pathogen-containing coagulated blood. In addition to vegetative bacteria and fungi, including fungal spores, area of action A also generally includes mycobacteria, which generally place higher demands on the disinfectant due to their chemo-tolerance. For many groups of active substances, this results in higher concentration values and/or longer contact time given in the RKI list than in the VAH list. In accordance with the requirements of the RKI, the efficacy testing of the products must be approved by manufacturer-independent expert opinions in testing laboratories whose competence has been demonstrated, for example, by participation in interlaboratory tests or accreditation. The RKI also conducts practical efficacy tests.

**Disinfectants compiled by the Industrial Association for Hygiene and Surface Protection (IHO, Industrieverband Hygiene und Oberflächenschutz e. V.):** The IHO disinfectant list is a compilation of disinfectants for a range of applications with details on efficacy, but the entries are only listed by the respective company name and under the sole responsibility of the company.

**Summary of the listing of disinfectants:** In the future, the efficacy testing of disinfectants will be performed as part of the approval process in accordance with the European Biocide Regulation (Regulation (EU) 528/2012 [53]), e.g., in accordance with DIN EN 14885 [55]. As mentioned above, the primary objective of product approval under the harmonised BPR is not to ensure the highest possible level of protection against infection.

As stated in the explanatory notes on product type 2, the area of application includes but is not restricted to swimming pools, aquariums, bath water and other types of water, as well as air-conditioning systems, walls and floors in both private, public and industrial areas and in other areas of professional activity. Medical facilities are not explicitly mentioned. Instead, the main purpose of the regulation is to prevent unnecessary risks to humans or the environment. As a result, the efficacy demands of disinfectants under biocide legislation do not satisfy the requirements for disinfectants intended to ensure the highest possible level of protection against infection in medical facilities. The approval of a surface disinfectant based on a group test (product family), which is feasible under the BPR, is not adequate for surface disinfectants destined for medical areas, because product efficacy is not only defined by its active ingredient but may also be affected by additives such as surfactants and pH. As a prerequisite for certain efficacy, the bactericidal, levurocidal, tuberculocidal, mycobactericidal, fungicidal, sporicidal and/or virus-inactivating efficacy required for the respective medical field of application must be confirmed by two independent test reports and expert opinions. These test reports and expert opinions must reflect the current state-of-the-art, consider new forms of application, and be issued by manufacturer-independent, accredited testing institutes, as practiced by the VAH, for example. Nevertheless, regular post-testing of products on the market by an independent institution provides additional certainty about the product’s efficacy. This is why independent disinfectant lists, such as those of the VAH and the RKI, will remain important.

## 2 Risk assessment of surfaces and prevention potential of surface cleaning, disinfecting surface cleaning and surface disinfection

### 2.1 Risk differentiation of near-patient (high-touch) and patient-remote (low-touch) surfaces

The potential importance of surfaces as a reservoir and/or source of transmission of pathogens depends on the pathogen load of the colonised and/or infected patient and the associated shedding of pathogens into the environment, as well as the quantity, virulence, resistance and tenacity of these pathogens in the environment, in addition to the infectious dose, route of infection and immunocompetence of the patient. Non-patient sources (e.g., spread of mould through building dust) can also contribute to surface contamination. Disinfecting surface cleaning is particularly pertinent in the environment of infection-prone and immunocompromised patients.

The decision whether cleaning is sufficient or disinfection measures are required is determined by

- the probability of microbial contamination,
- the potential for shedding pathogens where the differing patient risks result from the colonisation, suspected infection or infection of the patient,
- the probability of staff or patients being directly contaminated from the surface,
- the requirement for a pathogen-free environment during aseptic activities (e.g., preparation of infusion solutions, enteral nutrition solutions, provision of injection equipment, preparation of medicinal products in the pharmacy, operating theater),
- the patient’s susceptibility to infection, e.g., immunodeficiency due to an immature immune system, chronic diseases or immunosuppression,
- the risk to staff posed by pathogens.

For example, the literature describes a case in which a genotypically identical Serratia liquefaciens strain was spread from a siphon to a clean work surface (among other places), and, due to a lack of or inadequate disinfection of work surfaces and hands, was in turn transferred to two infusion bottles, resulting in sepsis [77]. Both in the reconstitution of the parenteral route [78] and in the manufacture of non-sterile prescription medicinal products, the disinfection of work surfaces is mandatory [79].

When assessing the risk, it is particularly important to consider surfaces that come into direct contact with the skin (especially hands), mucous membranes or wounds of patients and staff that are contaminated with secretions and excretions, or that may be contaminated by air turbulence. Pathogens may be transmitted from these surfaces indirectly (e.g., via hands, nursing aids) or via dust and active turbulence to patients or infection-relevant surfaces and MDs/instruments (e.g., in the operating theater) (see Figure 1 (Fig. 1)).

In this context, **near-patient surfaces** that often come into contact with hands/skin (frequently touched surfaces), and that may often become contaminated, are associated with a higher risk of transmission than surfaces that are further away from the patient (infrequently touched) and which staff also do not usually touch. Near-patient surfaces include contact parts of the bed and accessories, bedside table, patient sanitary area (bathtub, washbasin and surroundings, taps, toilet), door handles, remote controls, nappy changing tables, examination couches as well as device surfaces or non-critical MDs (e.g., incubators, ECG device and accessories) [20], [80]. When operating equipment, the risk of a potential cross-contamination of patient-side surfaces and equipment-side operating surfaces needs to be considered.

Surfaces on which aseptic activities are performed must be disinfected immediately before the activity to prevent contaminating low-germ or sterile products. This not only applies to the work surfaces of the dressing trolley but also to the clean work surfaces used to prepare infusion solutions and syringes. For practical reasons, disinfectants with the shortest contact time, e.g., one minute (min), are preferable.

Surfaces that are frequently disinfected (e.g., clean work surfaces) typically exhibit a relatively low total number of colony-forming units (CFUs) when tested by contact culture (referred to as the “smear” method). Conversely, frequently touched surfaces that are not intermittently disinfected, e.g., PC keyboards, boxes for removing mouth/nose protection and pathogen-free disposable medical gloves, doorknobs, etc., are often found to harbour ≥20 CFU/cm^2^, including potential nosocomial pathogens, in contact culture [81], [82], [83]. The rapid advances in next-generation sequencing (NGS) techniques have also made it possible to capture the hospital microbiome. The hospital microbiome refers to the sum of all microorganisms that can be detected on the surfaces of the different areas of the rooms of a hospital. Compared to contact cultures, NGS is able to identify the genetic material of many more species. This potentially allows detection of microorganisms that cannot be cultured. However, NGS cannot determine whether microorganisms were capable of reproducing at the time of sampling [84], [85], [86]. Preliminary studies indicate that environmental monitoring supplemented by the assessment of the indoor microbiome in hospitals may open up future opportunities to derive control strategies based on specific characteristic compositions of the microbiome on the different surfaces (see section 6.3).

Even **patient-remote surfaces** that are not in frequent contact with hands or skin (“low-touch”) need to be included as a source of contamination according to an infection hygiene risk analysis, and must be cleaned and disinfected, particularly in the case of visible contamination, if patient-remote surfaces are not included in an outbreak event [87]. Such surfaces include walls (beyond the direct contact area of the patient’s bed), floors in areas without increased risk of infection, ventilation outlets (including exhaust vents), lamps and radiators. Especially in the case of non-aerogenic pathogens or pathogens that can be transmitted via air turbulence, the importance of disinfecting surface cleaning of patient-remote surfaces to prevent infections decreases with increasing distance from the patient [88].

### 2.2 Occurrence and tenacity of nosocomial pathogens in the patient environment and reduction of the pathogen load through disinfection measures

Particularly in near-patient environments of microbially colonised or infected patients, the etiological species causing the colonisation or infection can only be detected if no surface disinfection has been carried out. This also applies to viruses, specifically enteroviruses [89], noroviruses [90], [91] and SARS-CoV-2 [92], as well as bacteria, e.g., MRSA [93], [94], vancomycin-resistant enterococci (VRE) [93], [95], carbapenem-resistant *Enterobacteriaceae* (CRE) [96], [97], *Acinetobacter* spp. [91], *C. difficile* [91], [98], fungi and yeasts [99], and in rare instances also dermatophytes [100], both on near-patient surfaces and on hand and skin contact surfaces as well as on floors [101], [102]. Even *Acanthamoeba*, *Vahlkampfia* and *Vermamoeba* spp. have been detected in the dust of internal medicine intensive care units (ICUs) as well as on equipment, doors and in the air-conditioning system of surgery and open-heart surgery ICUs [103]. *Aspergillus* spp. are primarily released during renovation work [104].

The tenacity of microorganisms or viruses affects the risk of direct or indirect spread of pathogens, while the minimum infection dose determines the risk of disease. Both Gram-positive and Gram-negative bacteria can survive for days to weeks or months, depending on the environmental conditions and the pathogen load; bacterial spores can sometimes survive much longer. The tenacity of viruses is often lower, with large differences between enveloped and non-enveloped viruses (for details on infectious dose and tenacity, see informative appendix, Attachment 1).

The reduction of microbial surface contamination by disinfecting surface cleaning has been demonstrated in various medical settings [105], [106], [107], [108], [109], [110], [111], [112], [113].

### 2.3 Infection epidemiological studies

There is a growing body of evidence on the association between environmental contamination and the risk of infection. Several studies have shown that when patients who were colonised or infected with specific pathogens were discharged, subsequently admitted patients contracted the same pathogen if there were deficiencies in terminal disinfection [23], [26], [114], [115], [116], [117], [118]. This has also been confirmed in meta-analyses [119], [120]. However, none of the studies examined provide direct evidence that the previous and the new patient were colonised with the same clone of the respective species, which means that this association may only be considered as highly probable. Similarly, environmental VRE contamination has been identified as an independent risk factor for contracting VRE in both an internal medicine ICU and a post-acute care setting [121], [122]. Results from one review indicated that both outbreaks and sporadic infections could be attributed to contaminated near-patient surfaces [123].

In a cohort study (n=82) investigating risk factors of household infections with community acquired MRSA (CA-MRSA), 65% of patients contaminated with environmental CA-MRSA in the household suffered a recurrent infection, whereas this was only observed in 35.5% of patients from households in the absence of environmental contamination. Environmental contamination significantly increased the risk of recurrent infection [124].

In a surgical ICU, the presence of *S. aureus* on near-patient hand-contact surfaces tended to be associated with a higher rate of NIs. The fact that *S. aureus* genotypes isolated from patients and the environment cannot be distinguished from one another in addition to the presence of temporal dependencies argues for transmission in both directions [125].

To date, only a few controlled clinical studies have examined the efficacy of disinfecting surface cleaning, in the context of standard precautions, with infection as an endpoint. Intensified disinfecting surface cleaning reduced surface contamination by 94% and was found to result in a 35% reduction in colonisation and/or infection of patients with MRSA, VRE, *C. difficile* and multidrug-resistant *Acinetobacter* spp. [126]. Most of the investigations were initiated in relation to a CDI. A before-and-after study design was used to investigate the impact of the disinfection on the rate of CDI. By switching from a chlorine-based surface disinfectant to cloths containing peracetic acid (PAA), the rate of CDI was reduced from 6/1,000 patients to 2/1,000 patients [25]. Changing from a quaternary ammonium compound (QAC)-soaked cloth to hypochlorite disinfection reduced the rate of CDI by 85%, from 24.3 to 3.6/10,000 patients [127]. In two other before-and-after interventional studies, the incidence of CDI was reduced only at relatively high endemic rates after substituting a QAC for hypochlorite; it then increased again after reverting to a QAC, and was once again reduced upon switching to hypochlorite [128], [129]. Hacek et al. [130] demonstrated a significant reduction in the incidence of CDI even at low endemic CDI rates after switching from a QAC to hypochlorite. A multicenter study of 16 hospitals in Ohio (USA), which were either randomised as intervention or control hospitals, examined the impact of an intensified surface disinfection program on the incidence of CDI. The intervention consisted of improving surface disinfection by training, performing quality controls with fluorescent dye, and providing feedback on the results. Intervention was associated with an improved quality of disinfection in the intervention hospitals but had no impact on the rate of nosocomial CDI. The publication does not specify which disinfectants were used, which means that the lack of effect can only be suspected to be due to insufficient sporicidal efficacy [131].

A cluster randomised cross-over multicenter study showed a significant reduction in new colonisations or infections caused by multidrug-resistant (MDR) pathogens when sodium hypochlorite was used instead of a QAC. A combination with UV irradiation did not demonstrate additional benefits [132]. A before-and-after study demonstrated a significant reduction in the incidence of MRSA after switching from a two-step procedure (see section 3.1 for definition), i.e., cleaning followed by wipe disinfection with propan-2-ol, to a one-step procedure (see section 3.1 for definition) with a QAC-based disinfectant cleaner [133]. The two-step procedure described in this paper presumably resulted in an inadequate disinfection effect, perhaps due to residual moisture and dilution of the propan-2-ol or because of excessively rapid evaporation and inadequate wetting of the surfaces.

To control outbreaks involving VRE [134], [135], *C. difficile* [136], MRSA [106], *Acinetobacter (A.) baumannii* [137], [138], [139], [140] and multiresistant Gram-negative bacteria (MRGN) [134], [141], intensified surface disinfection proved to be an effective measure in the bundle of interventions. In addition, several of the studies examined also demonstrated the reduction of pathogen-specific surface contamination through surface disinfection [105], [107], [136].

### 2.4 Conclusions

Disinfection of near-patient surfaces has the potential to prevent NIs. To date, this has predominantly been demonstrated for *C. difficile* and multidrug-resistant organisms (MDROs; e.g.,* Klebsiella pneumoniae*, VRE) and also as part of bundles of measures deployed to control outbreaks [142]. This is why international guidelines assign a key role to the disinfection of near-patient surfaces and surfaces that involve frequent hand/skin contact, in the context of standard precaution measures, to interrupt the spread of critical pathogens and prevent NIs in general [143], [144], [145], [146].

Based on the above, and analogous to hand antisepsis, there are 5 indications for surface disinfection [147]:

- disinfecting surface cleaning of near-patient surfaces as part of standard precautions (non-targeted surface disinfection) in the context of care/treatment, in particular for frequently touched surfaces,
- targeted disinfecting surface cleaning or surface disinfection after contamination with potentially pathogen-containing material,
- surface disinfection before aseptic activities on the work surface,
- terminal disinfection,
- disinfecting surface cleaning as part of a bundle of measures to control outbreaks.

## 3 Surface cleaning, disinfecting surface cleaning and surface disinfection

### 3.1 Definitions

**Surface cleaning:** Cleaning processes are intended to remove impurities (e.g., dust, dirt, organic substances such as blood, secretions, excretions) using water with cleaning-enhancing additives (e.g., surfactants). This process also removes microorganisms mechanically without actually or intending to kill/inactivate them. However, there is currently no valid test method for quantifying cleaning, which means that there is no available data on the cleaning effect of cleaning agents.

Depending on the amount of contamination, cleaning is required before disinfecting surface cleaning or surface disinfection (**two-step procedure**).

In addition to maintenance cleaning (cleaning as part of standard precautions), there are additional cleaning and care measures, e.g., care film renewal, basic cleaning and floor care, which are not covered in the current recommendations [148].

**Disinfection:** Disinfection is a process that reduces the reproductive microorganism count to a level assumed to be harmless in terms of infection hygiene by killing/inactivation, based on the latest standardised, quantifiable evidence of effect in accordance with the most up-to-date knowledge, with the objective of converting the condition of an object/area into one that no longer poses a risk of infection. This applies to both disinfecting surface cleaning and surface disinfection. The basic requirements for the efficacy of surface disinfectants are not based on epidemiology and are therefore only a guide.

**Disinfection procedures as part of standard precautions (also referred to as routine disinfection or current disinfection):** These serve to reduce surface contamination, without any indication that specific pathogens have been released, where the additional elimination of these pathogens would require an extended spectrum of activity.

In ***surface disinfection***, the surface is disinfected by using a surface disinfectant without requiring any additional cleaning effect (**one-step procedure**). The main area of application is the disinfection of work surfaces before aseptic activities, e.g., drawing up syringes or handling parenterals.

In the case of **disinfecting surface cleaning**, cleaning and disinfection are carried out in one operation (**one-step procedure**). This is to limit or prevent the spread of pathogens during patient care and treatment. It also applies to areas that are suspected or assumed to have been contaminated with pathogen-containing material without this being apparent in individual cases. Disinfection after the patient is discharged/transferred is intended to prepare the area/room to be used for the care or treatment of the next patient without posing any risk of infection.

These two procedures should only be carried out in one step if the surface is not heavily soiled (see section 5.1).

For disinfecting surface cleaning, products containing both a cleaning additive and a disinfectant agent are used. Cleaning agents and disinfectants must not be mixed unless specified by the manufacturer, because of potential incompatibilities of the ingredients (mixing prohibition).

**Targeted surface disinfection:** This requires the use of surface disinfectants with a specific indication.

- **Surface disinfection with specific activity spectrum:** This is performed in cases of patients who shed pathogens and therefore a specific activity spectrum for inactivation of the pathogen is required, e.g., tuberculocidal, sporicidal, fungicidal or virucidal activity. It is primarily used in outbreak situations for disinfecting surface cleaning during treatment or care of isolation patients or cohort patients, and for terminal disinfection after lifting the isolation period (end of infectiousness) or after discharge of the patient.
- **Disinfection after contamination with potentially pathogen-containing material:** After contamination with blood, secretions or excretions, a high load of pathogens and/or organic material is to be expected. This type of contamination must be immediately removed by mechanical means (do not use disinfectants because they fix proteins). Disinfecting surface cleaning or surface disinfection (labelled as “dirty”, see section 5.1) should only be carried out after this initial step (**two-step procedure**).
- **Terminal disinfection: **The facility-specific risk assessment must determine which pathogens require terminal disinfection. Depending on the pathogen, terminal disinfection may extend to near-patient surfaces or all accessible surfaces and objects that are potentially contaminated with the pathogen. In specific instances, other concentration:time ratios and procedures may be required than those used for surface disinfection in the context of standard precautions, e.g., in the case of officially ordered disinfection with products or procedures from the list of disinfectants and disinfection procedures tested and approved by the RKI [76].

According to both § 23 IfSG and § 137 SGB V, in-house procedures for infection hygiene must be laid down in hygiene plans. The Technical Rules for Biological Agents (TRBA), in particular TRBA 250 “Biological Agents in Health Care and Welfare Facilities” [149], defines this requirement more precisely. Accordingly, the cleaning and disinfection plan for all surfaces of the hygiene plan should specify when, with what and how these surfaces are to be cleaned or disinfected, and also include information on reuse (see section 8.2).

### 3.2 Effectiveness of surface cleaning and disinfecting surface cleaning

Cleaning may deplete pathogens but is generally not sufficient to kill or inactivate them. Only a few sensitive pathogens are susceptible to inactivation by surfactants [150]. This is why cleaning measures alone are not sufficient in health care facilities when disinfecting surface cleaning or surface disinfection is indicated.

The 4-Field Test provided experimental evidence that disinfecting surface cleaning achieved a significantly greater reduction of *S. aureus* compared to the control, which used water with an added surfactant [151], [152]. In contrast to disinfecting surface cleaning, there is no evidence (see section 2.3 for available evidence) that surface cleaning reduces the rate of NI. Significantly more nosocomial pathogens were detected on surfaces after using cleaning solutions than when disinfectant solutions were used [153].

## 4 Cleaning and disinfection measures in different risk areas

### 4.1 Assignment of rooms to risk areas

As described in the previous sections, the decision as to whether cleaning or disinfecting surface cleaning should be carried out depends on a number of factors that may affect patients, staff and processes. For the purposes of standardising the cleaning and disinfecting surface cleaning process to the greatest possible extent, it has proven practical to divide rooms and surfaces into different risk areas (Table 2 (Tab. 2)).

### 4.2 Information on risks when applying surface disinfectants

On a facility-specific basis, the hygiene staff are to establish the extent, activity spectrum, contact time and frequency of surface disinfection in the cleaning and disinfection plan, in accordance with TRBA 250 [149] and based on the hygiene plan (see section 8.2), as a function of the risk area and risk surfaces (Table 3 (Tab. 3)). Outpatient facilities that have neither a hygiene control officer nor IPC link personnel must define and assume responsibility for infection prevention via the providers or directors of these facilities according to section 23 (3) of the IfSG [75]. The plan must take the epidemiological evidence for infection control in the area of application and the risks arising from biocide use, as well as the Chemicals Act [154] and the Ordinance on Hazardous Substances [155] into consideration. Although contaminated door handles and keyboards, for instance, have been the cause of outbreaks [156], [157], disinfection cannot be carried out after every contact. Therefore, such infection routes must be interrupted in accordance with the multi-barrier concept by hand antisepsis, personal protective equipment and distancing working methods (non-touch technique). In contrast, surfaces that come into contact with the skin of different consecutive patients (e.g., contact surfaces of patient couches, headrests, baby scales) must be disinfected after each use.

## 5 Requirements for surface cleaning and disinfection procedures and selection criteria

### 5.1 Efficacy and activity spectrum

Cleaning procedures are intended to remove soiling by purely mechanical means (see section 1.3). However, there are no requirements for the cleaning effect in terms of reducing the number of microorganisms.

In contrast, for surface disinfection procedures, the requirements for efficacy are defined by European standards and national test methods [61], [62], [63], [64], [65], [66], [67], [68], [69], [70], [71], [72], [73] (for test methods, see informative appendix, Attachment 1, section 5). Disinfection should achieve a ≥5 log_10_ kill rate of vegetative bacteria on the surface. For yeasts, moulds, mycobacteria, *C. difficile* spores and viruses, the requirement is set at ≥4 log_10_ [61], [62], [63], [64]. These requirements consider the worst-case scenario of surface contamination in health-care facilities because bacterial contamination on near-patient surfaces is only in the range of 30 CFU/cm^2^ [158]; even in public toilets, 10^3^ CFU/cm^2^ is not exceeded, and is usually below 10^2^ CFU/cm^2^ [159]. The test of efficacy thus considers the application conditions of surface disinfectants by wiping or – in the case of minor soiling – by spraying. In the VAH listing, the corresponding suitability for and efficacy of the respective procedure are shown based on the protein (protein error) and blood (blood error) load with reference to test methods that are similar to the application. It is important to bear in mind that the protein error varies among the disinfectant agents. For instance, QACs and alcohols have a more pronounced protein error than do aldehydes. Oxidants have a low protein error but a high blood error [160]. For the declaration of efficacy on visually clean surfaces (“low load/clean conditions”), the test is performed with test soiling of 0.03% albumin, and in the case of visible contamination (“high load/dirty conditions”), with test soiling of 0.3% albumin and 0.3% sheep erythrocytes. For visually clean surfaces, products should be labelled “clean”; for visibly soiled surfaces, products should be labelled “dirty”. For heavy soiling, the two-step procedure with cleaning and subsequent disinfecting surface cleaning is to be used instead of the one-step procedure using disinfectants labelled for “dirty conditions”.

The activity spectrum for surface disinfection must include vegetative bacteria (bactericide) and yeasts (levurocide) as a basic requirement. Depending on the pathogen, additional pathogens such as *Mycobacterium* *(M.) tuberculosis* (tuberculocidal), atypical mycobacteria (mycobactericidal), bacterial spores (sporicidal), moulds (fungicidal) and/or viruses (products with limited virucidal, limited virucidal PLUS or virucidal activity) may also need to be inactivated. The declaration of the activity spectrum is based on the required efficacy against the test organisms specified in the test standards (see informative appendix, Attachment 1, Tab. 7). If the VAH or RKI lists do not include any products that are effective against *C. difficile* and/or viruses, the product selection for these areas of activity may be evaluated based on the plausibility and congruence of the expert opinions and test reports (at least two from independent, accredited test laboratories) in line with the methods published in Germany. If a pathogen is known to have a higher chemoresistance (e.g., such as *Candida (C.) auris* [161]), a recommendation for use can be derived, if necessary, by evaluating currently available evidence.

In selecting the surface disinfectant, a careful risk-benefit analysis based on the activity spectrum and the compatibility profile (human, animal, environment, material) (see informative appendix, Attachment 1, section 3) must be performed. The manufacturer’s product information is to be used for this purpose; it provides details on the activity spectrum, application concentration and contact time.

Indicative data on the activity spectrum of microbicidally active substances or substance classes for surface disinfection are given in Table 4 (Tab. 4). It should be noted that this table only provides a guide, because commercial products are generally combinations of several active substances which, together with the specific type of formulation, may influence efficacy. This is why the efficacy documented in expert opinions is decisive, whereas the tolerability can only be estimated, with the individual active substances contained providing orientation, as combination effects remain to be determined.

### 5.2 Contact time

The killing/inactivation of microorganisms follows characteristic kinetics depending on the type of active substance, the amount of disinfectant applied and the concentration [162]. In laboratory tests, large quantities of test organisms (10^6^ up to 10^8^) are used for methodological reasons to enable the required reduction to be measured. These high pathogen counts only occur in visible contaminations or in biofilms. Generally, surfaces are significantly less soiled in practice. In addition to the disinfection effect, wiping the surfaces also mechanically removes pathogens. Results from practical tests of surface disinfectants ensure that even greater numbers of pathogens are killed/inactivated at the indicated contact time. The smaller the amount of disinfectant applied, the greater the risk of non-wetted surfaces.

When disinfecting surfaces, and depending on the risk of infection (see Table 2 (Tab. 2)) and the level of patient safety required, it is important to evaluate whether the surface can be used after drying or whether the contact time should be observed before use. This is because the killing/inactivation of the pathogens is not linear, but logarithmic. Consequently, depending on the type of use, surfaces can in the majority of cases be used or walked on after air drying, provided the specific characteristics/conditions outlined in Table 3 (Tab. 3) are observed. This approach is warranted if there is no visible/massive contamination (low pathogen load), a sufficient amount of disinfectant is applied by mechanical means (wiping), and the application conditions are derived from practical tests and not from suspension tests. Suspension tests are still often employed to deterimine efficacy against viruses or bacterial spores, because practical tests usually involve higher concentrations and/or longer contact time. Insofar as the 4-hour value is to be applied, given the low concentration, there is a risk that pathogen reduction will not yet be sufficient by the time the surface dries, which means that the surface cannot be considered disinfected at this stage.

The operating theater is a very particular area because it encompasses different contamination risks depending on the surface (aseptic surfaces, frequently touched or near-patient surfaces, and rarely touched or patient-remote surfaces). While the instrument table as a surface for aseptic activities should not be used before the contact time period has been completed, other surfaces can already be used after drying. Whether the next operation (incision) can begin before the end of the contact time needs to be determined with the help of a hospital hygienist, as part of the risk assessment. Aspects such as contamination of the operating theater floor, air conditioning, type of operation, etc. must be considered. Depending on the surface, disinfectants with a very short contact time can be selected, e.g., contact time of 1 min for instrument tables or ≤5 min for floors.

In addition to the specifications given in Table 3 (Tab. 3), waiting for completion of the contact time is required in the following situations:

- in all cases where the applied product is rinsed off with drinking water, thereby terminating the disinfection process, e.g., in the ward kitchen or in patients’ bathtubs,
- if efficacy against viruses or bacterial spores has only been demonstrated in suspension tests.

### 5.3 Preventing the spread of pathogens

Cleaning solutions in particular, but also to some extent disinfectant solutions involving the re-immersion of the wiping textile after wiping surfaces, are rapidly contaminated with pathogens such as *Pseudomonas (P.) aeruginosa*, *Enterobacteriaceae* and *Acinetobacter* spp. [153], [163], [164]. The application of contaminated solutions results in the further spread of microorganisms on subsequently mopped surfaces and may be associated with outbreaks [165]. Cleaning and disinfection procedures must therefore be organised and carried out in such a way that they do not increase the microbial load and spread pathogens on surfaces (see section 6.1.1). The re-immersion of used wiping textiles in particular is not permitted [148]. If nosocomial colonisations and infections increase, incorrectly performed cleaning and disinfection procedures must also be considered as a source of infection.

Wiping textiles intended for repeated use must be machine processed in such a way that no pathogens are detected after processing and there is no subsequent microbial propagation (no residual moisture). Visual cleanliness must be achieved at the same time. Otherwise, the disinfectant may be inactivated by remaining impurities and lose its effectiveness. This may lead to the development of tolerance or resistance [166]. It should be noted that when pre-soaked wiping textiles are left to stand for a long time, this too may result in microbial propagation [148].

### 5.4 Prevention of selection and development of resistance

Depending on the mode of action of specific microbicidally active substances, these may lead to the development of resistance, in some cases even cross-resistance to antibiotics – a factor that has to date received too little attention when selecting surface disinfectants.

Among the disinfectants used for surface disinfection, the development of resistance has so far only been demonstrated for QACs, e.g., for benzalkonium chloride (BAC) and didecyldimethylammonium chloride (DDAC). At the genetic and molecular level, the underlying resistance mechanisms may be elucidated, e.g., by detecting non-specific multidrug efflux pumps [167], [168], [169], [170], [171], [172]. This also explains the simultaneous increase in resistance to certain antibiotics. Resistant staphylococcal strains with cross-resistance to antibiotics were only detected in isolates obtained from surfaces disinfected with BAC-soaked cloths or BAC sprays and not from surfaces that were not disinfected with BAC [170]. A *P. aeruginosa* strain that underwent passaging *in vitro* increased its resistance to BAC from 0.02 to 0.36% [173]. If, for instance, BAC used as a monoactive substance reaches or is diluted more than the 4-hour-concentration threshold, a weakening of the effect in practice cannot be ruled out. It remains to be investigated whether this is also the case when used in combination with other microbicidal active substances or when BAC is used together with a basic additive to achieve a high alkaline pH value, which results in virucidal efficacy. For DDAC, BAC and cetyltrimethylammonium chloride, an adaptive increase in resistance can be induced by passaging at sublethal concentrations [174], [175]. If DDAC is used long term at growth-inhibiting rather than microbicidal concentrations, species with increased antibiotic resistance can be selected [176]. There was also evidence of an adaptive development of resistance to the combination of DDAC with 2% propan-2-ol in *P. aeruginosa* strains which was possible to reverse by increased concentrations of the active substances or a longer contact time [177]. To conclude, it may be deduced that particularly for QAC-based surface disinfectants, the active substance should be used at concentrations that are effective against microbicidal and levurocidal activity. In suspension tests, the lowest effective BAC and DDAC concentration was found to be 0.005%. In the event of an MDR-strain outbreak, surface disinfectants based solely on QAC should not be used, as it is impossible to predict the sensitivity of antibiotic-resistant strains to QAC [178].

Resistance has not been reported to develop against alcohols, glucoprotamine, aldehydes, PAA, peroxides, chlorine dioxide and hypochlorite. Compared to chloramine B (diluted 1:250), the minimum inhibitory concentration (MIC) increased significantly, while the minimum bactericidal concentration (MBC) remained unchanged, i.e., the application concentration was unconditionally effective [179].

### 5.5 Risks to humans and the environment

The active agents used to disinfect surfaces differ significantly in terms of their risks to humans and the environment [180], [181] (see also informative appendix, Attachment 1, section 3). Due to their microbicidal mode of action, disinfectant agents require careful toxicity and ecotoxicity evaluation to minimise side effects for humans and the environment as far as possible. This is addressed, among other things, by the requirements of the Technical Rules for Biological Agents (TRGS), in particular TRGS 525 [182], and the TRBA 250 recommendations [149] on occupational health and safety. More details are provided in the recommendations of the German Social Accident Insurance (DGUV), the International Social Security Association (ISSA) [183], [184], [185] and in the informative appendix (Attachment 1) to the current recommendation. Independent of this, the toxicity and ecotoxicity assessment is part of the biocide authorization procedure.

A minimization principle applies to the handling of hazardous substances, which states that the use of disinfectants must always be objectively justified and minimised as far as possible [154], [155]. Although the EU authorization allows the unrestricted marketing of active substances listed below in terms of their toxic risks, the informative appendix (Attachment 1) provides suggestions from a risk-assessment perspective, in the interest of protecting health. The informative appendix (Attachment 1) therefore gives suggestions on potential side effects of selected active agents for humans and the environment and lists alternative active agents with the same activity spectrum. However, only active agents whose risks have not been assessed as part of the biocide product authorization are considered.

**Short-chain aliphatic alcohols:** Due to their flammability, alcohols should only be used on small, delimited surfaces (max. 50 ml/m^2^ [182]). Any possibility of sparks from electrical equipment or electrostatic charge must be excluded. Exposure by inhalation does not pose a health hazard [186], [187]. The material compatibility must betaken into account, as some plastics are susceptible to damage. It should be noted that open containers may release active substances by evaporation.

**Aldehydes:** Aldehydes should be rejected for toxicological reasons, particularly for surface disinfection, but also for room disinfection, as more compatible alternatives are available. The main hazard is their allergenic potency with cross-sensitization occurring between aldehydes. For formaldehyde, the threshold concentration for sensitization is 0.3%, and for triggering an epicutaneous reaction 0.05% but in extremely rare cases <0.05%. Long-term inhalation exposure to formaldehyde increases the risk of chronic obstructive pulmonary disease (COPD) [188]. The WHO (2010) recommendation for indoor environments, which has been adopted by the German Federal Environment Agency, applies to patients and work areas where formaldehyde is not normally handled. The formaldehyde concentration should not exceed 0.08 parts per million (ppm) (0.1 mg/m^3^) at any time or in any 30-minute period [189]. In workplaces where formaldehyde is handled, an occupational exposure limit (OEL) of 0.3 ppm (0.37 mg/m^3^) applies; for 4×per shift, a concentration twice as high is permissible for 15 min at one hour intervals [190]. Both specifications are designed to prevent irritant effects as well as cancers induced by tissue damage.

Room fumigation with formaldehyde is no longer carried out in hospitals, except for special isolation wards, or as part of patient transport, but only to avert the risk of exceptional infectious events. If disinfectants containing formaldehyde are to be used in disinfection measures ordered by the authorities, occupational safety measures must be taken to exclude any risks. Alternatively, nebulization of hydrogen peroxide (H_2_O_2_) should be considered.

Glutaraldehyde is also released into the ambient air when used for disinfection. The OEL for handling glutaraldehyde is 0.2 mg/m^3^ (0.05 ppm); for 4×per shift, a concentration twice as high is permissible for 15 min at one hour intervals [190]. As the odor threshold is 0.04 ppm, there is an inhalation risk if odor is perceived [191]. Measurements in different workplaces in the hospital found values of up to 0.08 ppm. In endoscopy units, provocation tests confirmed the association between exposure to glutaraldehyde and the triggering of asthma and rhinitis [192].

**Aliphatic carboxylic acids:** Organic carboxylic acids (e.g., lactic acid and formic acid) for surface disinfection are either used pure or in combination, e.g., with QAC, to reduce the QAC concentration. They pose no toxic risks at their application concentrations and are environmentally safe [193]. In addition, they also remove limescale.

**Peroxides:** When using H_2_O_2_ for room disinfection, due to the instability of H_2_O_2_ the room can be accessed after the end of the decay time which is dependent on the procedure used (OEL: 0.5 ml/m^3^ or 0.7 mg/m^3^). Patients may only access the room when the H_2_O_2_ concentration has fallen below the derived no-effect level (DNEL) for acute inhalation exposure of the general population, which is set at 1.93 mg/m^3^ [194]. Modern technologies monitor the ambient H_2_O_2_ air concentration during nebulization until the room is cleared for access. If colloidal silver in the ppm range is added to the H_2_O_2_ as a catalyst, an inhalation risk cannot be ruled out due to the stability of colloidal silver even after H_2_O_2_ has fallen below the detection limit.

**Peracetic acid (PAA):** Analogous to peroxides, PAA is not allergenic. However, if inhaled, PAA (2.4 ppm) is neurotoxic [195]. The inhalation of PAA, even at relatively low concentrations, has an irritant effect; according to the ECHA, the DNEL for occupational safety is 0.56 mg/m^3^ and 0.28 mg/m^3^ for the general population [196]. Odor nuisances may be expected even below these concentrations; good ventilation is required. PAA is particularly corrosive to copper and its alloys.

**Chlorine-releasing compounds:** Hypochlorite and hypochlorous acid are slightly toxic, but are perceived as odor nuisances and respiratory irritants depending on their concentration, which is why they are hardly used in Germany, in contrast to Anglo-American countries or southern Europe. If hypochlorite and hypochlorous acid are handled in strongly alkaline formulations, potential health hazards must be considered. When mixed with acidic solutions, life-threatening chlorine gas emissions may occur. Hypochlorites are not compatible with some surfaces (e.g., aluminium). Sodium hypochlorite very rarely induces sensitization. Tosylchloramide sodium (chloramine T) is a weak allergen; if it is inhaled as dust, allergic respiratory diseases may be triggered in some isolated cases [160].

A problem for all chlorine-releasing compounds, including chloramine T, is that they form adsorbable, organically bound halogens (AOX), which are toxic, biologically poorly degradable with mutagenic and carcinogenic potency [197], which often lead to threshold values being exceeded in hospital wastewater. However, the largest source of AOX is organic iodine compounds in X-ray contrast media [198], [199]. Where sporicidal action is required, oxidants are preferable to chlorine-releasing compounds for large-scale use, in order to reduce the contamination of wastewater with AOX.

**QAC:** The large-scale, regular application of QAC has not been sufficiently characterised in terms of toxicology. When applied to floors, a visible accumulation develops which cannot be removed using normal cleaning methods. When applying disinfectants, a proportion of the QAC adheres to aerosols or dust particles and – when these are disturbed, e.g. by walking on surface-disinfected areas – dry QAC may detach and find its way into the ambient air, resulting in either inhalation uptake or further dispersal [200]. Due to the high surface activity of QACs, inhaled dust particles can attack the surfactant of the lungs, which may cause and/or promote the development of COPD. In addition, QACs are highly cytotoxic to the upper respiratory tract. In patients with bronchial asthma, BAC, the main form of QAC, can induce bronchoconstriction at the 600 µg dose level [201]. Animal studies point to additional risks associated with exposure to BAC. BAC is classified as class I acute inhalation toxicity agent based on findings from animal studies. A 2-week exposure to a BAC-triethylene-glycol mixture induced severe respiratory symptoms and degenerative changes in the nasal cavity of rats [202]. When used to disinfect surfaces in animal husbandry (laboratory mice), reduced fertility, delayed embryonic development and impaired immune responses were reported as side effects [203]. Based on current understanding, a developmental neurotoxic effect of BAC also cannot be ruled out, although the oral intake of 120 mg BAC/kg body mass/d tested was relatively high. In pregnant laboratory mice, oral administration of BAC was shown to cross the blood-plasma barrier, reach the neonatal brain and alter brain sterol and lipid homeostasis [204]. BAC administered through the maternal diet during pregnancy passes the placental barrier and alters brain cholesterol and lipid homeostasis in newborn mice [204]. In 43 randomly selected participants (population sample), the presence of QACs in the blood was detected in 80%, and in half of these individuals, QAC levels varied between 10 and 150 nM (nanomolarity), which is the order of magnitude that affects cell metabolism in cell culture. Participants were found to exhibit a dose-dependent decrease in mitochondrial function and an increase in inflammatory cytokines. The concentration of cholesterol-synthesis pathway intermediates generally increased [205]. QACs have also been detected in breast milk at concentrations ranging from 0.33–7.4 ng/ml [206]. Since QACs are not only used in disinfectants and cleaning agents, but also as preservatives in personal care products and food processing, their origins cannot be established with any certainty. Given that surface disinfection in particular leads to chronic exposure by inhalation, these findings are alarming.

Allergic contact dermatitis caused by QACs and cross-reactivity with different QACs [207] has been known for some time [208], [209], [210]. In pig farmers [211], the use of disinfectants containing QACs was associated with asthma. The use of QACs for disinfection was also significantly associated with asthma in clinical nurses, as determined by questionnaires, workplace analysis, lung function testing and a specific IgE assay [212]. However, a questionnaire survey on the use of QACs in MD processing did not confirm an association between QAC use and asthma [213], presumably because of the lower level of exposure. A prospective cohort study (n=116,429; 14 US states) demonstrated an association between exposure to disinfectants and COPD in nurses irrespective of smoking and asthma status [214]. The use of hypochlorite and glutaraldehyde-based disinfectants was associated with an increased risk of asthma [215]. Prolonged exposure to a BAC-containing cleaning solution in the workplace resulted in occupational asthma. Changing the workplace resulted in complete remission [216]. The use of disinfectant surface cleaners has also been associated with bronchial hyperresponsiveness [215]. In any case, spray application of QACs should be avoided because of the high inhalation exposure [217].

**Phenol derivatives:** Today, these are rarely used in surface disinfectants, primarily for toxicological reasons. In addition, they have a comparatively low efficacy, a narrow activity spectrum, are difficult to degrade and produce AOX in the case of chlorinated cresols and phenols. In addition, they have an unpleasant, persistent odor [218].

**Glucoprotamine:** No data have been published on incompatibilities for glucoprotamine used as a surface disinfectant [219]. The application of glucoprotamine is associated with a low build-up on surfaces.

### 5.6 Waste disposal

Cleaning and disinfection agents whose ingredients are easily and completely biodegraded or at least eliminated during wastewater treatment, so that they cannot enter the water cycle, are preferable [220], [221]. There is a risk of developing resistance due to dilution effects depending on the active agent, an issue that has received little attention to date. When preparing the working solutions of cleaning agents and disinfectants, it is important to ensure that the quantity prepared is almost completely consumed, in order to minimise the wastewater load [221]. Concentrates, e.g., containers that are past their use-by date, must be disposed of as hazardous waste [222]; see the manufacturers’ safety data sheets and operating instructions.

### 5.7 Staff protection

When handling either concentrates or working solutions of surface disinfectants and cleaners, the risk to skin and mucous membranes from contact and inhalation of vapors must be considered. Protective measures specified by the manufacturer in the product-specific safety data sheet should therefore be carefully observed, for example:

- Prepare the disinfectant solution while wearing protective goggles, if necessary also mouth-nose protection,
- Wear suitable protective gloves where contact with the disinfectant and the solution for use may occur (this also applies to soaked cloths),
- Follow the order of mixing,
- Ventilate rooms during large-scale disinfection; respiratory protection masks may be necessary during disinfection measures requiring high ambient-air concentrations of irritating substances,
- When working in wet conditions or wearing waterproof gloves for >2 hours, implement a skin protection plan [149], [223].

Spray disinfection is only permitted in justified exceptional cases [217], e.g., if the surface cannot be reached by wipe disinfection [182].

If surface disinfectants or cleaners are advertised as spray products by the manufacturer, special instructions in the safety data sheet may apply. If surface disinfectants or cleaners are to be used for spraying but this is not specified by the manufacturer, respiratory protection may be required.

### 5.8 Fire protection

For alcohol-based surface disinfectants, additional protective measures are required due to fire and explosion risks. For example, the use of alcohol-based disinfectants is not permitted near open flames or other ignition sources. In accordance with TRGS 525 [182], the following apply:

- The total quantity applied per room must not exceed 50 ml/m^2^ room floor area for safety, health and explosion protection reasons.
- Aerosol formation must be avoided as far as possible. Hot surfaces must be cooled before disinfection.
- Disinfection may only be commenced when there are no other flammable gases or vapors in the ambient air.

It makes sense to summarise the work area- and substance group or substance-related operating instructions with the specifications from the hygiene, cleaning and disinfection plan as well as the skin protection plan in the same work instructions [182].

### 5.9 Conclusions

The following criteria apply to the selection of disinfectants:

- required activity spectrum,
- feasible contact time,
- risks to humans and the environment,
- compatibility with materials,
- acceptance (no or low odor nuisance),
- storage stability of working solutions and
- cost-effectiveness.

A minimization principle applies to the handling of hazardous substances, which states that the use of disinfectants must always be objectively justified and minimised as far as possible [154], [155].

The selection of disinfectant must be based on a careful risk-benefit analysis (see section 5.1 of the current recommendation and section 3 of the informative appendix, Attachment 1). In other words, the product with the broadest spectrum of activity should not be used in every situation without considering the associated risks. It is therefore preferable to select a product or product concentration that is sufficiently effective for the intended application.

For small surfaces, alcohol-based surface disinfectants are preferable due to their rapid action, provided that there are no material incompatibilities, because they are not only harmless to human health and the environment but are also biodegradable.

For toxicological reasons, formaldehyde-based surface disinfectants are no longer recommended for prophylactic disinfection and terminal disinfection. If disinfectants containing formaldehyde are to be used in disinfection measures ordered by the authorities, occupational safety measures must be performed to exclude any risks. Glutaraldehyde and glyoxal-based surface disinfectants should only be used if there is no available alternative.

For disinfection of sanitary areas (washbasins, toilets, shower trays, drains), peroxides with sporicidal activity are the agent of choice because they leave no residues, do not form AOX as chlorine-releasing compounds do, and are advantageous in sanitary areas [52]. Cleaning or disinfection products for sanitary surfaces often include acidic formulations to dissolve limescale, e.g., by adding citric, sulfamic or lactic acid, which all have relatively good material compatibility.

CDIs require the use of sporicidal surface disinfectants. Although sodium hypochlorite (bleach) is predominantly used in the USA and the UK whenever sporicidal activity is required, peroxides or PAA are recommended in Europe to prevent contaminating wastewater with AOX.

In case of viral infections, products declared as limited virucidal, limited virucidal PLUS or virucidal must be selected depending on the pathogen.

For open tuberculosis caused by *M. tuberculosis*, tuberculocidal products suffice. For non-tuberculous mycobacterioses, products with mycobactericidal activity should be used, e.g., peroxides. If an anti-mould activity is required, e.g., after remediation of moisture damage in accordance with the Federal Environment Agency Mould Guide [224], products declared as fungicides (efficacy is based on fungal spore testing, as these provide the only quantifiable measurements [72]) should be used. Since efficacy testing for dermatophytes is not included in the VAH test hierarchy [61], [62], if disinfection is necessary, for instance, in the case of particularly contagious dermatomycoses (e.g., tinea capitis caused by *Microsporum audouinii*), the best option is to ask the manufacturer for any available test reports. Alternatively, data on individual active ingredients [225] can also be obtained, although efficacy is influenced by the overall formulation.

Other requirements may therefore also need to be considered. For example, the specifications of food law must be followed in hospital kitchens. The DVG also publishes the DVG disinfectant list for the food sector, which specifically covers the application of chemical disinfection procedures in the food sector [74].

## 6 Procedures for surface cleaning, disinfecting surface cleaning and surface disinfection

The cleaning and disinfection of surfaces is mainly performed manually. Given that the resulting quality of manual wipe disinfection hinges on the human factor, non-contact equipment technologies have also emerged as alternatives in recent years (see section 6.2). Only time will tell if such techniques can replace manual wipe disinfection as part of standard precautions.

Probiotic detergents, i.e., probiotics combined with surfactants, have been developed as a promising option to reduce exposure to chemical substances (see section 6.3).

### 6.1 Methods using chemical disinfectants

There are a number of different methods for preparing chemical disinfectant solutions, their application and distribution on the given surface, and auxiliary materials used. The different conditions of use have yet to be compared in clinical trials. Part of the procedural efficacy testing involves simulating the application methods in practice-based trials. When selecting procedures, the efficacy, feasibility, effectiveness, health hazards and costs need to be evaluated.

#### 6.1.1 Wipe disinfection

Different methods are available depending on the nature and size of the surface to be treated and the indication for disinfection.

Surface disinfectants are either supplied as directly applicable ready-to-use solutions or as concentrates which need to be diluted on site to the appropriate working solution. Concentrates provide the advantage of lower transport and storage costs, but the risk of incorrectly prepared working solution concentrations is inherent.

The following should be noted:

- **Preparation of the working solution: **
  ***Important information:*** Working solutions should not be left uncovered to avoid altering their concentration and avoid any unnecessary pollution of ambient air; they should be used within one working day. If the manufacturer specifies a shelf life for the working solution, this should be respected.
- **Reusable or disposable textiles: **
  Disinfectants may be applied and dispersed with reusable textiles that can be processed or with disposable textiles. One study, which found that the life cycle assessment of a disposable wipe or mop head was more favorable compared to the reusable alternative, needs to be reviewed because the methodology described does not provide sufficient information to reach a conclusive assessment [226]. Although the life-cycle assessment of surgical gowns showed that reusable textiles were superior to disposable textiles [227], this conclusion is not transferable to wiping textiles. Overall, there are insufficient data available to draw any conclusions on wiping textiles. In order to select the more environmentally friendly alternatives, independent comparative life-cycle assessment studies on disposable and reusable wiping textiles (including wipe dispenser systems versus ready-to-use wipes) are needed.

**Reusable textiles: *Important information*:** In principle, it should be noted whether the manufacturer of the processable wiping textile provides information on the maximum number of processing cycles, as material wear increases with each use and each processing cycle. Excessive processing may result in the loss of cleaning characteristics [228]. Accordingly, reusable textiles should be disposed of at the latest when they no longer fulfil their intended purpose. Reusable textiles need to be processed using validated chemo-thermal or thermal disinfection washing procedures, unless pre-soaked mop heads are used. They are subsequently dried and must be stored in a hygienic manner, specifically in a dry place, to prevent the propagation of microorganisms [229], [230]. All process parameters, such as concentration of the process chemicals, contact time, temperature and diluting effects, need to be strictly observed. Other additives, such as alkaline wash enhancers, can only be added if the efficacy of the process with this additive has been confirmed under specified conditions. Microbiological testing of processed, reusable textiles revealed clear deficiencies when the above requirements were not met [231]. In addition, when using processable textiles with different material qualities, there is a risk of the active ingredients of the disinfectant being adsorbed onto the material. If the cloth has not been tested with the specific disinfectant, a degree of uncertainty remains regarding its effectiveness, as the level of liquid absorption and active ingredient adsorption varies depending on the material. Wipe textiles used should offer a high absorption capacity for liquids, be resistant to cleaning detergents and disinfectants, have low fluffing characteristics and be easy to process at high temperatures.

**Disposable textiles:** Two options are available: ready-to-use wipes soaked with disinfectant solution in disposable packaging (e.g., as commercially available flow packs or compact free-standing containers) and pre-soaked wipe dispenser systems. The manufacturer is responsible for ensuring that the textile and disinfectant are compatible and that the efficacy of the disinfectant is consistent over the application period. 

***Important information:*** The advantage of ready-to-use products is their immediate availability at their place of use. They are available in different sizes for all spectra of action. The shelf life after opening is up to 3 months. Disadvantages are the high volume of waste generated, which includes plastic packaging, the potential that insufficient liquid is delivered to the surface to be disinfected, the risk of a limited shelf life, and high costs.

The requirements that apply to pre-soaked wipe dispenser systems are even more stringent for ready-to-use soaked disposable wipes.

- **On-site preparation:**
  Disinfection may be carried out either with dry wiping textiles that are soaked in a freshly prepared disinfectant solution (working solution), pre-soaked textiles, or with cloths that have been packaged ready-for-use. The manual pre-soaking method requires a daily supply of ready-to-use, processable wipe dispenser systems or wipe dispenser systems containing dry, non-woven wipe rolls, that are then processed by filling with the disinfectant solution for pre-soaking. The shelf life of manually disinfectant pre-soaked cloths depends on the system. Pre-soaked reusable textiles usually have a shelf life of one working day if stored properly (continuous storage of the wiping textile in the working solution in a suitable closed container). For wipe dispenser systems, it is generally 28 days. The manufacturer of the wipe roll or the disinfectant used in pre-soaked wipe dispenser systems must provide expert evidence, e.g., by chemical analysis, that active-ingredient activity is not lost during the specified shelf life [232]. Before deploying wipe dispenser systems, the suitability of the system for the application area must be evaluated.
  Mechanical pre-soaking (processing) is carried out in washing machines designed for this purpose; manufacturer’s instructions for the cleaning textiles, washing machines, dosing devices and cleaning and surface disinfectants used must be followed [148]. Wiping textiles are processed in the washing machine. This involves pre-soaking the textiles with disinfectant in the washing machine and then storing in a closed container until wiping textiles are removed for use (note: comply with the specified maximum storage times). The procedure for machine pre-soaking must be validated in accordance with DIN 13063 [148]. The intervals for revalidation are to be determined with the auditor in collaboration with hospital hygiene. Due to the variety of influencing factors (e.g., dilution effects, detergent residues), the process must be viewed critically [233], [234]. DIN 13063 specifies a number of criteria for determining whether the machine-pre-soaked wiping textiles produce the desired disinfection result right up until the end of the shelf life [148].
  ***Important information*:** In the case of wipe dispenser systems, if the shelf life is surpassed, the dosage is too low and/or the containers are inadequately processed, this may cause any introduced infectious agents to become tolerant to disinfectants and create a potential infectious reservoir, potentially with the formation of biofilms. Previous studies detected contaminations particularly with formulations based on surface-active substances, more rarely also with aldehyde-based formulations, but so far not with alcohol-based active substances [235], [236], [237]. Therefore, the VAH disinfectants commission does not recommend the use of processable wipe dispenser systems – except systems with alcohol-based disinfectants – in areas at increased risk of infection, e.g., intensive care unit, hematology/oncology, neonatology, burns ward. This recommendation also applies to systems where only the closing mechanisms (lids) are processed [238].
  Before reloading wipe dispenser systems, the container and lid must be processed with a bactericidal, fungicidal and sporicidal activity spectrum, according to the manufacturer’s instructions. Processing of an experimentally contaminated wipe dispenser system in the washer-disinfector (WD) machine, regardless of whether chemical cleaners were added or not, was shown to prevent recontamination, provided a temperature of 60–70°C was maintained for at least 5 min. Similarly, pre-cleaning with hot water or a thorough cleaning step followed by wipe disinfection using a disposable wipe and oxygen-releasing disinfectant prevented recontamination of the disinfectant. However, the efficacy of neither processing method was tested for contamination with spores [239].
  Independent of verifying that the wipe dispenser systems are handled correctly, in the context of outbreak situations with relevant pathogens and particularly with Gram-negative bacteria, a hygiene-microbiological inspection of the processable wipe dispenser systems should be carried out, [232]. In addition, an annual, random hygiene-microbiological inspection by membrane filtration may be considered to monitor the processing. The recommended limit for disinfectant solutions in processable wipe dispenser systems is 0 CFU/10 ml for Gram-negative microorganisms and 3 CFU/10 ml for aerobic spore-forming organisms and other apathogenic environmental flora (e.g., *Micrococcus* spp.). Testing should include all processable wipe dispenser systems in use. Processable wipe dispenser systems in non-risk areas can be reviewed for initial indications on suitable culture media for testing (e.g., dip slides) [240].
  To ensure that staff are protected and to prevent contamination, the cloths need to be removed with fresh or disinfected protective gloves of the appropriate resistance class.
  In practice, observations have shown that if the lid is not closed, disinfectant wipes protruding from the bucket dry out and lose their efficacy. There is also a risk of contamination with Gram-negative bacteria, e.g., as a result of coming into contact with contaminated gloves, particularly from wet areas. It is necessary to ensure that disinfectant wipes do not dry out, e.g., as a result of incorrect closure of the system, and that wipes do not protrude freely. In addition, the working instructions should state that the lids of processable disinfectant wipe dispenser systems should be securely closed when the wipes are not being used [232]. The quality of the textile needs to enable optimal soaking to the last wipe, i.e., the liquid content needs to remain constant and there must be no evaporation.
- **Application procedure:**
  The application of the surface disinfectant should be as standardised as possible.
  ***Important information*:** To ensure the effectiveness of the disinfection, the surface must be completely wetted with an appropriate amount of disinfectant solution (individual visual control of the wetting may be useful). It should be noted that manufacturers do not generally provide information on the range of appropriate wetting for any of the wiping textiles. Although a suitable method has been proposed for ready-to-use wipes [241], ultimately it is hoped that this information (m^2^ surface that can be disinfected/wipe) will be available to help in practice. As long as the efficacy of ready-to-use disposable wiping textiles is certified in the 4-Field Test, the quantity required as a function of surface area is not necessary.
  The applied disinfectant should not be removed by wiping with water or dry wiping before completion of the contact time. A notable exception is the kitchen area, where disinfectant residues are removed, e.g., by rinsing, after completion of the contact time.
  In general, disinfection procedures need to uphold the discard principle, i.e., the wiping textile should only be immersed in the disinfectant solution once, because the efficacy of the disinfectant is reduced or eliminated by contaminants that are transferred into the solution. Re-immersion of the wipe is not allowed. To implement this method, several wipe textiles should initially be immersed in the clean working solution before use, then continuously removed as needed during the cleaning or disinfection process and finally directly collected for processing or disposal (this is called the mop-head changing method).
  If the surface is heavily contaminated, e.g., with blood, vomit, etc., the contaminant needs to be removed immediately by mechanical means (do not use disinfectants because they fix protein). Disinfecting surface cleaning or surface disinfection is only carried out thereafter (two-step procedure).
  To prevent the spread of pathogens, differently colored textiles should be provided for cleaning and disinfecting surface cleaning in different areas. The cloths must be processed or discarded after each patient unit (patient room incl. sanitary area) [148], [180], [242]. The different colors also allow the patient to see whether near-patient hygiene measures are being carried out correctly. For disinfecting surface cleaning of floors in areas with an increased risk of infection (see Table 2 (Tab. 2)), it has proven to be sensible to start in the unclean area in order to prevent spreading pathogens and to avoid re-contaminating clean areas via shoes. The protective gloves of the housekeeping staff (see section 5.7) must be disinfected or changed after each patient unit [40].
- **Processing of used auxiliary materials: **
  After use in areas where there is no increased or potential risk of infection (see Table 2 (Tab. 2)), it suffices to clean potentially contaminated regions of the cleaning trolley (e.g., handles, containers, rollers, holders) at the end of the last shift, but at least once every day of use, and allow them to dry [148].
  ***Important information*:** After use in areas where there is an increased risk of infection, or in any potentially contaminated areas on the wash trolley (e.g., handles, containers, rollers, brackets), these must be cleaned with disinfectant at the end of the last shift, but at least once every day of use, and allowed to dry. For the processing of wiping textiles, see section 7.3.2.

Table 5 (Tab. 5) summarises the advantages and disadvantages of the different floor disinfection options.

#### 6.1.2 Spraying method

With spray disinfection, the disinfectant is sprayed onto the surface to be treated. This results in inhalation exposure of staff, which is why this procedure is not recommended for occupational safety reasons [183], [185] or is only deemed permissible in justified exceptions [182], if the area cannot be reached by wiping but requires disinfection [217], [243]. If the disinfectant is applied as a foam instead of a spray, inhalation exposure may be disregarded.

Table 6 (Tab. 6) summarises the advantages and disadvantages of the different options for disinfecting small surfaces.

#### 6.1.3 Surface cleaning by machines

Scrubbing machines can be used for synthetic coverings and tiles, and spray extraction methods can be used for textile floors [244], [245]; however, there are currently no test methods to prove the cleaning or disinfecting efficacy of the methods using machines [148]; therefore, machine methods for surface cleaning should only be used in areas without an infection risk.

The use of scrubbing machines for cleaning floors in health-care facilities does not lead to increased microbial ambient air pollution when machines are operated and maintained properly. In the absence of available data on the efficacy of disinfecting surface cleaning, these machines should only be used for cleaning purposes [246].

This application requires that the machines be operated according to the manufacturer’s instructions with respect to maintenance and processing (preference should be given to hoses, suction lips and brushes that can be processed mechanically/[chemo]thermally) [148]. The mentioned requirements and the processing frequency need to be substantiated by the appliance manufacturers by means of expert opinions.

### 6.2 Non-contact equipment-based procedures

Non-contact equipment-based disinfection processes include chemical and physical principles of action. As these have no cleaning effects, additional cleaning is required to remove any contamination before using these procedures.

Non-contact technologies including nebulization of H_2_O_2_ are not considered in the context of standard precautions; they may be used as a supplement in the context of terminal disinfection or outbreak control, if necessary.

To date, non-contact physical equipment-based procedures should not be used in areas with an increased or specific risk of infection (see Table 2 (Tab. 2)), due to the lack of test standards for the disinfecting effect and the resulting uncertainty in terms of efficacy.

#### 6.2.1 Chemical procedures

**Aerosols and nebulization:** Different disinfectants are used in disinfection processes that apply the disinfecting agent as an aerosol or nebulization.

Room disinfection by vaporising or nebulizing formaldehyde is only required in extremely rare, exceptional cases [247]. Should this procedure nevertheless be required in specific situations as part of an officially ordered disinfection measure, the detailed conditions of use are given in the list of disinfectants and disinfection procedures tested and approved by the RKI [76]. In addition, TRGS 522 [248] applies to the application of the procedure.

For room disinfection, the nebulization of H_2_O_2_ is increasingly being discussed as a supplement to wipe disinfection [249], [250], [251], [252]. An essential condition for use is the manufacturer’s evidence of efficacy [253]. Since the efficacy of the method is highly dependent on the furnishings of the room (e.g., existing fixtures, textiles), the size of the room and the accessibility by the method, it is important to evaluate the efficacy in a practical test to ensure an effective application. If necessary, this may be based on results from similar rooms. Information on the methodological procedure for this is given in the list of disinfectants and disinfection procedures tested and approved by the RKI [76]. When used accordingly, a significant reduction of the pathogen load on the treated surfaces can be demonstrated [252], [254], [255], [256]. A reduction in the incidence of nosocomial colonisations and infections [249], [251], [254], [255], [257] as well as efficacy in controlling outbreaks [254], [258], [259], [260], [261] have also been demonstrated. Nebulization with H_2_O_2_ may therefore be considered in the event of an uncontrollable outbreak [260]. Nebulization of rooms after occupancy by COVID-19 patients and before new admissions has also proven to be feasible. Overall, the evidence to date is insufficient to support the deployment of such procedures [249], [250], [251], [252], and the limitations are numerous [76], [183], [184], [247], [251], [252], [262], [263]. Consequently, at this point in time, no recommendation can be given for the use of H_2_O_2_ nebulization as terminal disinfection.

**The following is required for effective disinfection:**

- H_2_O_2_ is only effective in direct contact with the surface. To cover the largest possible surface area, open cupboards and drawers and remove other materials in the room that cover potentially contaminated surfaces beforehand and then disinfect them separately. Devices and openings that make it difficult for the disinfecting agent to penetrate due to their design, as well as ventilation systems, must be given special attention [264].
- Effective disinfection depends on the dimensions of the surfaces in the room that need to be disinfected and the ambient air flow behaviour (e.g., switching off the ventilation and air conditioning [A/C] system, taping off ventilation shafts). This is why the reproducible efficacy of nebulization procedures and the application of aerosols must be tested in the context of on-site validation [76], [264], which is not described in the studies cited. For effective use of the processes, technical solutions such as sensors are required to provide a continuous measurement of the H_2_O_2_ concentration in the ambient air during nebulization for the duration of the previously determined contact time to ensure disinfection efficacy.
- Furthermore, the efficacy is highly dependent on the nature of the surfaces to be disinfected. For example, the time required to appropriately reduce the level of pathogen solely based on its adherence to another surface may increase the contact time tenfold [265], [266].
- It should be noted that efficacy is not achieved for blood residues and that it has not been specified for fluid accumulations, for instance. Nebulization therefore only makes sense as a supplement to the previously performed wiping method of surface disinfection.

**The following is required for compatibility:**

- Room disinfection by H_2_O_2_ nebulization only applies to unoccupied rooms, i.e., as part of terminal disinfection, because inhaled H_2_O_2_ is highly toxic at the 10% application concentration and is irritating to the respiratory tract at much lower concentrations (irritation threshold 10 mg/m^3^) [267]. Consequently, the rooms can only be occupied again after longer ventilation intervals [76], [262], [263], [268], [269]. Patients may only access the room when the H_2_O_2_ concentration has fallen below the derived no-effect level (DNEL) for acute inhalation exposure [194]. Ideally, the drop in H_2_O_2_ concentration after nebulization is tracked and documented by the H_2_O_2_ disinfection device itself, by continuous measurement of the ambient air. For working rooms, activities may already be started when the OEL is below 0.5 ppm. Efficacy can only be guaranteed by accompanying measurements of the disinfection device, as the time required for ventilation until H_2_O_2_ decays depends on the room situation. The room is automatically given clearance by the disinfection device to ensure the safety of patients and staff. Should complications arise during the disinfection process (e.g., if the A/C system is not switched off), the process needs to be stopped automatically by the H_2_O_2_ disinfection device and the operating personnel notified. Procedures involving nebulization solutions containing >8–35% H_2_O_2_ require additional precautions due to their hazard potential [270].
- To insure the safety of staff and patients, the room that is nebulised with H_2_O_2_ should be separated from the surrounding area. Tape off all open connections to other areas, e.g., door gaps, ventilation shafts, heating pipes and sockets. The manufacturer needs to provide evidence that the material used is suitable for this purpose.
- To demonstrate the successful progress of the disinfection process, a final report including room data, the amount of H_2_O_2_ consumed and the clearance measurement of the room from the device should be generated.
- To reliably ensure disinfection, a programme for annual calibration and quality assurance of the nebulization device should be implemented. In addition, the manufacturer should provide instruction when the device is first used.
- It is also important to note that the process may cause material damage, and that the manufacturer’s instructions generally do not extend to the MDs that remain in the room. The operator of the MD is therefore responsible for ensuring that the processed MD is fully functional for its intended purpose and that it is safe to use, even if it has been processed differently from the manufacturer’s instructions.

Given that ozone is highly toxic, genotoxic and suspected of being carcinogenic [160], its use in health care facilities is not relevant for surface disinfection. However, the uses of ozone for disinfection in the health care sector continue to be evaluated [271], [272], [273], [274], [275], [276] [277], [278].

The “Chemical Risks” working group of the Health Care Section of the ISSA, in collaboration with the Employer’s Liability Insurance Association for Health Services and Social Services (Berufsgenossenschaft für Gesundheitsdienst und Wohlfahrtspflege), has analysed the hazards and protective measures for activities involving disinfectants in the health care sector. Its Factsheet 8 from 2014 notes that fumigation procedures possess a high risk potential for the operators and the environment, and that high demands are therefore placed not only on the competence of the persons carrying out the procedure but also on the sequence of the individual steps of room disinfection, i.e., preparation, implementation and follow-up [183], [184].

#### 6.2.2 Physical procedures

**Steam:** Steam (e.g., steam cleaners) is a resource-intensive as well as unreliable method for reducing pathogen loads. The following problems still remain unresolved: lack of validation of the efficacy and cleaning effect in addition to the risk of pathogen spread. Under specific conditions, steam reduced the pathogen load on surfaces [279], but without cleaning. A mobile system only reduced coliforms by <1 log_10_ [280]. In contrast, an ICU application study showed that steam disinfection was equivalent to a two-step procedure consisting of cleaning and subsequent application of 1,000 or 5,000 ppm hypochlorite in terms of MRSA, VRE, carbapenem-resistant *P. aeruginosa* and multidrug-resistant *A. baumannii* [281].

**UV and HINS irradiation:** UV and high-intensity narrow-spectrum (HINS) irradiation (405 nanometre [nm] HINS irradiation) can have a bactericidal, sporicidal and virucidal efficacy depending on the dose of irradiation [282], [283], [284]. Because of the potential shadowing, pathogens are not reliably killed on highly structured surfaces (e.g., pores, depressions, caverns, inner lumina) and surfaces with a flat radiation angle. This essentially limits the application of UV and HINS irradiation to smooth surfaces. Devices need to be operated and maintained according to the manufacturer’s instructions (in particular, the number of hours the lamps are left on need to be monitored to guard against undue energy losses). The safety of users with regard to radiation exposure and potential exposure to ozone must be ensured at all times. In addition, material compatibility with the process must be assessed.

Antimicrobial irradiation of surfaces by UV or HINS irradiation using mobile irradiation devices (robots) has been increasingly used as part of terminal disinfection after discharge of patients infected with *C. difficile* or MDRO in more recent years [255], [285], [286], [287], [288], [289], [290], [291], [292], [293], [294], [295], [296]. Most studies indicate that these types of procedures are effective in preventing nosocomial infections caused by *C. difficile* and MDRO. A conclusive estimate of UV or HINS irradiation efficacy for preventing defined nosocomial infections is not yet possible [15], [294], as the studies performed to date do not address whether the technology is superior or inferior to current wipe-disinfection practices.

### 6.3 Probiotic cleaning methods

Microbiome studies have shown that a reduced diversity of microorganisms can lead to an increased risk of infection. It has therefore been postulated that it is not only the presence of pathogens, but also the absence of a diverse composition of non-pathogenic environmental bacteria in the hospital environment that may contribute to the development of NIs [297], [298], [299]. This rationale led further studies to investigate the influence of probiotic microorganisms on the hospital microbiome and its impact on NIs.

Probiotic detergents deploy different bacterial species (e.g., *Bacillus* spp., *Lactobacillus* spp., *Streptococcus* spp.). Colonisation with probiotic bacteria can prevent the propagation of pathogens by increasing competition for nutrients and space and secreting secondary metabolites that provide a survival advantage [300]. According to the competitive exclusion principle, also known as Gause’s principle, Gause’s law, Gause’s hypothesis, the Volterra-Gause principle, Grinnell’s axiom or the Volterra-Lotka equations [301], species competing for the same limited resource cannot coexist in a homogeneous habitat. If microorganisms are removed from a surface by disinfection, diversity is disturbed, allowing a pathogen to thrive and colonise the space because of reduced competition [302].

The use of probiotic cleaning methods in experimental settings have been shown to reduce the surface pathogen load by as much as 90% over conventional wipe disinfection [303], [304] without selecting for MDRO [305], [306]. In a multicentre study, the probiotic procedure reduced the cumulative incidence of NIs from 4.8 to 2.3% (p<0.0001) when compared to chemical disinfection. In addition, the detection of antimicrobial resistance genes decreased by up to 99%, antibiotic use to combat NIs decreased by 60.3% and resulted in a 75.4% decrease in associated costs [305], [307]. However, the before-and-after design of this multicentre study represents a potential bias. The result was nevertheless almost completely confirmed in a subsequent study, which reported a reduction in the cumulative incidence of NIs from 4.6 to 2.6%, a decrease in severe NIs from 1.57 to 1% and MDR infections from 1.13 to 0.53% [308]. In addition, a comparison of cleaning with soap, disinfection and probiotic procedures found that using soap and probiotic bacteria stabilised the surface microbiome by displacing *Escherichia (E.) coli* and *S. aureus* when compared to disinfection [309]. So far, there is no evidence that probiotic bacteria put patients at risk [310]. Since viruses do not propagate outside of their host cells, it cannot be assumed that they are displaced by the microbiome when surfaces are contaminated with probiotic bacteria. Consequently, chemical disinfection is indispensable when virus disinfection is indicated. Overall, the use of probiotics on surfaces in medical facilities is an interesting approach because instead of nosocomial pathogens, probiotic bacteria form a long-lasting stable microbiome [311], [312], in contrast to disinfection, which only lasts for a short time [313], may result in the development of resistance involving cross-resistance to antibiotics depending on the active ingredient being used, and may pose a risk to humans and the environment. A prerequisite for the safe application of probiotics is a guarantee of microbiological product quality, because evidence exists of extraneous contamination occurring [314]. The effectiveness of probiotic cleaning procedures needs to be studied more thoroughly before deriving any general recommendations for their use in hospital settings [315].

## 7 Building and equipment requirements

### 7.1 Rooms and furnishings

Suitable premises are a prerequisite to ensure a consistently high quality of surface disinfection and disinfectant surface cleaning. The following findings are based on an inpatient setting and are to be considered for outpatient settings as far as risk and room-specific functions are concerned. Storage rooms must be available, e.g., for tools and cleaning trolleys (cleaning-equipment rooms). If cleaning-agent rooms do not have windows, they must have an air-exhaust system.

Depending on the building configuration, rooms may be centralised or decentralised. In the case of a centralised arrangement, staff must have access to disinfection aids outside of main operating hours. Alternatively, ready-to-use disposable wiping textiles soaked in disinfectant solution can be kept on hand. DIN 13063 [148] can be used to provide further guidance on the requirements for central premises, e.g., power connections, drains, changing facilities.

For setting up and dismantling the cleaning trolleys, an appropriately large room should be available to allow separation into unclean/clean areas with central or decentralised aeration and air exhaust.

Appropriately sized, ventilated rooms with equipment for processing, including separate drying facilities (if this is not already done during the processing), are needed for the processing of used auxiliary materials. The processing procedure needs to be organised in such a way that unclean and clean areas are separated from each other, e.g., optimally by means of a tunnel washer. The processed auxiliary materials must be stored in such a way that they are protected from contamination (separation of clean/unclean areas). In an analysis of 44 planned hospitals, this separation was lacking in 42% of cases. Inadequate ventilation was noted in 12% [316].

### 7.2 Requirements for surfaces in medical facilities with regard to cleaning and disinfection

Functionality, safety and hygiene must be given equal consideration right from the planning phase, i.e., cleaning and disinfection should be designed to be both efficient and very user-friendly. Structurally, poorly accessible niches should be avoided.

Floors and other hygienically relevant surfaces should be easy to clean and disinfect, i.e., surfaces should be even, easy to wipe and seamless wherever possible. Carpets are not suitable for therapy and care areas.

Instead of conduits, which can often suffer damage and can be costly to repair, appropriately grouted plinths are easier to repair and can be easily reached with wiping textiles.

Surface wettability affects how easily a surface may be contaminated and influences the result of surface cleaning and disinfection. Low wettability (e.g., lotus effect) can adversely impact the result of the disinfection. In other areas where hygiene is paramount, such as the food industry, a surface roughness (Ra) of 0.8 micrometres (µm) has proven to be suitable for mechanically treated surfaces [317]. Open-pored and granular materials should be avoided.

Materials or coatings should be selected that possess long-term resistance to the expected physical (e.g., UV radiation, temperature exposure) and chemical effects (e.g., cleaning and disinfecting agents) as well as other influences. In the real clinical setting, many intensively used surfaces in patient care are defective after a few years and reveal areas that cannot be safely disinfected and where dirt accumulates. Especially in high-risk areas, steps should be taken to ensure that surfaces can be disinfected.

To reduce inaccessible surfaces, cupboards should extend to the ceiling and be flush with the floor. Radiators should be designed to prevent creating inaccessible spaces.

### 7.3 Equipment requirements

#### 7.3.1 Disinfectant dosing devices

Effective disinfection requires accurate dosing of the disinfectant to prevent selection of micro-organisms with increased disinfectant tolerance or resistance. Depending on the size of the facility, this is best achieved by automatic dosing in decentralised disinfectant dosing units [318]. Central disinfectant dosing units with a pipe network throughout the facility are no longer used because of their disadvantages (costs, no remediation feasible in case of biofilm formation). However, even with decentralised disinfectant dosing devices and especially with combination devices that are used to dose cleaning agents in addition to disinfectants, biofilm can form in the lines conveying the fluids.

Incorrect operation, which poses a risk of inadequate disinfection and/or the selection of disinfectant-tolerant microorganisms due to the use of sub-concentration levels of disinfectants, should be prevented by conducting technical inspections at least once a year [318]. In the case of (generally rare) nosocomial outbreaks, a hygiene-microbiological check may indicate the presence of biofilms.

#### 7.3.2 Processing of wiping textiles

For processing, there should be a separation from other laundry to be processed based on the degree of soiling, material condition (e.g., with the risk of introducing extraneous chemicals) and hygiene risk. Textiles used for cleaning may be processed in a household washing machine and dried in a household tumble dryer; air drying is not recommended because of its inherent risk of spreading pathogens and recontamination. If wiping textiles are used for disinfection, higher processing standards must be observed than if they are used for cleaning purposes. If processing is not feasible, disposable wiping textiles should be used.

Reusable wiping textiles used for disinfection must be processed in a thermal or chemo-thermal disinfection washing procedure with proven efficacy, e.g., following the specifications of the disinfectant dosing device list of the VAH, or according to the list of disinfectants and disinfection procedures tested and approved by the RKI, or with proven efficacy in a practical test (e.g., cotton cloth). Processing needs to ensure that dirt and organic contamination are removed and that pathogens are no longer detectable. In the case of wiping textiles used for disinfection in areas with an increased risk of infection, prevention of pathogen carry-over should be prevented during transport to processing (e.g., waterproof packaging). Washing is followed by machine drying. Unless pre-soaked wiping textiles are used, the completely dried wiping textiles should be stored in a dry place until further use to prevent the propagation of any potential residual flora. Processed wiping textiles must be protected from recontamination.

The requirements for the disinfection washing process are for instance specified in DIN 13063 [148]. If the machine is only equipped with a manual detergent dispenser, graduated dispensing accessories need to be used. Annual maintenance of the washing machines is recommended. Regular wash cycle inspections are to be carried out with bioindicators [319]; a statement on inspection intervals is not yet available [320], but an annual inspection appears sensible. In the event of an outbreak, a hygiene-microbiological examination of potential sources, e.g., wiping textiles used and processing procedure, is indicated.

For the outpatient sector, it is also possible to outsource processing to certified laundries (e.g., RAL-GZ 992/2 for “hospital laundry” [321]). If wiping textiles are processed in laundries that operate according to the RABC (risk analysis and biocontamination control) quality assurance system, revalidation of the washing process needs to be carried out annually or after any process changes according to DIN 14065 [322].

### 7.4 Compatibility of materials with cleaning and disinfection procedures

When disinfecting surfaces, a distinction must be made between surfaces of furniture and fixtures on the one hand and MDs on the other. All furniture and fixture surfaces that need cleaning, disinfecting surface cleaning or surface disinfection must be resistant to cleaning agents and disinfectants used for the intended purpose as well as to the mechanical cleaning system. This is why chemical resistance is an important selection criterion.

If furniture or fixtures used in the patient care setting are to be purchased, these need to be amenable to and able to withstand cleaning and disinfection. To this end, the manufacturer needs to provide valid information, e.g., proof of effectiveness for cleaning and, if applicable, disinfection procedures, material compatibility and, if applicable, the influence of disinfection on the service life of the product. Prior to procuring fixtures and flooring, it is advisable to request the manufacturer’s cleaning and disinfection specifications by means of a checklist to avoid having to use different groups of active substances for disinfection because the surfaces are different, which would lead to confusion in practice (example of a checklist in [323]).

If manufacturer’s instructions for cleaning and disinfection of the operating or device surfaces of the MD are given, these must be followed after a plausibility check. In addition, the manufacturer’s instructions for processing in accordance with the Medical Devices Operator Ordinance (MPBetreibV) [324] must be considered.

In the event of incomplete and/or implausible information on the cleaning and disinfection of operating or device surfaces, a request for completion, specification and/or correction needs to be submitted to the manufacturer. In practice, it is problematic if different agents need to be used for different devices. One possible solution to the problem is to choose one type of product for sensitive surfaces and one for non-sensitive surfaces, and to have the manufacturer of the device or disinfectant dosing device confirm in writing that it is approved for use [323].

Incompatibilities should be considered e.g., when using:

- QACs on certain rubber coverings,
- alcohols on acrylic glass, soft PVC and polystyrene,
- Glucoprotamine on silicone, if used continuously also on polycarbonate, polysulphone and acrylic glass,
- acidic products on cement-based materials (e.g., terrazzo floors) and tile joints,
- chlorine compounds on corrodible metals and even standard stainless steels,
- PAA on corrodible metals and
- alkaline products on aluminium and linoleum floors.

Tantalum, aluminium (99.6%), tin (99%), polished and cleaned stainless steels, perbunan, many plastics (PE, PVC, polytetrafluoroethylene), borosilicate glass, white stoneware and porcelain are inert to H_2_O_2_.

### 7.5 Anti-adhesive and antimicrobially effective surfaces

Anti-adhesive finishes (e.g., super hydrophobicity, zwitterions, hydrogels, nano-structuring) can minimise the adhesion of microorganisms through steric or electrostatic effects. For this reason, the introduction of antimicrobially impregnated or effective surfaces is increasingly being discussed in health-care facilities [325] as a means of reducing the pathogen load on surfaces between disinfecting surface cleanings. Whether these surfaces are suitable to complement standard precaution measures in terms of disinfecting surface cleaning needs further independent investigation. Antimicrobially finished surfaces are tested according to ISO 22196 [326]. However, the process parameters of the standard do not reflect the practical conditions of use (different temperature, humidity, contact time, pathogens [327], organic load [328]), and do not give any indication about the reliability of efficacy; thus, they must be verified in practice [329]. Copper surfaces have been shown to exhibit an antimicrobial efficacy *in vitro* and *in situ* [330], [331]. The results of 4 out of 638 selected studies showed that the rate of NI tended to decrease under the influence of copper surfaces; however, the quality of the studies in some cases was characterised by substantial methodological weaknesses and conflicts of interest, which means that no recommendation for use can be derived at present [332].

The widespread use of materials with added active substances at sub-bactericidal concentrations, such as triclosan, BAC, chlorhexidine, silver and copper, presents a risk of developing tolerance and resistance that correlates with antibiotic resistance [149], [162], [166], [324], [333], [334], [335], [336], [337], [338], [339]40] and the loss of biodiversity. The uncontrolled use of copper and silver-based products, for example, is therefore considered critical not only with regard to the finishing of surfaces, but also in animal feed and cosmetics [340], [341], [342].

It is generally important to bear in mind that antimicrobial surfaces are only a supplement to disinfectant surface cleaning or surface disinfection, and that trusting in their antimicrobial properties may lead to neglecting standard precaution measures.

## 8 Quality assurance

Surface cleaning, disinfecting surface cleaning and surface disinfection must be considered as processes for which standard operating procedures need to be developed. Proper implementation can be ensured through auditing systems [6].

The performance and frequency of surface cleaning and disinfecting surface cleaning must be specified in the cleaning and disinfection plan on a room- and surface basis for each medical facility; alternatively, in the case of external service providers (building cleaners), these must be included in the service description (service description in DIN 13063, Annex A [148]) along with instructions for performance (see also section 4.2). Where an external service provider is commissioned, this provider is responsible for the qualified implementation; otherwise, the head of the facility needs to ensure that the staff in charge are qualified. DIN 13063 lists detailed requirements for the service provider, such as responsibilities, authorities and qualifications [148].

The effectiveness of surface cleaning, disinfecting surface cleaning and surface disinfection depends not only on the choice of products but also on compliance with quality standards [6], [7], [147], [343], [344]. It has been demonstrated that less than half of the near-patient surfaces are regularly cleaned [125], [345], [346], [347]. After terminal disinfection, the target pathogen was still detected in up to 60% of cases [348].

Ensuring that both cleaning and disinfecting surface cleaning are carried out in accordance with quality standards is a source of ongoing discussion among hospital staff, patients and visitors [349]. The following information on quality assurance was provided by an online survey involving 10% of German hospitals [350]: The hospital hygiene department monitored the quality of cleaning by visual inspection in 51% of cases, by contact culture in 35% and by fluorescent marking in 12% of cases. The quality of cleaning was monitored monthly (28%), quarterly (24%), semi-annually (28%) and for special reasons (e.g., change of staff, change of cleaning system or outbreak) (20%). Patient rooms were not cleaned on Sundays in over 50% of reporting hospitals, and not on Saturdays and Wednesdays in 13% and 16% of hospitals, respectively.

Measures to improve the effectiveness of surface cleaning and disinfecting surface cleaning were often investigated in bundles. Bundles that included training and coaching, dedicated staff and monitoring using checklists resulted in improvements [4].

### 8.1 Requirements for staff, human and material resources

To ensure infection prevention, the necessary qualification and training of facility staff must be regulated in accordance with section 23 (3) IfSG, which also includes standard precaution measures. The head of the facility is responsible for implementation of surface hygiene measures which meet the quality requirements; external service providers or in-house staff may be called upon for this purpose.

The requirements for both surface cleaning and disinfecting surface cleaning need to be provided at all times to ensure that they are carried out correctly and effectively. For instance, the service provider is required to ensure that the staff, equipment and expertise necessary to carry out cleaning and disinfection work, including the ability to implement it, are in place. DIN 13063, for example, calls for introductory and training programmes, and Annex D lists the subjects to be covered by technical instruction which are suitable as aids to training, based on facility size [148]. The optimal solution is to assign specifically trained staff to risk areas. The service provider and the client shall agree on a contingency plan to cover sick leave and holidays.

Additional training is required before introducing new technologies or disinfectant dosing devices. Because the supervisor is responsible for the continuous improvement of the housekeeping staff’s performance of their duties, the staffing ratio of supervisor:housekeeping staff should be determined and specified in the contract, if outsourced to a service provider. The same applies to the definition of the number of cleaners.

In order to assess the quality of the measures performed and the resulting implementation for quality improvement, close cooperation between the entire specialist staff for hygiene and infection prevention (hospital hygienist, hygiene specialist and, if applicable, hygiene engineer; hereafter referred to as hygiene team) and the service provider is required. The joint development and risk-based definition of cleaning and disinfection measures are crucial to create a working atmosphere of mutual understanding and collegial cooperation.

In a before-and-after study in a paediatric ward for immunocomprimised patients, the use of more qualified surface cleaning staff reduced environmental contamination with noroviruses from 20% to 6% and with rotaviruses from 15% to 10% [351]. This example illustrates how important it is for surface cleaning to be carried out by professional staff.

Training can more than double the quality of results [352]. In line with expectations, the combination of monitoring and training also leads to a significant improvement the quality of results [353]. At a university hospital in Virginia (USA), the results of internal monitoring using fluorescent markers are externally validated by the manufacturer and the results are reported to the infection control committee on a monthly basis to ensure the ongoing quality [8]. A combined intervention consisting of a highly motivated and trained team, daily disinfecting surface cleaning and final monitoring in isolation units for patients with CDI reduced the number of positive environmental test results by 89% (p<0.006) [98].

In addition to the training, some centres have studied the impact of increasing staffing levels. Employing an additional worker from Monday to Friday to perform disinfecting surface cleaning of only the critical surfaces in one of 2 surgical units for a period of 6 months each (prospective cross-over design) enabled a significant reduction of the level of microbial contamination as well as the MRSA infection rate [110].

### 8.2 Hygiene plan

As part of the hygiene plan, indications, target objects, as well as cleaning and surface cleaning intervals must be defined in the cleaning and disinfection plan based on the risk area (see Table 2 (Tab. 2), Table 3 (Tab. 3), and Table 4 (Tab. 4)) and the contamination risk, and included in the service description. Coordination between the hygiene team and the service provider is recommended when developing the hygiene plan for the inpatient sector. In the outpatient sector, the head of the facility is responsible for the preparation of and compliance with the hygiene plan; external hygiene consultations may be useful depending on the facility profile. For implementing the hygiene plan, the service provider needs to establish procedural instructions with associated responsibilities, including the handling of specific procedures/technologies.

### 8.3 Implementation of monitoring

There is currently no international standard to uniformly monitor surface cleaning and surface disinfection. The recommendations of the Centers for Disease Control and Prevention (CDC) on infection control in medical facilities [143] and the recommendations derived from them for evaluating surface cleaning in hospitals [354] provide guidance. These recommendations call upon hospitals to implement programmes to improve the cleaning and disinfection of frequently touched, near-patient contact surfaces. Two levels are proposed. Level I comprises a programme which is adapted to the individual clinic and jointly established by the hygiene team and the service provider; it includes defined checklists and structured training of the cleaning staff as well as regular, standardised monitoring of the cleaning performance by the hygiene team and the service provider. Patient-satisfaction surveys are also assessed. The results are then used to develop and implement suggestions for team improvements. Level II also includes the use of objective methods for checking cleaning performance (e.g., fluorescence or adenosine triphosphate [ATP] method), which are to be carried out at least 3x annually and reported back as part of a feedback process, with the aim of achieving at least a 10–20% improvement in cleaning.

If monitoring is specifically used to improve quality, the choice of method is secondary. In terms of recovery or sensitivity, nylon-flocked swabs were superior to cellulose sponges for the detection of Gram-negative bacteria [355] or equivalent for CRE [356]. For the detection of Gram-negative bacteria, swabs are superior to contact plates; for Gram-positive cocci, the reverse is true [357]. A detailed description of the methods has been included in section 4 of the informative appendix (Attachment 1) to this recommendation, and its informative value is summarised in Table 7 (Tab. 7) without indicating any preference for one measurement method over another.

According to Ferreira et al. [358], the fluorescence method and visual inspection are good for monitoring compliance with cleaning specifications, whereas methods that check the microbiological load give a better indication of an actual risk of infection and the efficacy of disinfection. Independent of the use of a measuring method, the observation of process sequences by means of a checklist is necessary for the assessment of the quality-compliant performance of disinfecting surface cleaning. The repeat intervals are set based on the audit results.

Of course, depending on the risk profile, such extensive monitoring is not necessary in outpatient facilities, but nevertheless, quality assurance of surface cleaning and surface disinfection should also be ensured in these facilities along with continual improvement.

## 9 Recommendations

Surface cleaning, disinfecting surface cleaning according to the indication, and surface disinfection are essential standard precaution measures for the prevention of NIs. The key to the prevention potential of these measures is adherence to further standard precaution measures focusing on hand hygiene. As part of the revision of these recommendations, the evidence for several recommendations was reviewed and reassessed, and some categories were consequently adjusted. Even if no category has been assigned, there is a requirement to comply with good hygienic practices.

### Organisation and scope of surface cleaning and disinfection

**The commission recommends**

- that medical facility managers define the framework conditions for ensuring quality cleaning and disinfection measures (cat. IV).
- that the scope of cleaning (maintenance cleaning and additional cleaning services) and surface disinfection (disinfection procedures as part of standard precaution measures and targeted surface disinfection) in inpatient and outpatient healthcare facilities be defined in a service descrip-tion (no cat.) as a basis for action for external service providers, depending on the risk area involved (see Table 2 (Tab. 2)) and the risk of contamination in relation to rooms and surfaces. Coordination between the hygiene team and the service provider is recommended when developing the hygiene plan for the inpatient sector. If an external service provider is commissioned, this provider is responsible for the qualified implementation. In the outpatient sector, the head of the facility is responsible for drawing up the cleaning and disinfection plan based on the hygiene plan. If no ex-ternal service provider is involved, the head of the facility is also responsible for compliance with the cleaning and disinfection plan and for ensuring that the staff in charge are qualified (cat. IV).
- that the cleaning and disinfection plan for all surfaces specifies when, with what and how these surfaces are to be cleaned or disinfected, and also includes information on reuse (cat. IV).
  Other recommendations of the KRINKO concerning surface disinfection or disinfecting surface cleaning in special areas must also be considered, if necessary, in particular the recommendations on the prevention of SSIs, the prevention of infections in the care and treatment of patients with communicable diseases, infection prevention requirements for the medical care of immunosup-pressed patients, hygiene measures for *Clostridioides difficile* infections (CDIs) or colonisation with multidrug-resistant Gram-negative rods, and on the prevention of enterococci infections with specific antibiotic resistances, as well as recommendations for the prevention and control of methicillin-resistant *Staphylococcus aureus* (MRSA) strains in medical and nursing facilities.
- that the interfaces with the external service provider as well as the tasks that are not provided by the service provider be specified in the cleaning and disinfection plan for each department or facility (outpatient facilities) (no cat.).
- that the external service provider, in consultation with the hygiene team, establish procedural instructions including the use of specific procedures/technologies to be implemented (no cat.).
- that the external service provider establish programmes for the familiarisation and training of housekeeping staff in coordination with the hygiene team, and that the implementation of these programmes also be coordinated (no cat.).
- that cleaning and surface disinfection measures in ward and milk kitchens of health care facilities be defined by the hospital hygienist (no cat.).
- the definition of cleaning and surface disinfection measures in areas where medicinal products are manufactured or MDs are processed, in accordance with the requirements of the law on Medicinal Products and MDs (cat. IV).
- surface disinfection or disinfectant surface cleaning on surfaces on which aseptic activities are carried out prior to the start of such activities (cat. IB/IV).
- surface disinfection after visible surface contamination with potentially infectious materials (e.g., blood, secretions, excretions, faeces, etc.) after prior mechanical removal of the contamination (cat. IV).
- that surfaces that come into contact with the skin of different consecutive patients (e.g., contact surfaces of patient couches, headrests, baby scales) be disinfectant cleaned or disinfected after each use (cat. II).
- that surfaces that are frequently touched or near-patient surfaces, in areas with a potential risk of infection (see Table 2 (Tab. 2)) but where there is no evidence of colonisation or infection with critical pathogens, be disinfected daily and after patients have been discharged (cat. II).
- that frequently touched or near-patient surfaces (cat. IB) as well as floors (cat. IB) in areas with an increased risk of infection, e.g., immunocompromised patients, intensive care patients, as well as in areas with a particular risk of infection, e.g., isolation units (see Table 2 (Tab. 2)), undergo daily disinfecting surface cleaning.
- that if there are indications of frequent nosocomial transmissions or outbreaks, the measures for disinfection of surfaces with frequent hand/skin contact be reviewed as part of the intervention bundle with regard to the implementation, the selection of active substances in the disinfectant and the application conditions of the disinfectant dosing devices, and adjust them if necessary (cat. IB).
- disinfecting surface cleaning after discharge of isolated patients as terminal disinfection (cat. IB).
- the routine cleaning of all surfaces in areas without risk of infection, i.e., with a risk comparable to that of the general population (no cat.).

### Selection of surface disinfectants

**The commission recommends**

- that the hospital hygienist select the disinfectant dosing device including application concentration and contact time for the inpatient area. The head of the facility is responsible for this in the outpatient sector. Ensure that the required spectrum of activity is met (cat. IB).
- that in the selection of surface disinfectants, the activity spectrum, efficacy, material compatibility and risks to humans and the environment be considered (cat. IB).
- that rooms occupied by CDI patients be disinfected daily with a surface disinfectant with proven efficacy against *C. difficile* (cat. II); the concentration and contact time should at least be selected based on the bactericidal/levurocidal efficacy (no cat.).
- that in the event of CDI outbreaks, corridors (including handrails in the corridor) and side rooms of the ward also be disinfected with disinfectant dosing devices with proven efficacy against *C. difficile* at sporicidal concentration-time ratios (cat. II). The scope is to be defined in detail with the hospital hygienist based on the results of a departmental and patient-related risk analysis.
- that for the terminal disinfection of patient rooms, including sanitary areas, where CDI patients have been accommodated, all accessible and potentially contaminated surfaces and objects be disinfected, including the floor, after discharge, transfer or when isolation is lifted, with a disinfectant dosing device proven to be effective against *C. difficile* at a sporicidal concentration-time ratio (cat. II).
- that for infections with non-enveloped viruses with partial lipophilicity (noroviruses, rotaviruses, adenoviruses), disinfectant dosing devices declared as limited virucidal PLUS be used, and for hydrophilic non-enveloped viruses (e.g., picornaviruses), disinfectant dosing devices declared as virucidal be used (cat. II).
- that in the case of mould contamination, after remediation of moisture damage and subsequent detailed cleaning in consultation with the hygiene staff, surface disinfectants with fungicidal activity be used (cat. II).
- not using QAC-based surface disinfectants at the low 4-hour concentration because of the risk of developing resistance (no cat.).
- that, as a matter of principle, surface disinfectants be used under the application conditions that were used to determine their efficacy under practical conditions (cat. II).

### Implementation

**The commission recommends**

- that the surface to be disinfected be wetted with an appropriate amount of disinfectant solution (no cat.); that the applied disinfectant not be removed by wiping with water or dry wiping before the end of the contact time (no cat.).
- that working solutions (no cat.) be handled appropriately, protected from contamination, and that their shelf lifebe respected (cat. IV).
- the use of fresh and clean wiping textiles (no cat.); alternatively, wiping textiles soaked with disinfectant solution removed from disposable packaging or pre-soaked wiping textiles from processable wipe dispenser systems or from the washing machine (ready-made) are also suitable.
- the implementation of application methods that do not involve the reimmersion of used wiping textiles into the cleaning or disinfectant solution (no cat.); reimmersion is strictly forbidden (cat. II).
- that any visible contamination with organic material first be removed mechanically (without the use of disinfectant) and the surface then disinfected (two-step procedure) (cat. IV).
- the proper processing and dry storage of auxiliary materials used (e.g., wipe dispenser systems, colour-coded containers, tubs, wipe and mop head holders, presses, insert sieves, cleaning trolleys, hand contact surfaces) (no cat.).
- that the processing of wiping textiles used for both cleaning and disinfection be performed separately from other processed laundry on account of the level of soiling, the nature of the material (e.g., detaching of textile fibres) and the risk of introducing extraneous chemicals (no cat.).
- that textiles used for cleaning be processed in a household washing machine and dried in a household tumble dryer (no cat.); on the other hand, reusable wiping textiles used for disinfection must be processed in a disinfection washing process in order to safely remove dirt and organic contamination from the rinsing, cleaning and wet wiping covers and to ensure that pathogens are no longer detectable (cat. II).
- the machine drying of processed wiping textiles (no cat.). Unless pre-soaked wiping textiles are used, the completely dried wiping textiles should be stored in a dry place until further use to prevent the propagation of any potential residual flora. Processed wiping textiles must be protected from recontamination (no cat.).
- the use of disposable wiping textiles, if wiping textiles used for disinfection cannot be processed as part of the disinfection washing process (cat. II).
- carefully weighing the use of any special processes such as machine pre-soaking of wiping textiles (processing) as part of a risk analysis, due to the variety of potential influencing factors and that the efficacy of any such processes should be checked (no cat.).
- that reprocessable wipe dispenser systems not be used in areas where there is an increased risk of infection (cat. II). This does not apply to processable wipe dispenser systems that use alcohol-based disinfectants.
- that when using reprocessable wipe dispenser systems, the manufacturer’s declared period of use (available expert confirmation) and reprocessing of the system (with a bactericide, fungicide and sporicide) absolutely be complied with before reloading (cat. IV); the dispenser system must be carefully closed after the removal of each wipe.
- that reprocessable wipe dispenser systems be checked hygiene-microbiological in the event of nosocomial outbreaks, particularly if Gram-negative bacteria are involved (cat. II); if necessary, an additional annual random hygiene-microbiological inspection may be considered in order to check the processing.
- limiting the use of alcohol-based surface disinfectants to small circumscribed areas due to their inflammability; the total quantity applied per room must not exceed 50 ml per m^2^ room floor area (cat. IV).
- observing the contact time declared by the manufacturer for the application concentration:
  - for work surfaces before aseptic activities,
  - for visible contamination with potentially pathogen-containing material (e.g., blood, secretions, excretions),
  - for contamination from environmental sources,
  - for terminal disinfection,
  - for patients’ bathtubs until the next time the bath is filled with water,
  - for the ward kitchen, when rinsing with drinking water is required after disinfection,
  - for the application of surface disinfectants, specifically against viruses or bacterial spores (or, if applicable, for other spectra of activity), for which the conditions of application have been determined exclusively based on suspension tests.

If surfaces need to be reused quickly on a regular basis, it makes sense to use disinfectant dosing devices with a short contact time adapted to the practical situation. All other surfaces can be walked on/used after drying (no cat.).

### Selection of procedures

**The commission recommends**

- that only areas that cannot be reached by wipe disinfection be disinfected by spraying (cat. IV).
- the use of equipment for nebulising H_2_O_2_ in addition to regular surface disinfection, but only in the case of NI outbreaks where routine wipe disinfection methods did not stop the outbreak (cat. II); their use requires evidence that the required H_2_O_2_ concentration is achieved in the ambient air for the duration of the contact time at the concentration previously established by an expert in order to guarantee the disinfection effect. Staff may only access the room when the H_2_O_2_ concentration has fallen below the OEL (0.5 ml/m^3^ or 0.7 mg/m^3^) (cat. IV). Patients may only be admitted to the room when the H_2_O_2_ concentration has fallen below the DNEL (1.93 mg/m^3^) (cat. IV). It should be noted that efficacy is not achieved for blood residues and that efficacy has not been specified for fluid accumulations (cat. II).
- that machine methods for surface cleaning only be used in areas without a risk of infection due to the lack of evidence of effectiveness (no cat.).
- that physical non-contact equipment-based methods not be used in areas with an increased or particular risk of infection due to the lack of test standards for disinfecting effect and the resulting uncertain efficacy (no cat.).

### Building requirements, material compatibility and surface design

**The commission recommends**

- that hygienically relevant surfaces, including floors, be reliably cleanable and disinfectable and that no material damage be inflicted by the cleaning or disinfecting surface cleaning (no cat.). Prior to purchasing new products, e.g., floor coverings and furniture, we recommend confirming with the manufacturer the material compatibility in terms of suitability for disinfection.
- that surfaces impregnated with anti-adhesive and antimicrobial products not be used to supplement basic hygiene precautions with regard to disinfecting surface cleaning, as long as their infection prevention benefits have not been assessed in terms of epidemiology and the risks they pose to humans and the environment as well as their ability to induce microbial resistance have not been evaluated (cat. III).
- that ventilated rooms for processing and storage of auxiliary materials be available in the inpa-tient area, either as spatially separate clean and unclean rooms, or, the size of the room permitting, at least as separate clean and unclean areas; in the outpatient area, facility-specific measures need to be implemented (no cat.).
- that depending on the size of the facility and the way in which the disinfectant solution is prepared, automatic decentralised disinfectant dosing devices be installed (no cat.); with regard to dosing accuracy, the dosing devices should comply with the recommendations issued by the Federal Institute for Materials Research and Testing (BAM), the RKI and KRINKO; at the same time, the operating principle of the device must not be conducive to forming biofilms (no cat.).
- that in the case of nosocomial outbreaks, the hygiene-microbiological inspection of the disinfectant dosing device be decentralised (no cat.); irrespective of this, the frequency of the technical inspection is based on the manufacturer’s specifications (cat. IV).
- that in the event that a dosing device is not used, other safe dosing methods be implemented which are simple, reliable and less prone to errors; the correct application must be ensured and verified (no cat.).

### Quality assurance of surface cleaning and disinfection

**The commission recommends**

- that the staff and equipment required to ensure quality cleaning and disinfecting surface cleaning, including processing technology, be provided (no cat.); that the quality and continual improvement of implementation be assured through familiarisation programmes as well as regular documented training and education measures (cat. II); that the procedure be adapted to the size of the facility (no cat.).
- the quality of surface cleaning and disinfection be ascertained by means of facility-related hygiene monitoring (cat. II); the scope of monitoring and the responsibility for its implementation, assessment and frequency as well as the communication channels in the inpatient area are to be deter-mined by the hospital hygienist and the head of the facility for outpatient areas, where applicable in coordination with an external hygiene advisor (no cat.).
- that the inspection of the disinfecting surface cleaning as part of terminal disinfection after the occurrence of particular pathogens be carried out on an ad hoc basis through hygiene monitoring (no cat.).
- that if reusable textiles are used for disinfection and processed, an annual validation be conducted to determine the effectiveness of the disinfecting washing process in the respective facility (no cat.). In the event of an outbreak, a hygiene-microbiological inspection of the sources in question is indicated (no cat.).

These recommendations were produced on behalf of the Commission for Hospital Hygiene and Infection Prevention by Prof. Dr. Axel Kramer (Head of the working party), Dr. Bärbel Christiansen, Prof. Dr. Martin Exner, Prof. Dr. Ursel Heudorf, Prof. Dr. Lutz Jatzwauk and Prof. Dr. Constanze Wendt on a voluntary basis and without influence from commercial groups. From the Robert Koch Institute, Dr. Franziska Lexow, Dr. Ingeborg Schwebke (formerly from RKI) and Marc Thanheiser were involved. The recommendations were prepared by the working party and, after a detailed discussion, agreed by the Commission.

## List of abbreviations

- *A/C* Air conditioning
- *ATP* Adenosine triphosphate
- *AOX* Adsorbable organic halides
- *BAC* Benzalkonium chloride
- *BPR* Biocidal products regulation
- *CA-MRSA* Community acquired MRSA
- *CDI* *Clostridioides difficile* infection
- *cm^2^* Square centimetre
- *COPD* Chronic obstructive pulmonary disease
- *CRE* Carbapenem-resistant enterobacteriaceae
- *DDAC* Didecyldimethylammonium chloride
- *DNEL* Derived no-effect level
- *DVG* German Society of Veterinary Medicine
- *DVV* German Association for the Control of Viral Diseases
- *H_2_O_2_* Hydrogen peroxide
- *HINS* High-intensity narrow-spectrum
- *IfSG* Infection Protection Act
- *ICU* Intensive care unit
- *ISSA* International social security association
- *CFU* Colony forming unit
- *KRINKO* Commission for Hospital Hygiene and Infection Prevention
- *m^2^* Square metre
- *m^3^* Cubic metre
- *Mg* Milligram
- *log_10_* Decimal logarithm
- *Min* Minute
- *Ml* Millilitre
- *MD* Medical device
- *MDRO* Multidrug-resistant organisms
- *MRSA* Methicillin-resistant *Staphylococcus aureus*
- *NI* Nosocomial infection
- *OEL* Occupational exposure limit
- *PAA* Peracetic acid
- *Ppm* Parts per million
- *QAC* Quaternary ammonium compound
- *RKI* Robert Koch Institute
- *TRBA* Technical rules for biological agents
- *TRGS* Technical rules for hazardous substances
- *VAH* Association for Applied Hygiene
- *VRE* Vancomycin-resistant enterococci

## Notes

### Competing interests

The author declares to have no competing interests.

## Supplementary Material

Electronic supplementary material (only online and in German)

## Figures and Tables

**Table 1:**
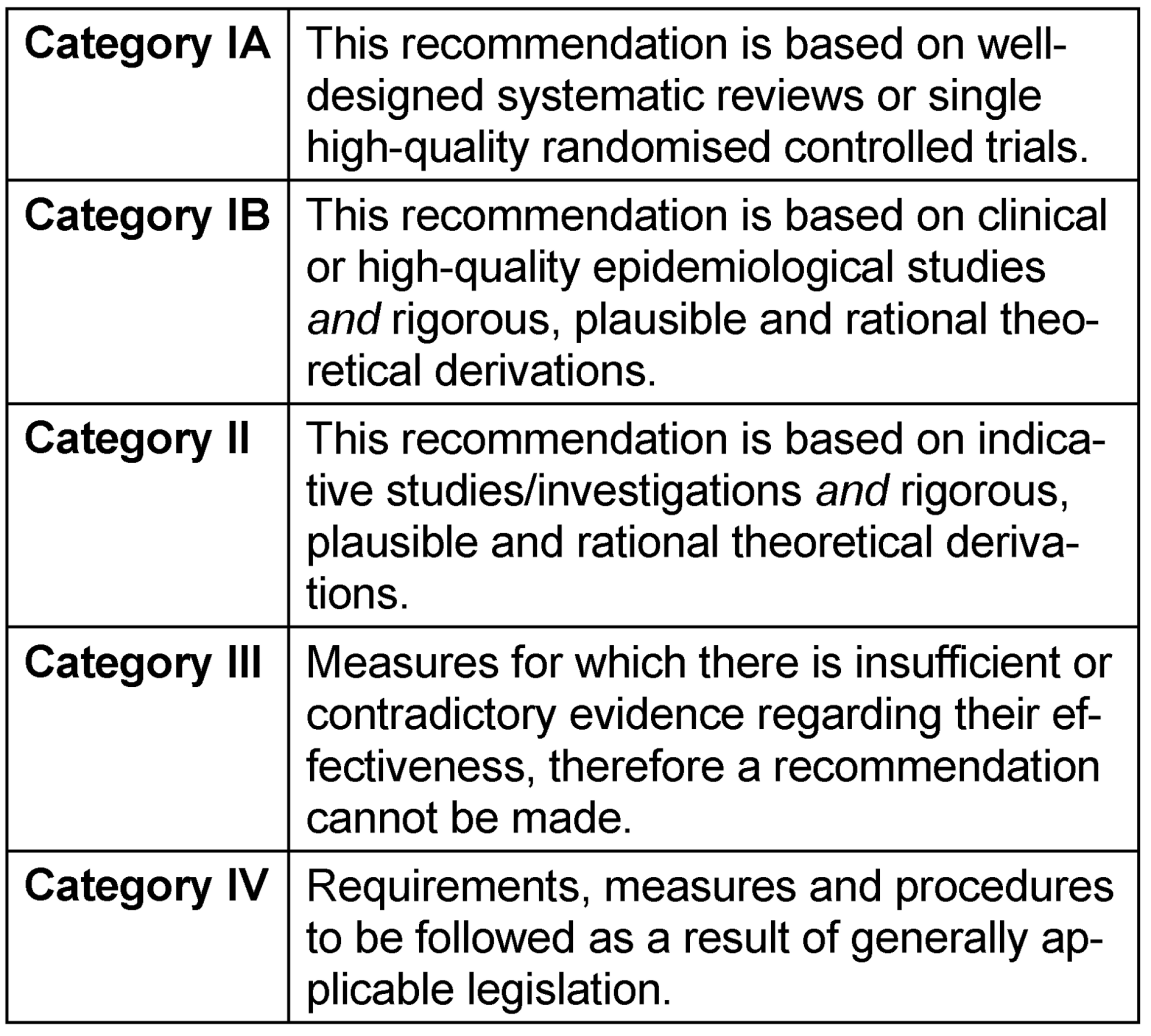
Categories within the recommendations of the Commission for Hospital Hygiene and Infection Prevention (KRINKO, 2010)

**Table 2:**
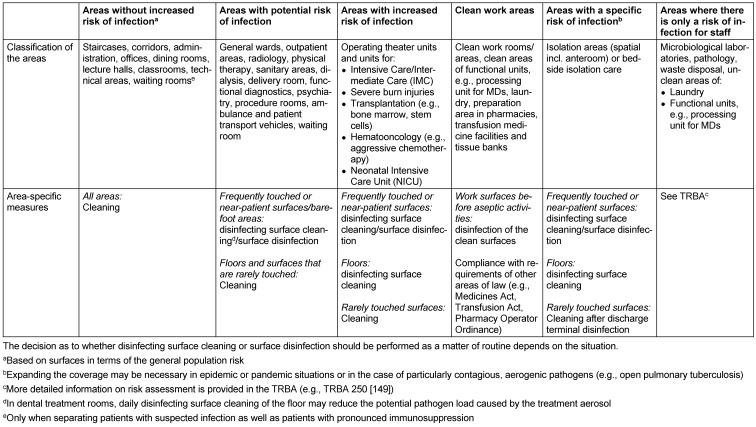
Cleaning and disinfection measures based on the risk of infection for patients and staff (the list within columns serves as an example)

**Table 3:**
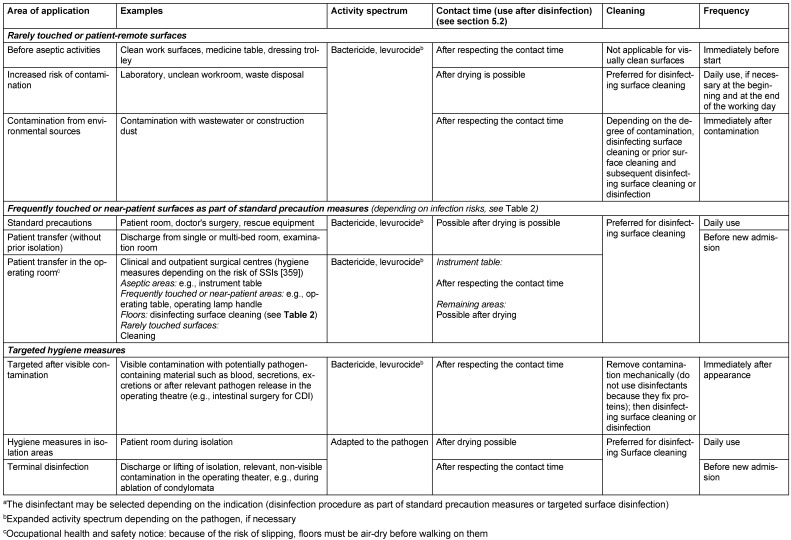
Areas of application for surface disinfection or disinfectant surface cleaning with reference to exposure time and frequency of application^a^

**Table 4:**
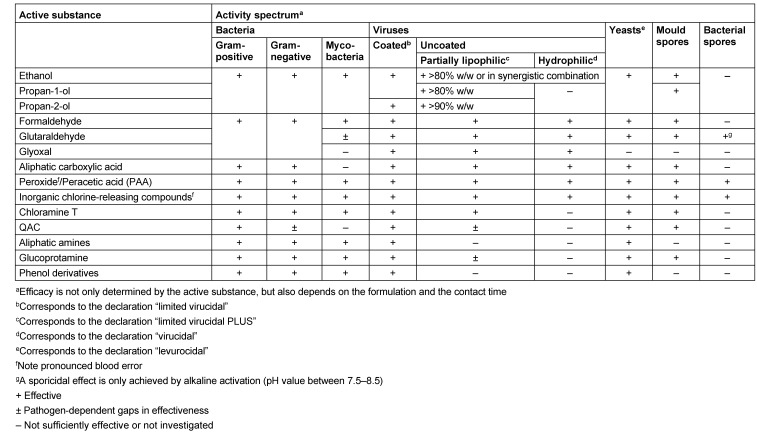
Indicative data on the activity spectrum of microbicidal active substances or substance classes for surface disinfection

**Table 5:**
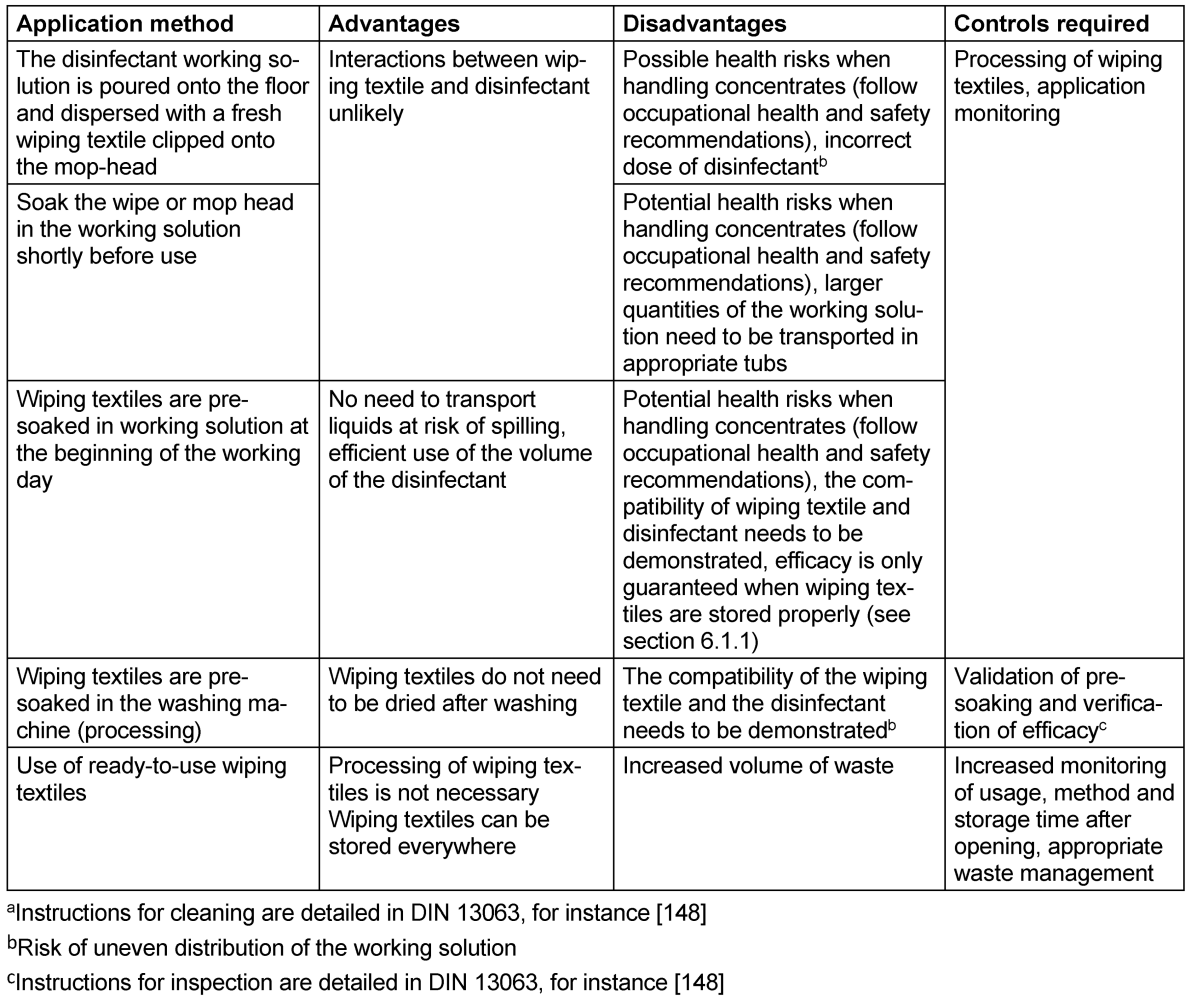
Advantages and disadvantages of different wiping methods for manual disinfecting cleaning and disinfection^a^ of floors

**Table 6:**
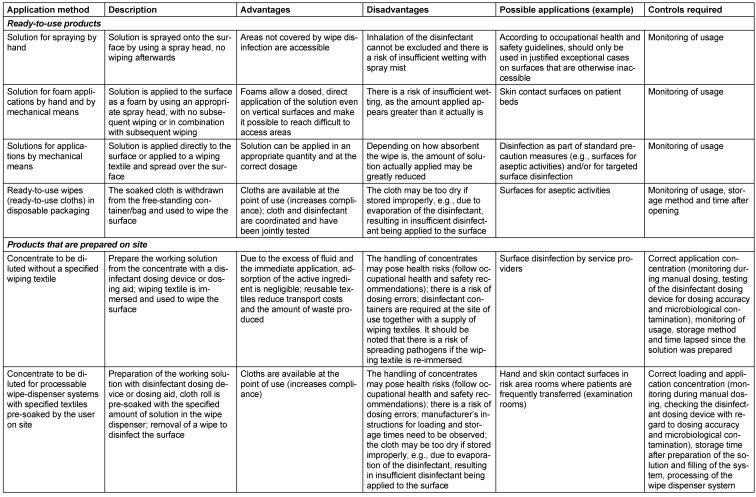
Advantages and disadvantages of different methods for disinfecting surface cleaning and surface disinfection of small surfaces

**Table 7:**
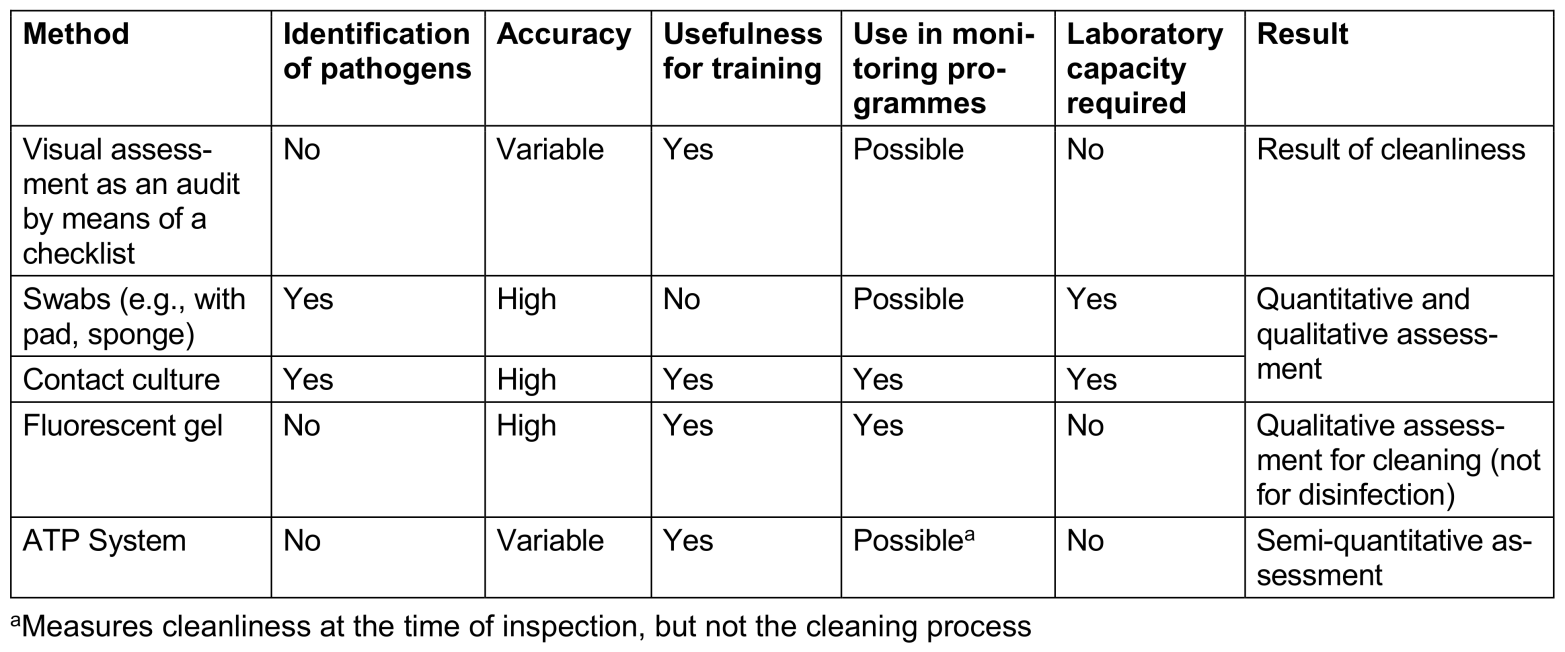
Comparison of different methods for assessing the results of disinfecting surface cleaning of the patient environment (modified according to [358])
