# Supplementary material for: Hygiene requirements for cleaning and disinfection of surfaces: recommendation of the Commission for Hospital Hygiene and Infection Prevention (KRINKO) at the Robert Koch Institute
Source: GMS Hyg Infect Control. 2024 Mar 5;19:Doc13. doi: 10.3205/dgkh000468 (PMC11035912; doi:10.3205/dgkh000468)
Supplement: Electronic supplementary material (only online and in German) [file HIC-19-13-s-001.pdf]

Informativer Anhang zur Empfehlung „Anforderungen an die Hygiene bei der Reinigung und Desinfektion von Flächen“ der Kommission für Krankenhaushygiene und Infektionsprävention (KRINKO) beim Robert Koch-Institut

Inhaltsverzeichnis

Abkürzungsverzeichnis ... 2

1 Einführung ... 4

2 Für die Indikationsabwägung der desinfizierenden Flächenreinigung relevante Erregereigenschaften ... 5

2.1 Tenazität von Mikroorganismen und Viren auf unbelebten Flächen ... 5

2.1.1 Bakterien ... 6

2.1.2 Pilze ... 42

2.1.3 Viren ... 51

2.2 Infektiöse Dosis ... 76

2.3 Biofilmbildung ... 76

3 Toxikologische und ökotoxikologische Merkmale mikrobizider Wirkstoffe ... 77

4 Methoden zur Bewertung der Ergebnisqualität der Reinigung bzw. desinfizierenden Flächenreinigung ... 80

4.1 Audit ... 80

4.2 Mikrobiologische Beprobung ... 80

4.3 ATP-Methode ... 81

4.4 Fluoreszenzverfahren ... 82

4.5 Optische Kontrolle ... 83

5 Prüfmethoden zur Bestimmung der Wirksamkeit von Flächendesinfektionsverfahren ... 84

Literatur ... 86

Allgemeine Anmerkung:
Grundsätzlich sind in diesem Dokument bei allen Berufs- bzw. Gruppenbezeichnungen immer alle Geschlechter gemeint.

Attachment to: Commission for Hospital Hygiene and Infection Prevention (KRINKO). Hygiene requirements for cleaning and disinfection of surfaces: recommendation of the Commission for Hospital Hygiene and Infection Prevention (KRINKO) at the Robert Koch Institute. GMS Hyg Infect Control. 2023;18:Doc13. DOI: 10.3205/dgkh000468

Abkürzungsverzeichnis

ATCC	American type culture collection
ATP	Adenosintriphosphat
BSA	Bovines Serumalbumin
$CaCl_2$	Kalziumchlorid
$CCID_{50}$	Virusmenge, die 50 % der inokulierten Zellkulturen infiziert (cell culture infectious dose 50; auch kulturinfektiöse Dosis 50 (KID_{50}) oder tissue culture infectious dose 50 ($TCID_{50}$))
cm	Zentimeter
cm^2	Quadratzentimeter
CPE	Zytopathogener Effekt
cpm	Zerfälle pro Minute (counts per minute)
d	Tag
D-Wert	Dezimale Reduktionszeit
dest.	destilliert
h	Stunde
H_2O	Wasser
Hz	Hertz (Einheit)
IE	Infektiöse Einheiten
ITS	Intensivtherapiestation
k.A.	keine Angabe
KbE	Koloniebildende Einheiten
KT	Keimträger
KRINKO	Kommission für Krankenhaushygiene und Infektionsprävention
lg	Dekadischer Logarithmus
Lsg.	Lösung
m^3	Kubikmeter
mg	Milligramm
min	Minute
ml	Milliliter
µg	Mikrogramm
µl	Mikroliter
µm	Mikrometer

Mon	Monat
MSSA	Methicillin-sensitiver *Staphylococcus aureus*
MRSA	Methicillin-resistenter *Staphylococcus aureus*
N_t/N_0	Initialer Virus-Titer/Titer zum angegebenen Zeitintervall
NaCl	Natriumchlorid
n.b.	nicht berichtet
n.n.	nicht nachweisbar
PBS	Phosphat gepufferte Salzlösung (phosphate-buffered saline)
PCR	Polymerase-Kettenreaktion (polymerase chain reaction)
PFU	Plaquebildende Einheiten (plaque forming units)
PPS	Physiologische Pepton-Salz-Lösung
PVC	Polyvinylchlorid
QAV	Quartäre Ammoniumverbindungen
RIA	Radioimmunoassay
RK	Raumklima
RLF	Relative Luftfeuchtigkeit
RLU	Relative Lichteinheiten
RT	Raumtemperatur
s	Sekunde
TK	Testkörper
TSB	Trypticase-Soja-Brühe
US	Ultraschall
VBNC	viable but non-culturable
VISA	Vancomycin-intermediär-sensibler *Staphylococcus aureus*
VRE	Vancomycin-resistente Enterokokken
vs.	versus
W	Watt (Einheit)
Wo	Woche

1 Einführung

Krankheitserreger können von Oberflächen direkt oder indirekt einschließlich nach Verwirbelung über die Raumluft [1] akquiriert werden [2] und abhängig von der Erregermenge, der Infektionsdosis, der Immunitätslage und der Eintrittspforte eine Infektion verursachen. Ausgehend von der Umgebungskontamination sind Infektionen nach Neubelegung von Patientenzimmern [3] und Ausbrüche beschrieben worden [4, 5]. Insbesondere aus dem *Vestibulum nasi*, dem Respirationstrakt und dem Gastrointestinaltrakt [6-8] wird die patienteneigene Flora in die patientennahe Umgebung freigesetzt. Die Kenntnis der Überlebensfähigkeit (Persistenz bzw. Tenazität) von Mikroorganismen bzw. der Dauer des Erhalts der Infektiosität von Viren auf Flächen sowie die erforderliche Infektionsdosis bei der Akquirierung von Krankheitserregern auf Flächen ermöglichen eine orientierende Risikoabschätzung des von Flächen ausgehenden Infektionsrisikos und der daraus ableitbaren Indikationen der desinfizierenden Flächenreinigung. Deshalb wurden Angaben zur Wiederanzüchtbarkeit von Mikroorganismen und Viren auf Flächen als informativer Anhang zusammengestellt, um Interessierten weiterführende Informationen zur Verfügung zu stellen und zugleich den Umfang der KRINKO-Empfehlung „Anforderungen an die Hygiene bei der Reinigung und Desinfektion von Flächen“ zu begrenzen. Ein weiteres Kriterium für die Indikation zur Reinigung bzw. desinfizierenden Reinigung ist die Verhinderung einer Biofilmbildung auf Flächen. Deshalb wird darauf hingewiesen, welche Mikroorganismen unter welchen Bedingungen schwer entfernbare Biofilme bilden.
Angaben zu Risiken der zur Flächendesinfektion eingesetzten Wirkstoffe für Mensch und Umwelt sollen eine Hilfestellung für die Auswahl der Desinfektionsmittel und deren Anwendung geben und wurden deshalb ebenfalls in den informativen Anhang aufgenommen.

Ebenfalls wurden die zur Auswahl stehenden Methoden zur Bewertung der Ergebnisqualität der Reinigung und desinfizierenden Reinigung (sog. Hygienemonitoring) in den informativen Anhang aufgenommen.

Bei den Angaben in den **Tabellen 1-7** dieses informativen Anhangs handelt es sich um beispielhafte Aufzählungen, die Anwendern der KRINKO-Empfehlung eine zusätzliche theoretische Orientierung bieten sollen, ohne Anspruch auf Vollständigkeit.

Es sei an dieser Stelle noch einmal betont, dass im informativen Anhang keine Empfehlungen für die klinische Anwendung ausgesprochen werden.

2 Für die Indikationsabwägung der desinfizierenden Flächenreinigung relevante Erregereigenschaften

2.1 Tenazität von Mikroorganismen und Viren auf unbelebten Flächen

Unter dem Begriff der Tenazität wird die Widerstandsfähigkeit von Mikroorganismen und Viren gegenüber Umwelteinflüssen verstanden. Da Bakterien einen eigenen Stoffwechsel haben, wird hier in der Literatur synonym der Begriff der Persistenz, d.h. der Überlebensfähigkeit, gebraucht. Die Persistenz von Mikroorganismen ist Folge der Anpassungsreaktion, in der Umwelt durch Reduktion des Metabolismus sowie durch morphologische, biochemische und/oder genetische Adaptationen zu überleben, insbesondere bei Bakterien in ihrer Hauptlebensform der Biofilme [9-11]. Eine weitere Form der Anpassung ist der Übergang zu viable but non-culturable (VBNC)-Zellen, die nur durch bestimmte Stimuli wieder in ein reproduktives, virulentes Stadium überführt werden können [12, 13]. Kriterium zur Ermittlung der Persistenz von Mikroorganismen ist die Rekultivierbarkeit des Erregers nach Aufbringung auf eine Fläche.

Da Viren keinen eigenen Stoffwechsel haben, benötigen sie für ihre Replikation den Syntheseapparat intakter Wirtszellen. Deshalb wird anstelle des Merkmals der Überlebensfähigkeit der Begriff der Tenazität verwendet, der die Stabilität von Viruspartikeln unter Beibehaltung der Infektiosität in der unbelebten Umgebung beschreibt. Die Überlebensfähigkeit wird anhand der Anzahl infektiöser Viruspartikel durch Anzucht der verbliebenen Viruspartikel üblicherweise in Zellkulturen mit anschließender Ermittlung des Virustiters bestimmt. Dagegen lässt der alleinige molekularbiologische Nachweis keinen Rückschluss auf die Infektiosität zu. Deshalb werden nachfolgend nur Studien berücksichtigt, in denen die Rekultivierbarkeit von Viren ermittelt wurde. Üblicherweise wird zunächst eine Resuspension von der Testfläche vorgenommen, die dann in die Zellkultur überführt wird. Unter Viruspersistenz wird dagegen verstanden, dass die genetische Information von Viren in Zellen des Wirtsorganismus vorliegt und das Virus unter bestimmten Umständen, z.B. bei Immunsuppression des Wirts, reaktiviert werden kann (bspw. bei Herpesviren).

Mit höherer Menge und zunehmender intrinsischer Umweltpersistenz bzw. Tenazität steigt die Wahrscheinlichkeit des von kontaminierten Flächen ausgehenden nosokomialen Übertragungsrisikos, wenn die Krankheitserreger nicht entfernt bzw. inaktiviert werden. Erwartungsgemäß ist sowohl für Bakterien [8, 14] und *Candida (C.)* spp. [15] als auch für Viren die Übertragbarkeit von Händen auf Flächen und umgekehrt nachgewiesen [8, 14, 16].

Bei der Bewertung von Angaben zur Persistenz bzw. Tenazität ist zu berücksichtigen, dass diese in den meisten Untersuchungen auf trockenen Oberflächen, unterschiedlichen Materialien und bei unterschiedlichen abiotischen Umgebungsbedingungen (Temperatur, relative Luftfeuchtigkeit,

Eiweißbelastung, Belichtung, pH-Wert [17]) nach experimenteller Kontamination unter Laborbedingungen untersucht wurden. Häufig handelt es sich nicht um Medianwerte, sondern um die maximal nachgewiesene Tenazität. Ferner ist zu berücksichtigen, dass sich bei hohen Ausgangszahlen aufgrund der logarithmischen Absterbekurve [18] längere Überlebenszeiten ergeben, was für verschiedene Bakterienspezies [19] und für Sprosspilze [20] auf Flächen nachgewiesen wurde.

Aus den genannten Gründen können Laborstudien nur eine Orientierung geben, zumal die klinische Situation der häufig gleichzeitigen Kontamination von Krankenhausoberflächen mit verschiedenen Erregern, Sekreten, Exkreten, Staub und antimikrobiellen Rückständen, z.B. von der letzten Flächendesinfektion nicht erfasst wird.

2.1.1 Bakterien

Je nach Bakterienspezies und Umgebungsbedingungen beträgt die Überlebensfähigkeit Tage (d) bis Monate (Mon) (**Tab. 2**). Häufig haben Gram-negative Bakterien eine geringere Persistenz als Gram-positive Bakterien. Sporen von Bacillus und Clostridien überleben abhängig vom Material > 6 Mon (**Tab. 2**).

Auf Kupferoberflächen ist die Persistenz von Bakterien, Pilzen und die Dauer der Infektiosität von Viren deutlich kürzer als auf textilen Materialien, Kunststoffen und Stahl [21, 22].

Die Überlebenszeit wird von der Menge des Ausgangsinokulums, dem Material des Prüfkörpers und damit von der kontaminierten Oberfläche in der Gesundheitseinrichtung, der Art der Rückgewinnung, der Antrocknung auf der Oberfläche (rasch oder langsam), der relativen Luftfeuchtigkeit (RLF) und der Temperatur während der Lagerung, den Rekultivierungsbedingungen und dem Stadium der Kultivierbarkeit (VBNC) beeinflusst (**Tab. 2**).

Gram-positive Bakterien tolerieren aufgrund ihrer Zellwand trockene Bedingungen besser als Gram-negative Bakterien [23]. *Staphylococcus* (*S.) aureus* persistierte länger bei geringer relativer RLF [24], während die Überlebenskinetik für *Enterococcus (E.) faecalis* bei 25 % RLF geringer war als bei 0 % RLF [25]. In destilliertem (dest.) Wasser suspendierte *Acinetobacter* spp. überlebten bei Raumtemperatur (RT) signifikant länger bei einer RLF von 28-34 % bzw. 93 % im Vergleich zu 10 % RLF, während sich die Überlebensrate zwischen 28-34 % bzw. 93 % nicht unterschied [26]. Die Überlebensfähigkeit Gram-positiver Bakterien wurde am meisten bei einer RLF von 50-70 % reduziert, während die Absterberate bei Gram-negativen Bakterien am höchsten bei einer RLF von 50-70 % bzw. 70-90 % war [23].

Antrocknung in eiweißhaltigen Medien verlängert die Persistenz, nachgewiesen, z.B. für *Acinetobacter* (*A.) baumannii* (**Tab. 1**), *Escherichia (E.) coli* [26], *Neisseria (N.) meningitidis* und Sprosspilze [20, 27].

Tabelle 1: Persistenz unterschiedlicher *A. baumannii*-Stämme bei Antrocknung in Wasser bzw. bovinem Serumalbumin (BSA) nach Jawad et al. [26]

Durchschnittliche Persistenz	Stamm	Bedingungen
≤ 5 Tage (d)	American type culture collection (ATCC) 9955	suspendiert in Wasser; 28-34 % RLF, 20-24 °C
6-10 d	ATCC 17978, ATCC 19606, R 0211019	
> 10-29 d	ATCC 17904, 18, 49, 16/48, 16/49, R 447	
6-10 d	ATCC 9955	suspendiert in 7 % BSA
> 10-31 d	ATCC 17978, 18, 16/48,	
> 29-59 d	ATCC 19606, ATCC 17904, 49, 16/49, R 447, R 0211019	

Konstante Temperaturen > 24 °C scheinen das Überleben von Bakterien, nachgewiesen für Vertreter Gram-positiver, Gram-negativer und intrazellulärer Bakterien, in der Luft zu verringern [23].

Schließlich ist auch die Herkunft des Erregers von Einfluss. So waren aus klinischen Settings isolierte *A. baumannii*-Stämme häufiger widerstandsfähiger gegen Austrocknung als ATCC-Stämme (**Tab. 1**).

Die Relevanz des Vorkommens auf Krankenhausoberflächen findet ihren Ausdruck in der zunehmend nachgewiesenen Assoziation zwischen Umgebungskontamination und Infektionsrisiko (siehe Abschnitt 2.3 der Empfehlung „Anforderungen an die Hygiene bei der Reinigung und Desinfektion von Flächen“).

Tabelle 2: Persistenz von Bakterien und Bedingungen zur Rekultivierung

Erreger	Ausgangs-inokulum	Rückgewinnung zur Kultivierung	Tenazität	Oberfläche	Temperatur/ RLF	Bemerkung	Quelle
**Gram-positiv**							
Bacillus subtilis-Sporen	~ 8 lg kolonie-bildende Einheiten (KbE)/Milliliter (ml)	Keimträger (KT) 15 Minuten (min) Schütteln in Phosphat gepufferte Salzlösung (PBS) + Triton X-100, danach Ultraschall (US) 45 min; Bestätigung durch Polymerase-Kettenreaktion (PCR)	nach 15 d Reduktion ~ 0,3 lg, nach 56 d Reduktion um ~ 0,7 lg	Glas	21-23 °C/36 %	Bestätigung durch PCR	[28]
	7,1-9,5 lg KbE/ml, Applikation von 20 Mikroliter (µl)	Filter in 0,89 % Natriumchlorid (NaCl) 30 Sekunden (s) vortexen (zweimal im Abstand von 30 min), Eluat auf Agar	> 200 d (Abnahme um ~ 2 lg)	Polycarbonatmembran-filter	22 °C/31 %	Stamm JH642; Wiedergewinnungs-rate vom Membranfilter 5,4-7,8 lg KbE/ml; Überlebensrate ist nur orientierend zu entnehmen	[29]
Clostridioides (C.) difficile-Sporen	6 lg KbE/ml	Kontaktkultur	nach 2 d Reduktion um ~ 2 lg, nach 4 Wo 8 KbE, nach 5 Mon 1 KbE/feet(ft)^2 (Einheit; 1 ft = 30,48 cm)	Fußboden	keine Aussage (k.A.)	Bestätigung biochem./chromat.; Studie in leerem Krankenzimmer	[30]
	6-7 lg KbE/KT	KT in dest. Wasser, US 60 Hz/20 min, Eluat auf Agar	6 Wo Reduktion um ~ 0,5-0,8 lg; 12	Stahl	21-27 °C/40-63 %	Lufttrocknung	[31]

			Wo Reduktion um < 3 lg				
C. difficile veg.	~ 6 lg KbE/ml	Abstrich (PBS + Taurocholsäure)	15 min, Reduktion um ~ 4 lg	Glas	RT/k.A.	Nachweisgrenze 3-4 lg; trockene Fläche	[32]
Corynebacterium diphtheriae	je nach Probenge-winnung bis 155 KbE/30,5 Qua-dratzentimeter (cm^2)	kulturell, biochemisch	7-90 d (abhängig vom Stamm)	Staub aus Patientenzimmer, Rekultivierung bis 30 d nach Kontamination in Teströhrchen	RT/k.A.		[33]
Corynebacterium pseudotuberculosis	~ 6 lg KbE/ml	trockener Baumwolltupfer, kulturell	3 d /1,1 d /4,1 d	Kunststoff	22 °C/37 °C/4 °C		[34]
Enterococcus faecium	6-7 lg KbE/KT	KT in dest. Wasser, US 60 Hertz (Hz)/20 min, Eluat auf Agar, Rekultivierung	nach 12 Wo, Reduktion um < 3 lg	Stahl	21-27 °C/40-63 %	Teststamm NCTC 12204; Lufttrocknung	[31]
	~ 6,5 lg KbE Testfläche	Testfläche auf Agar, Rekultivierung	49 d/51 d/49 d/49 d (qualitativ)	Baumwolle/Baumwolle-Polyester/Wolle /Seide	21-24 °C/30-49 %	klinische Isolate, Krankenhaustextilien	[35]
	~ 8,4 lg; Applikation 0,25 µl/25 cm^2	Kontaktkultur von kontaminierter Fläche	250 KbE nach 7 d, ~ 240 KbE nach 14 d, 162 KbE nach 28 d /250 KbE nach 7 d, ~ 186 KbE nach 14 d, ~ 32 KbE nach 28 d/250 KbE nach 14 d, ~ 56 KbE nach 21 d, 50 KbE nach 28 d/250 KbE nach 7 d, ~ 125 KbE nach 14 d, ~ 18 KbE nach 28 d	Glas/Polyvinylchlorid (PVC)/Stahl/Aluminium	21 °C/31-35 %	Teststamm ATCC19434, angetrocknet in 0,89 % NaCl-Lösung	[36]

	8 lg KbE/ml	5 min geschüttelt in 100 ml 0,9 %iger NaCl-Lösung mit Glasperlen, Membranfiltration und/oder Verdünnungsreihen, Rekultivierung	1 bis 16 Wo	PVC	22 ± 2 °C/50 ± 5 %	einige Enterokokkenstämme überleben unter trockenen Bedingungen > 4 Mon	[37]
	8 lg KbE/ml	5 min in 100 ml NaCl Vortexen auf Glasperlen, Membranfiltration/ Rekultivierung	bis 4 Mon (~ 2 lg rekultivierbar)	Keramik/PVC/Gummi/Stahl (5 x 5 cm)	22 ± 2 °C/50 ± 5 %	Teststamm ATCC 19433; Wiedergewinnungs-rate vom Membranfilter 12-96 % (im Mittel 46 %)	[38]
	~ 5 lg KbE/ml	Resuspension in Thioglycolatbouillon	33/29/29/> 90/> 90 d	Baumwolle/Baumwoll-frottee/Mischgewebe aus Baumwolle und Polyester/Polyester/Poly-propylen-Kunststoff	22,9-24,5 °C/30-49 %	Herkunft von Patienten oder aus der Krankenhausumge-bung	[39]
Enterococcus faecalis	6-7 lg KbE/KT	KT in dest. Wasser, US 60 Hz/20 min, Eluat auf Agar, Rekultivierung	nach 6 Wo, Reduktion um < 1,8 lg	Stahl	21-27 °C/40-63 %	Lufttrocknung	[31]
	7,5 lg KbE/ml (1:1) mit Urin, Blut, Salzlösung (lsg.) Wasser; Applikation von 20 µl	Resuspension in Trypticase-Soja-Brühe (TSB), Eluat auf Agar, Kultivierung	alle KT: Abnahme um ~ 1 lg KbE/ml nach 8 Wo	Keramikboden/Baumwoll-stofffragmente/synthe-tische Fasern/Eierkarton-Matratzenauflage	RT/k.A.	KT 3 cm^2	[40]
	5,2 lg KbE/ Baumwolltuch (2x2 cm)	in 4 ml PBS gemixt, Eluat auf Agar, Rekultivierung	nach 1 d Überleben von 3 % der KbE	Baumwolltuch	21± 1 °C /50 ± 5 %		[41]

	~ 5 lg KbE/ml	Eluat auf Agar, Rekultivierung	> 90/> 90/> 90/> 90/> 90 d	Baumwolle/Baumwollfrottee/Mischgewebe aus Baumwolle und Polyester/Polyester/Polypropylen-Kunststoff	22,9-24,5 °C/30-49 %	Herkunft von Patienten oder aus der Krankenhausumgebung	[39]
Enterococcus spp.	7,2 lg KbE; Applikation von 20 µl	Vortexen 15 s in Wasser, Eluat auf Agar, Rekultivierung	mittlere Überlebensrate 3 d bei Antrocknung in Wasser, 43 d bei Antrocknung in Eiweiß	Glas	22 ± 2 °C/31 ± 3 %	Nachweisgrenze 2 KbE/KT	[26]
VRE	~ 6 lg KbE/KT	KT in dest. Wasser, US 60 Hz/20 min, Eluat auf Agar, Rekultivierung	nach 6 Wo Reduktion um ~ 3 lg	Stahl	21-27 °C/40-63 %	Lufttrocknung	[31]
	8 lg KbE/ml	5 min schütteln in 100 ml 0,9 %iger NaCl-Lsg. mit Glasperlen, Eluat auf Agar, Rekultivierung	1 bis 16 Wo	PVC	22 ±2 °C/50 ± 5 %	einige Enterokokkenstämme können unter trockenen Bedingungen > 4 Mon überleben; Bestimmung der Erregerzahl durch Membranfiltration und/oder Verdünnungsreihen	[42]
	E. faecalis: ~ 5 lg KbE/ml	Resuspension in Thioglycolatbouillon, Eluat auf Agar, Rekultivierung	22/22/22/> 80/> 80 d	Baumwolle/Baumwollfrottee/Mischgewebe aus Baumwolle und Polyester/Polyester/Polypropylen-Kunststoff	22,9-24,5 °C/30-49 %	Herkunft von Patienten oder aus der Krankenhausumgebung	[39]
	E. faecium: ~ 5 lg KbE/ml		> 90/> 90/> 90/> 90/> 90 d				

Micrococcus luteus	7,1-9,5 lg KbE/ml, Applikation von 20 µl	Filter in 0,89% NaCl 30 s vortexen (2 x im Abstand von 30 min), Eluat auf Agar, Rekultivierung	nach 120 d Abnahme um ~ 6 lg	Polycarbonatmembran-filter, Auswaschen der Übernachtkultur mit 0,89% NaCl	22 °C/31 %	Wiedergewinnungs-rate vom Membranfilter 5,4-7,8 lg KbE/ml; Überlebensrate ist aus der Abb. nur orien-tierend zu entnehmen	[29]
	5,2 lg KbE/ Baumwolltuch (2x2 cm)	in 4 ml PBS gemixt, Eluat auf Agar, Rekultivierung	nach 2 d Überleben von 20 % der KbE	Baumwolltuch	21 ± 1 °C /50 ± 5 %		[41]
Mycobacterium tuberculosis	(a) 0,001 und 1,0 Milli-gramm (mg)/ml<br>(b) 0,1 mg/ml<br>(c) 10 mg/ml<br>(d) 10 mg/ml<br><br>in Sputum und Wasser gelöst; Abstrich von 0,05 ml auf Deckgläsern (getrocknet)	Rekultivierung (k.A.), bei (c) und (d) zusätzlich Infektionsversuch (Tier)	(a) nach 4 d (= 40 Tageslicht-stunden) 1-4 KbE (Sputum), nach 12 d kein Wachstum in allen Proben<br>(b) bei Tageslicht rekultivierbar nach 1 d, bei Dunkelheit rekultivierbar für 9 d, nach 40 d nicht rekul-tivierbar<br>(c) bei Tageslicht für 51,5 h rekultivierbar (*in vitro*/Tier), nach 56,5 h nicht rekul-tivierbar (*in vitro*/Tier), bei	Deckgläser	(a) 27-4 °C/17-62 %, im Zimmer bei Tageslicht (sonnige Wintertage)<br>(b) 11-24 °C/35-94 %, im Zimmer bei Tageslicht versus (vs.) komplette Dunkelheit (bedecktes Frühjahrs-wetter)<br>(c) 16-31 °C/42-94 %, im Zimmer bei Tageslicht vs. komplette Dunkelheit	klinische Isolate, dosisabhängiges Wachstum (a)	[43]

			Dunkelheit nach 75 d nicht rekultivierbar (*in vitro* /Tier)<br>(d) bei Tageslicht nach 4 d nicht rekultivierbar *in vitro*, aber nach 5 d rekultivierbar im Tier (Versuche enden nach 5 d), bei Dunkelheit nach 200 d noch kultivierbar (Kultur/Tier), nach 424 d nicht rekultivierbar		(Sommer-wetter)<br>(d) 25 °C/13-87 %, im Zimmer bei Tageslicht (sonniges Winterwette r) vs. komplette Dunkelheit bei Kühlung (7 °C)		
Methicillin-sensitiver *Staphylococcus aureus* (MSSA)	7,3 lg KbE; Applikation von 20 µl	starkes Umrühren der Glasplättchen in 2 ml dest. Wasser für 15 s; Agar-basierte Methode zur Bestimmung überlebender Bakterien verwendet, wenn KbE < 300 KbE/ml	≥ 11 d	Objektträger aus Glas	20-24 °C/34 %		[26]

	5,2 lg KbE Baumwolltuch (2x2 cm)	in 4 ml PBS gemixt, Eluat auf Agar, Rekultivierung	nach 25 d Überleben von 0,8 % der KbE	Baumwolltuch	21 ± 1 °C/50 ± 5 %		[41]
	7,5 lg KbE/ml (1:1) mit Urin, Blut, Salzlsg. Wasser; Applikation von 20 µl	Resuspension in TSB, Eluat auf Agar, Rekultivierung	alle KT: Abnahme um ~ 1 lg KbE/ml nach 8 Wo	Keramikboden/Baumwoll-stofffragmente/synthetische Fasern/Eierkarton-Matratzenauflage	RT/k.A.	KT 3 cm^2	[40]
	5-6 lg KbE in 2 ml sterilem Speichel	Vortexen in 2 ml PBS (30 s), Eluat auf Agar, Rekultivierung	bis 2 d	Schilf	25 °C	zudem wurde Klarinettenspiel simuliert (bis zu 5 d kultivierbar)	[44]
	8 lg KbE/ml; Applikation von 10 µl	KT 1min vortexen; Eluat auf Agar	2 d/18 d/> 45 d/43 d	Latex/Baumwolle/ Vinylbelag/Granit	RT/k.A.	Teststamm ATCC 25923; KT 10x20 mm	[45]
	~ 6,5 lg KbE/ Testfläche	Testfläche auf Agar, Rekultivierung	37 d/37 d/41 d/37 d (qualitativ)	Baumwolle/Baumwolle-Polyester/Wolle/Seide	21-24 °C/30-49 %	klinische Isolate, Krankenhaustextilien	[35]
	6 lg KbE/ml; Applikation von 0,25 ml auf KT	mit in steriler NaCl-Lsg. angefeuchtetem Tupfer beprobt, auf Agar überimpft, Rekultivierung	9 d/10 d/3 d	Formica/Edelstahl/Infusionsständer (Emaille)	20-22 °C, 60-70 %	KT 8 cm^2	[46]
	~ 8,4 lg KbE; Applikation von 0,25 µl/25 cm^2	Kontaktkultur von kontaminierter Fläche, Rekultivierung	~ 28 KbE nach 14 d, 5 KbE nach 21 d/~ 6 KbE nach 7 d, /~ 4 KbE nach 21 d /~ 25 KbE nach 3 d, ~ 10 KbE nach 7 d	Glas/PVC/Stahl/Aluminium, angetrocknet in 0,89 % NaCl-Lösung	21°C/31-35 %	Teststamm ATCC 25932; Kalkulation der Überlebenszeit bezogen auf 250 KbE in der Kontaktkultur als zählbare Ausgangs-KbE	[36]
	7,2 lg KbE, Applikation von 20 µl	Vortexen 15 s in Wasser; Eluat auf Agar, Rekultivierung	mittlere Überlebensrate 26 d bei Antrocknung	Glas	22 ± 2 °C/31 ± 3 %	Nachweisgrenze 2 KbE/KT	[26]

			in Wasser, 35 d bei Antrocknung in Eiweiß; nach 12 d ~ 3 lg Verlust, nach 18 d ~ 5,7 lg Verlust				
	für Austrocknungs-versuch: 7,3 lg KbE/cm², Applikation von 50 µl;<br>für Feuchtig-keitsversuch: 3-4 lg KbE/ml	bei Austrocknungs-versuch: Alufolie in Bouillon ausge-schüttelt, Eluat auf Agar, Rekultivierung;<br>bei Feuchtigkeits-untersuchungen: Ausspateln der Wasser-Bakterien-Suspensionen auf Agar, Rekultivierung	4,4 lg KbE/cm² nach 25 d Austrocknung<br>in Leitungswasser nach 7 d/in Aqua bidest nach 5 d nicht mehr kultivierbar	Austrocknungsversuche auf Alufolie<br>Untersuchungen in feuchtem Milieu in Aqua bidest/sterilem Leitungswasser	RT und 40-50 %<br>RT/k.A.; bei Leitungswasser auch Kaltvernebelung bei 30-40 °C	klinische Isolate	[47]
	6-7 lg KbE/ml	Staubprobe auf Agar, Kultivierung	bei Trockenheit bis zu > 7 Mon, bei 32 % RLF > 5 Mon, bei 77 % RLF 1,5 Mon	Hausstaub	RT/0 %, 32 %, 42 %, 58 %, 77 %, 99 %	klinische Isolate, je höher die Feuchtigkeit, desto häufiger traten Verunreinigungen durch Schimmelsporen auf	[48]
	Probenkörper (1 cm²) bestrichen mit a) Trocken-inokulum: 5-6 lg/cm² oder b) Flüssig-inokulum: ~ 6	Einlegen Proben-körper in TSB (silberhaltige Proben mit 5% Pferdeserum neutralisiert), Aufbringen auf Agar, Rekultivierung	a) nach 24 h 6,7 lg/cm², nach 7 d 22 KbE/cm² ohne Silber;<br>nach 24 h 6,3 lg/cm², nach 7 d 1 KbE/cm²; b) nach 7 d 16,2 lg/cm² ohne	Polymer ohne und mit Silberimprägnierung	37 °C/feuchte Kammer	Teststamm ATCC 6538, z.T. schwache Hemmung durch Silber	[49]

	lg KbE/ml; Applikation von 50 µl		Silber, 6,1 lg KbE/ml mit Silber				
	8 lg KbE/ml	Austrocknung in Flaschen; Zurückgewinnung mit 1 ml PBS, vortexen, Eluat auf Agar, Rekultivierung	mit Staub: bis 28 d; ohne Staub: bis 35 d	Material der Flaschen nicht berichtet (n.b.) /mit und ohne Hausstaub	22-27 °C/27-45 %	MSSA und Methicillin-resistenter *Staphylococcus aureus* (MRSA) hatten in Hausstaub (aus dem Krankenhaus) kürzere Tenazität	[50]
Epidemischer Methicillin-resistenter *Staphylococcus aureus (EMRSA)*	8,7 lg KbE/ml	30 s in 10 ml PBS vortexen, 50 ml Eluat auf Agar, Rekultivierung	≤ 60 min, 270 min, mind. 360 min	Kupfer, Messing (80 % Cu, 20 % Zn), Edelstahl	4 °C und 22 °C und 50 ±10 %	Epifluoreszenz-mikroskopie-Analyse wurde auch verwendet, MRSA persistierte bis 72 h auf Edelstahl, aber nicht auf Kupfer	[51]
Staphylococcus aureus (MRSA)	6-7 lg KbE/KT	KT in dest. Wasser, US 60 Hz/20 min Testplättchen (Ultraschall), Eluat auf Agar, Rekultivierung	nach 6 Wo Reduktion um 5-6 lg	Stahl	21-27 °C/40-63 %	Lufttrocknung	[31]
	8 lg KbE/ml, Applikation von 10 µl	KT vortexen1 min, Eluat auf Agar, Rekultivierung	1 d/18 d/41 d/40 d	Latex/Baumwolle/ Vinylbelag/Fliese	RT/k.A.	Teststamm ATCC 33591; KT 10x20 mm	[45]
	3,2-4,9 lg KbE/100 cm^2	Ausschütteln für 30 min, anschließend 5 min US, Eluat auf Agar, Rekultivierung	Wiederfindung 59-125 % nach 7 d, 26-42 % nach 14 d, 0,2-16 % nach 28 d, 0-1 % nach 56 d	Trockene Mops, Einsatz in 4 Patientenzimmern	4-28 °C/50-75 %		[6]

	9 lg KbE/ml	Suspension mit/ohne Staub war nach 10 d verdunstet; restliche Bakterien mit 1 ml PBS gewonnen, vortexen, Eluat auf Agar, Rekultivierung	bis 318 d	Kunststoff mit und ohne Zusatz von Staub	20-22 °C/24-47 %	Staub hat keine Auswirkung auf Persistenz	[52]
	8 lg KbE/ml	Trocknung in Flaschen; Zurückgewinnung durch 1 ml PBS, vortexen, Eluat auf Agar, Rekultivierung	mit Staub: bis 126 d; ohne Staub: bis zu 175 d	Material der Flaschen nicht berichtet (n.b.)/mit und ohne Hausstaub	22-27 °C/27-45 %	MRSA und MSSA hatten in Hausstaub (aus dem Kranken-haus) kürzere Tenazität	[50]
	5,6 lg KbE; Applikation von 10 µl	Thioglycolat Medium, Eluat auf Agar, Rekultivierung	bis zu 21/14/3/40/> 51 d	Baumwolle/Baumwoll-frottee/Mischgewebe aus Baumwolle und Polyester/Polyester/Polypropylen-Kunststoff	22,9-24,5 °C/30-49 %	KT 8 cm^2	[39]
	~ 7,3 lg KbE/Applikation von 20 µl PBS/dest. Wasser auf Glas unter Aus-trocknung	15 s vortexen in Wasser, Eluat auf Agar, Rekultivierung	bis zu 96 d	Glas	RT/31 %	Vorhandensein von gesättigtem Kalziumchlorid ($CaCl_2$) × 6 H_2O während der Lagerung	[53]
VISA	8 lg KbE/ml; Applikation von 10 µl	KT 1 min vortexen, Eluat auf Agar, Rekultivierung	1/3/> 45/> 45 d	Latex/Baumwolle/ Vinylbelag/Granit	RT/k.A.	KT 10x20 mm	[45]
Streptococcus equi	8,3 lg KbE/ml	Baumwolltupfer PBS befeuchtet und gründlich über die zu beprobende Stelle gewischt,	bis zu 3 d	Holz (jeweils unbehandelt und lackiert), Metall und Gummi	k.A.		[54]

		Überimpfung auf Agar, Rekultivierung					
	8 lg KbE/ml	Abstrich mittels Baumwolltupfer, Ausstrich auf Blutagar, Rekultivierung	im Sommer: bis7 d auf Kunststoff; im Winter: bis 35 d auf Kunststoff	Holz/Schuhsohle, Baumwoll-Anzüge/Nasensonden-schlauch/Zahn-Raspel (Stahl)/feuchter Kunststoffeimer (alle innerhalb einer Holzhütte)/Zaunpfahl (im Baumschatten)	Sommer: ø 18,8 °C/70 % Winter: ø 5,2 °C/87 %	unter Winterbedingungen längeres Überleben	[55]
Streptococcus faecalis	für Trocknungs-versuch: 6,9 lg KbE/cm², Applikation von 50 µl;<br><br>für Feuchtig-keitsversuch: 3-4 lg KbE/ml	Für Trocknungs-versuch: Alufolie in Bouillon ausge-schüttelt, Eluat auf Agar, Rekultivierung<br><br>bei Feuchtigkeits-untersuchungen: Eluat auf Agar, Rekultivierung	4,6 lg KbE/cm² nach 25 d Austrocknung<br><br>in Leitungswasser nach 10 d/in Aqua bidest nach 11 d nicht mehr kultivierbar		RT und 40-50 %<br><br>RT und bei Leitungswasser auch Kaltvernebelung bei 30-40 °C	klinische Isolate	[47]
Streptococcus pyogenes	5-6 lg KbE in 2 ml sterilem Speichel	Vortexen in 2 ml PBS (30 s), Eluat auf Agar, Rekultivierung	bis 24 h	Schilf	25 °C	zudem wurde Klarinettenspiel simuliert (bis zu 48 h kultivierbar)	[44]
	~ 7,7 lg KbE/ml; Applikation von 100 µl (Lebensmittel) bzw. 10 µl	Eluat auf Agar, Rekultivierung	Überleben bei 5 °C auf Lebensmitteln bis zu 24 h; übrige Oberflächen: < 2 h bei 21 °C	Kunststoff- und Keramikteller/Kunststoff-becher/Edelstahl/Lebens-mittel	21 °C bzw. 5 °C bei Lebensmitteln/ k.A.	Detektionsgrenze 2 lg KbE	[56]

	8 lg KbE/ml; Austrocknung	Resuspension der ausgetrockneten Oberflächen und Anzucht auf Agar bzw. Infektionsversuche im Tier	auf Oberflächen — planktonisch: 3 d, als Biofilm: > 120 d; Hand: planktonisch etwa 3 min, als Biofilm längeres Überleben; Biofilme nach 1 Mon Antrocknung sind nach Resuspension und intranasaler Einbringung in Tier rekultivierbar	Oberflächen (Kunststoff Bücher, Stofftiere, Spielzeug)/geripptes Leinen/ Haut (Hände)	RT/k.A., Lagerung im Dunkeln	im Biofilm unempfindlicher gegen Austrocknung	[57]
Streptococcus pneumoniae	5-6 lg KbE in 2 ml sterilem Speichel	Vortexen in 2 ml PBS (30 s), Eluat auf Agar, Rekultivierung	< 24 h	Schilf	25 °C	Klarinettenspiel wurde nicht für *Str. eptococcus pneumoniae* simuliert	[44]
Speichel-Streptokokken, -Staphylokokken	5,3 lg KbE/ml für *Staphylococcus aureus* Stamm C55; 5,9 lg/ml KbE/ml für *Streptococcus pyogenes* FF22 und 5,8 lg KbE/ml für *Streptococcus salivarius* Stamm Min 5; Applikation von	Kontaktkultur auf Agar	> 88 h	Glas/Speichel, Glas/Serum, Latex/Speichel, Latex/Serum, Holz/Serum	~ 25 °C in direktem Sonnenlicht/ k.A.	auch Testung auf Kunststoff und Edelstahl (ähnlich langes Überleben wie auf Glas); Teststämme C55 und FF22 überlebten länger, wenn Speichel zugesetzt wurde	[58]

	100 µl; alle resistent gegen 1 mg/ml Streptomycin						
**Gram-negativ**							
Acinetobacter baumannii	~ 6,5 lg KbE/Testfläche	Testfläche auf Agar, Kultivierung	19 d/19 d/7 d/19 d (qualitativ)	Baumwolle/Baumwolle-Polyester/Wolle /Seide	21-24 °C/30-49 %	klinische Isolate, Krankenhaustextilien	[35]
	6-7 lg KbE/KT	KT in dest. Wasser, US 60 Hz/20 min, Eluat auf Agar, Rekultivierung	nach 6 Wo Reduktion um 4-5 lg	Stahl	21-27 °C/40-63 %	Teststamm NCTC 12156; Lufttrocknung	[31]
	6 lg KbE/ml; Applikation von 0,25 ml auf KT	mit in steriler NaCl-Lsg. angefeuchtetem Tupfer beprobt, auf Agar überimpft, Rekultivierung	11 d/12 d/6 d	Formica/Edelstahl/Infusionsständer (Emaille)	20-22 °C, 60-70 %	KT 8 cm^2	[46]
	~ 8,4 lg KbE; Applikation von 0,25 µl/25 cm^2	Kontaktkultur von kontaminierter Fläche, Kultivierung	250 KbE nach 7 d, ~ 112 KbE nach 28 d/250 KbE nach 7 d, ~ 180 KbE nach 21 d, ~ 12 KbE nach 28 d/250 KbE nach 7 d, 150 KbE nach 14 d, 112 KbE nach 28 d/250 KbE nach 7 d, ~ 125 KbE nach 14 d, ~ 18 KbE nach 28 d	Glas/PVC/Stahl/Aluminium, angetrocknet in 0,89 % NaCl-Lsg.	21 °C/31-35 %	Teststamm DSM 30011, Kalkulation der Überlebenszeit bezogen auf 250 KbE in der Kontaktkultur als zählbare Ausgangs-KbE	[36]
	7,1-9,5 lg KbE/ml, Applikation von 20 µl	Filter in 0,89 % NaCl 30 s vortexen (2 x im Abstand von 30 min), Eluat auf Agar, Rekultivierung	Nach 5 d Abnahme um etwa 2,5 lg, nach 20 d um etwa 5,5 lg	Polycarbonatmembranfilter, Auswaschen der Übernachtkultur mit 0,89 % NaCl vor Applikation auf das Filter	22 °C/31 %	ATCC 19606^T; Wiedergewinnungsrate vom Membranfilter 5,4-7,8 lg KbE/ml;	[29]

						Überlebensrate ist aus der Abb. nur orientierend zu entnehmen	
	n.b.	15 s in 2 ml dest. Wasser geschüttelt, 100 µl Aliquots auf Agar, Rekultivierung	Biofilmbildende bis 36 d/nicht biofilmbildende bis 15 d	Glas	n.b./31 ± 3 %		[59]
	7,3 lg KbE; Applikation von 20 µl	Vortexen in 2ml dest. Wasser (15 s), Agar-Inkorporations-methode, wenn Konzentration auf < 300 KbE/ml sank	20 d/3 d	Glas	RT/31 %<br>RT/10 %	Glasscheibe (Ø 13mm)	[60]
	7,3 lg KbE/20 µl dest. Wasser auf Glas unter Austrocknung		21-33 d	Glas	22 ±3 °C/31 %		[61]
	7,3 KbE; Applikation von 20 µl PBS/dest. Wasser auf Glas unter Trocknung	Resuspension, Eluat auf Agar, Rekultivierung	bis 70 d	Glas	RT/31 %	Teststämme ATCC 17978 und CC2, ACICU	[62]
	~ 8 lg KbE/ml; Applikation von 5 µl Suspension unter Trocknung	Resuspension, Eluat auf Agar, Rekultivierung	max. Überlebens-zeit für ATCC 19606: 3 d; A118: 20; ATCC 17904: 22 d; ATCC 17978: 34 d; AB09-002: 43 d; AB09-003: > 90 d; ATCC 17961: > 90 d; AB5075: ~ 90 d	Kunststoff (Polystyrol)	19-23 ˚C/25-61 %		[63]
	~ 7,3 lg KbE; Applikation von 20 µl PBS/dest.	Vortexen in Wasser, Eluat auf Agar, Rekultivierung	bis 96 d	Glas	RT/31 %	Vorhandensein von gesättigtem $CaCl_2 \times 6$	[53]

	Wasser auf Glas unter Trocknung					H_2O während der Lagerung	
	8 lg KbE	5 min in 100 ml NaCl vortexen auf Glasperlen, Membranfiltration; Rekultivierung auf Agar	50 % der Stämme mittlere Überlebensdauer mind. 2 Wo (< 2lg rekultivierbar); stammabhängig bis 4 Mon (7 lg rekultivierbar)	Keramik/PVC/Gummi/Stahl (5 x 5 cm); Isolate aus Abwasser und Urin empfindlicher als von trockenen Flächen	22 ± 2 °C/50 ± 5 %	Wiedergewinnungs-rate vom Membranfilter 12-96 % (im Mittel 46 %)	[38]
	7,2 lg KbE, Applikation von 20 µl	Vortexen 15 s in Wasser; Eluat auf Wasser, Eluat auf Agar, Rekultivierung	mittlere Überlebensrate stammabhängig 2-29 d bei Antrocknung in Wasser, bis 59 d bei Antrocknung in Eiweiß; nach 12 d ~ 1 lg Verlust, nach 18 d ~ 5,5 lg Verlust	Glas	22 ± 2 °C/31 ± 3 %	Nachweisgrenze 2 KbE/KT	[26]
Acinetobacter johnsonii			mittlere Überlebensrate 3 d bei Antrocknung in Wasser, 12 d bei Antrocknung in Eiweiß				
Acinetobacter junii			mittlere Überlebensrate 2 d bei Antrocknung in Wasser, 13 d bei Antrocknung in Eiweiß				

Acinetobacter lwoffi			mittlere Überlebensrate 6 d bei Antrocknung in Wasser, 8 d bei Antrocknung in Eiweiß				
	7,3 lg KbE; Applikation von 20 µl	Vortexen in 2 ml dest. Wasser (15 s), Agar-Inkorporations-Methode, wenn Konzentration auf < 300 KbE/ml sank	3 d/18 h	Glas	RT/31 %<br>RT/10 %	Glasscheibe Ø 13mm	[60]
Acinobacter calcoaceticus anitratus	8 lg KbE/ml; Applikation von 0,02 ml (= 4 lg KbE/Probe) auf Finger bzw. Hartfaserplatte	Abschwemmung in Ringerlösung mit Glasperlen sowie Mischung mit MacConkey Bouillon, Kultivierung	Abnahme um 2-2,5 lg KbE/Finger nach 1 h/ Hartfaserplatte: Abnahme um 1 lg KbE nach 1 h	Finger/Hartfaserplatten	RT/n. b.	klinische Isolate	[64]
	5,2 lg KbE/Baumwoll-tuch (2x2 cm)	in 4 ml PBS gemixt, Eluat auf Agar, Rekultivierung	nach 25 d Überleben von 0,6 % der KbE/nach 7 h Überleben von 40 % der KbE	Baumwolltuch/Glas	21 ± 1 °C /50 ± 5 %		[41]
Acinetobacter calcoaceticus lwoffii	4 lg KbE/Finger bzw. Hartfaserplatte (Formica)	Abschwemmung in Ringerlösung mit Glasperlen sowie Mischung mit MacConkey Bouillon, Kultivierung	Abnahme um 2-2,5 lg KbE/Finger nach 1 h/ Hartfaserplatte: Abnahme um 1 lg KbE nach 1 h	Finger/Hartfaserplatten	RT/n. b.	klinische Isolate	[64]
	5,2 lg KbE/ Baumwolltuch (2 x 2 cm)	in 4 ml PBS gemixt, Eluat auf Agar, Rekultivierung	nach 7 d nicht mehr kultivierbar	Baumwolltuch	21 ± 1 °C /50 ± 5 %		[41]

Acinetobacter radioresistens	7,3 lg KbE; Applikation von 20 µl	Vortexen in 2 ml dest. Wasser (15 s), Agar-Inkorporations-Methode, wenn Konzentration auf < 300 KbE/ml sank	157 d /31 d	Glas	RT/31 %<br>RT/10 %	Glasscheibe Ø 13 mm; Überstehen Austrocknung besser als andere *Acinetobacter* spp., Erhöhung der RLF auf 31 % = durch-schnittliche Ver-längerung der Über-lebenszeit um 126 d	[60]
Campylobacter jejuni	0,1 ml kontaminiertes Wasser aus Schraubkühlern	Kontaktagarplatte sowie Eintauchen 20 ml in Bouillon (2 min schütteln), Eluat auf Agar, Rekultivierung	schnelle Trocknung: alle KT 0,25 h; langsame Trocknung: 4 h Aluminium u. Edelstahl; 7 h Laminat u. Keramik	Aluminium/Edelstahl/ Formica/Keramik	20-24 °C/n. b.	KT 4x4 cm, nur rekultivierbar, solange Fläche feucht	[65]
	8-9 lg KbE/ml	Eluat auf Agar, Rekultivierung	in belüfteter Bouillon<br>- bei 30 °C mit unversiegeltem Holz ~ 5 lg KbE/ml nach 28 d (ohne Holz 0 lg KbE/ml nach 2 d)<br>- bei 4 °C mit unversiegeltem Holz ~ 2 lg KbE/ml nach 25 d (ohne Holz ~ 2 lg KbE/ml nach 20 d)	Holz/Polyurethanschaum (Poren 320-550 Mikrometer (µm)/Glas (Poren 10-250 µm)/Kunststoffschneide-brett	4, 25, 30 °C/k.A.	klinische Isolate (UOS 2843); unversiegeltes Holz bzw. andere Oberflächen mit Poren ~ 16 µm begünstigen unter belüfteten Be-dingungen Überleben (besonders bei hohen Temperaturen)	[66]

			(Überleben > 2 d nur auf ~ 16 µm porigem Glas)				
Enterobacter cloacae	~ 8,4 lg KbE; Applikation von 0,25 µl/25 cm^2	Kontaktkultur von kontaminierter Fläche, Kultivierung	~ 14 KbE nach 3 d /~ 8 KbE nach 2 d /~ 12 KbE nach 3 d /~ 6 KbE nach 2 d	Glas/PVC/Stahl/Aluminium, angetrocknet in 0,89 % NaCl-Lsg.	21 °C/31-35 %	Teststamm ATCC13047; Kalkulation der Überlebenszeit bezogen auf 250 KbE in der Kontaktkultur als zählbare Ausgangs-KbE	[36]
Escherichia coli	5-6 lg KbE/in 2 ml sterilem Speichel	Vortexen in 2 ml PBS (30 s), Inkubation bei 35 °C 5 % CO_2 für 48 h, Eluat auf Agar, Rekultivierung	bis 2 d	Schilf	25 °C/k.A.	zudem wurde Klarinettenspiel simuliert (bis zu 72 h kultivierbar)	[44]
	6 lg KbE/cm^2	mit 0,1 % Peptonwasser angefeuchteter Wattetupfer, in 10 ml Maximum Recovery Diluent, Suspension auf Agar, Rekultivierung	nach 48 h ~ 1,5 lg KbE/cm^2 (Kunststoff)<br><br>nach 24 h ~ 1,5 lg KbE/cm^2 (Karton)	Kunststoff/Karton	RT/k.A.	Verpackungsmaterial	[67]
	9 lg KbE/100 µl	Resuspension, Eluat auf Agar, Rekultivierung	bei 5 °C nach 300 d Reduktion um 2-3 lg; bei 25 °C nach 100 d Reduktion um 8 lg	Kunststoff und Lebensmittel	5, 15, 25 °C/ 22, 43, 58, 68, 93 %		[68]
	8 lg KbE/ml Rinderkot,	dreimaliges Abschwemmen in	bei 5 °C, trocken, nach 28 d nicht	Holz/Stahl	5 und 20 °C unter trockenen		[69]

	Aufbringen von 500 µl Kot auf Proben (7,3 lg KbE/cm^2)	Ringerlösung; Eluat auf Agar, Rekultivierung	mehr kultivierbar; bei 20 °C, trocken nach max. 7 d nicht mehr kultivierbar /bei Feuchtigkeit > 28 d		und feuchten Bedingungen (Befeuchtung mittels künstlichem Regenwasser)		
	7-8 lg KbE/ml	Eintauchen der Oberfläche für 2 min in 5 ml Nährbouillon; Eluat auf Agar, Rekultivierung	bis zu 120 min	Kunststoff/Holz	RT/k.A.		[70]
	5,2 lg KbE/ Baumwolltuch (2x2 cm)	in 4 ml PBS gemixt, Eluat auf Agar, Rekultivierung	nach 7 h nicht mehr kultivierbar/nach 7 h Überleben von 0,8 % der KbE	Baumwolltuch/Glas	21 ± 1 °C /50 ± 5 %	überlebte länger mit 2 % Rinderalbumin oder 5 % Pferdeserum	[41]
	7,5 lg KbE/ml (1:1) mit Urin, Blut, Salzlsg. Wasser; Applikation von 20 µl	Resuspension in TSB, Eluat auf Agar, Rekultivierung	alle KT: Abnahme um ~ 1 lg KbE/ml nach 8 Wo	Keramikboden/Baumwoll-stofffragmente/synthe-tische Fasern/Eierkarton-Matratzenauflage	RT/k.A.	KT 3 cm^2	[40]
	7-9 lg KbE/ml	eluiert in 99 ml PBS; Gussplattentechnik	Abnahme um 1,7 lg nach 2 h/ Abnahme um 0,37 4 lg nach 2 h/ Abnahme um 1,09 1 lg nach 2 h/ Abnahme um 0,44 lg nach 2 h/	Schneidebretter aus Holz und Kunststoff neues, trockenes Holzbrett/neues, feuchtes Holzbrett/benutztes, trockenes Holzbrett/benutztes, feuchtes Holzbrett/Kunststoffbrett	k.A.		[71]

Attachment to: Commission for Hospital Hygiene and Infection Prevention (KRINKO). Hygiene requirements for cleaning and disinfection of surfaces: recommendation of the Commission for Hospital Hygiene and Infection Prevention (KRINKO) at the Robert Koch Institute. GMS Hyg Infect Control. 2023;18:Doc13. DOI: 10.3205/dgkh000468

			Abnahme um 0,06 1 lg nach 24 h				
	8 lg KbE/ml	5 min in 100 ml NaCl vortexen auf Glasperlen, Membranfiltration, Eluat auf Agar, Rekultivierung	bis 4 Mon (~ 2lg rekultivierbar)	Keramik/PVC/Gummi/Stahl (5 x 5 cm)	22 ± 2 °C/50 ± 5 %	Wiedergewinnungs-rate vom Membranfilter 12-96 % (im Mittel 46 %), ATCC 11775	[38]
	~ 6,5 lg KbE/Testfläche	Testfläche auf Agar, Kultivierung	45 d/37 d/45 d/45 d (qualitativ)	Baumwolle/Baumwolle-Polyester/Wolle/Seide	21-24 °C/30-49 %	klinische Isolate, Krankenhaustextilien	[35]
	~ 3-7 lg KbE/cm^2	mit 0,1 % Peptonwasser angefeuchteter Baumwolltupfer, in 10 ml Maximum Recovery Diluent, Suspension auf Agar, Rekultivierung	bei Ausgangs-kontamination 3,5 lg KbE/cm^2 nach 8 h kein Nachweis; bei 4,6 lg nach 8 h Wiederfindung ~ 1,3 lg; bei 6,5 lg Wiederfindung nach 8 h ~ 3,8 lg, nach 24 h ~ 2,3 lg und nach 48 h ~ 1,5 lg	Kunststoff	25 °C	Verpackungsmaterial	[67]
	~ 8,4 lg KbE; Applikation von 0,25 µl/25 cm^2	Kontaktkultur von kontaminierter Fläche, Kultivierung	~ 6 KbE nach 1 d/1 KbE nach 7 d/1 KbE nach 1 d/1 KbE nach 3 d/10 KbE nach1 d/1 KbE nach 5 d	Glas/PVC/Stahl/Aluminium, jeweils angetrocknet in 0,89 % NaCl-Lsg.	21 °C/31-35 %	Teststamm ATCC25922; Kalkulation der Überlebenszeit bezogen auf 250 KbE in der Kontaktkultur als zählbare Ausgangs-KbE	[36]
	7,1-9,5 lg KbE/ml,	Filter in 0,89 % NaCl 30 s vortexen (2 x im Abstand von 30 min),	nach 6 h Abnahme um etwa 6,5 lg	Polycarbonatmembranfilter	22 °C/31 %	Stamm DH5α; Wiedergewinnungs-rate vom	[29]

	Applikation von 20 µl	Eluat auf Agar, Rekultivierung				Membranfilter 5,4-7,8 lg KbE/ml	
	7,2 lg KbE, Applikation von 20 µl	Vortexen 15 s in Wasser	mittlere Überlebensrate 1 d bei Antrocknung in Wasser, 3 d bei Antrocknung in Eiweiß	Glas	22 ± 2 °C/31 ± 3 %	Nachweisgrenze 2 KbE/KT	[26]
	6-7 lg KbE/ml	Direktausstrich auf Agar, Kultivierung	bei 0/32/58 % > 8 Mon/bei 77 % > 5 Mon	Hausstaub	RT/0, 32, 42, 58, 77, 99 %	klinische Isolate, je höher die Feuchtigkeit, desto häufiger traten Verunreinigungen durch Schimmelsporen auf	[48]
	für Trocknungs-versuch: 6,9 lg KbE/cm^2, Applikation von 50 µl<br><br>für Feuchtig-keitsversuch: 3-4 lg KbE/ml	bei Trocknungs-versuch: Alufolie in Bouillon ausgeschüttelt, Eluat auf Agar, Rekultivierung;<br>bei Feuchtigkeits-untersuchungen: Ausspateln der Wasser-Bakterien-Suspensionen auf Agar, Rekultivierung	0,7 lg KbE/cm^2 nach 25 d Austrocknung<br><br>überlebt in Leitungswasser > 12 d, stirbt in Aqua bidest nach 20 d ab	Austrocknungsversuche auf Alufolie<br><br>Untersuchungen in feuchtem Milieu in Aqua bidest/sterilem Leitungswasser	RT und 40-50 %<br><br>RT/k.A.; bei Leitungswasser auch Kaltvernebelung bei 30-40 °C	klinische Isolate	[47]
	Probenkörper (1 cm^2) bestrichen mit Trocken-inokulum: 5-6 lg/cm^2	Einlegen Probenkörper in TSB (silberhaltige Proben mit 5 % Pferdeserum neutralisiert),	nach 24 h 0,2 KbE/cm^2, nach 7 d kein Nachweis ohne Silber; nach 24 h 132 KbE/cm^2,	Polymer ohne und mit Silberimprägnierung	37 °C/feuchte Kammer	Teststamm ATCC 11229	[49]

		Aufbringen auf Agar, Rekultivierung	nach 7 d 8 KbE/cm^2 mit Silber				
Francisella tularensis	~ 8 lg KbE/KT	Aliquot (0-1 ml) einer 10-fachen Verdünnung in PBS, Eluat auf Agar, Rekultivierung	nach 240 h 4 lg KbE auf Glas bei RT und 38 °C, nach 96 h nicht mehr kultivierbar auf Papier bei RT und 38 °C	Glas und Papier	22-60 °C und 30-75 %	höhere Temperaturen oder höhere RLF führten zu schnellem starken Abfall	[72]
Helicobacter (H.) pylori	9 lg KbE/ml	Suspension von *H. pylori* in sterilisiertem Wasser (Auftauwasser von Geflügel)	nach ~ 15 min 3,2 lg bzw. nach 60 min 1 lg/nach ~ 180 min ~ 1,9 lg bzw. nach 240 min ~ 2 lg	Holz bzw. Kunststoff/Keramik	RT/5-7 °C	Nachweis nur so lange, wie Fläche feucht	[73]
Klebsiella pneumoniae	5,2 lg KbE/Baumwoll-tuch (2 x 2 cm)	in 4 ml sterile PBS gemixt, Eluat auf Agar, Rekultivierung	nach 1 h nicht mehr kultivierbar	Baumwolltuch	21 ±1 °C /50 ±5 %		[41]
	7,5 lg KbE/ml (1:1) mit Urin, Blut, Salzlsg. Wasser; Applikation von 20 µl	Resuspension in TSB, Eluat auf Agar, Rekultivierung	alle KT: Abnahme um ~ 1 lg KbE/ml nach 8 Wo	Keramikboden/Baumwoll-stofffragmente/synthe-tische Fasern/Eierkarton-Matratzenauflage	RT/k.A.	KT 3 cm^2	[40]
	~ 6 lg KbE/KT	KT in dest. Wasser, US 60 Hz/20 min, Eluat auf Agar, Rekultivierung	nach 6 Wo Reduktion um ~ 5 lg	Stahl	21-27 °C/40-63 %	Lufttrocknung	[31]
	~ 8,4 lg KbE; Applikation von 0,25 µl/25 cm^2	Kontaktkultur von kontaminierter Fläche, Kultivierung	~ 26 KbE nach 3 d, 1 KbE nach 7 d /50 KbE nach 2 d, 10 KbE nach 3 d/nicht	Glas/PVC/Stahl/Aluminium, jeweils angetrocknet in 0,89 % NaCl-Lsg.	21 °C/31-35%	Teststamm ATCC700603, Kalkulation der Überlebenszeit bezogen auf 250 KbE	[36]

			ablesbar /~ 10 KbE nach 2 d			in der Kontaktkultur als zählbare Ausgangs-KbE	
	für Trocknungs-versuch: 7 lg KbE/cm², Applikation von 50 µl	bei Trocknungs-versuch: Alufolie in Bouillon ausgeschüttelt, Eluat auf Agar, Rekultivierung	1,8 lg KbE/cm² nach 25 d Austrocknung	Trocknungsversuche auf Alufolie	RT und 40-50 %	klinische Isolate	[47]
	6-7 lg KbE/ml	Direktausstrich auf Agar, Kultivierung	bei 0/32/58 % > 15 Mon/bei 99 % > 16 Mon	Hausstaub	RT/0, 32, 42, 58, 77, 99 %	klinische Isolate, je höher die Feuchtigkeit, desto häufiger traten Ver-unreinigungen durch Schimmelsporen auf	[48]
Listeria monocytogenes	~ 3-7 lg KbE/cm²	angefeuchteter Baumwolltupfer, überführt in Nährmedium, in 10 ml Maximum Recovery Diluent, Suspension auf Agar, Rekultivierung	bei Ausgangs-kontamination 3,8 lg nach 8 h kein Nachweis; bei 6,8 lg Wiederfindung nach 48 h bei ~ 3,4 lg KbE/cm²	Kunststoff	25 °C/k.A.	Verpackungsmaterial	[67]
	7-8 lg KbE/ml	2 min Schütteln in 5 ml Nährbouillon, Eluat auf Agar, Rekultivierung	4 lg KbE nach 180 min	Holz/Kunststoff	RT/k.A.		[70]
	6 lg KbE/ml, Applikation von 25 µl	Resuspension (abgekratzt und abgeschwemmt), Eluat auf Agar, Rekultivierung	Edelstahl: (a) Abnahme nach 10 d um 1 lg KbE/cm²; (b) Abnahme nach 10 d um 4,5 lg	rostfreier Edelstahl/Acrylnitril-Butadien-Kautschuk (ABK)	25 °C/32,5 % (a) 25 °C/75,5 % (b) 6 °C/32,5 % (c) 6 °C/75,5 % (d)	Teststamm ScottA	[74]

			KbE/cm^2; (c) Abnahme nach 10 d um 1,5 lg KbE/cm^2; (d) Abnahme nach 10 d um 4 lg KbE/cm^2/ ABK: (a) Abnahme nach 5 d um 4,5 lg KbE/cm^2; (b) Abnahme nach 10 d um 4,5 lg KbE/cm^2; (c) Abnahme nach 10 d um 3 lg KbE/cm^2; (d) Abnahme nach 10 d um 1 lg KbE/cm^2				
	9 lg KbE/ml, Applikation von 25 µl	US-Bad, serielle Verdünnungen in Pepton-Salzlösung, Eluat auf Agar, Rekultivierung	ohne Biofilm, osmoadaptiert, 0,5 % NaCl: Abnahme um 1,5 lg KbE/cm^2 nach 20 d;<br>ohne Biofilm, osmoadaptiert, 5 % NaCl: Abnahme um 0,75 lg KbE/cm^2 nach 25 d;<br>mit Biofilm, osmoadaptiert, 0,5 % NaCl: Abnahme um 1,25	rostfreier Edelstahl	15 °C/43 %	in den ersten d schneller Abfall, danach langsam	[75]

			lg KbE/cm^2 nach 50 d; mit Biofilm, nicht osmoadaptiert, 0,5 % NaCl: Abnahme um 2,25 lg KbE/cm^2 nach 50 d;<br>mit Biofilm, osmoadaptiert,<br>5 % NaCl: Abnahme um 1 lg KbE/cm^2 nach 50 d; mit Biofilm, nicht osmoadaptiert,<br>5 % NaCl: Abnahme um 1,5 lg KbE/cm^2 nach 50 d				
	8 lg KbE/cm^2	US-Bad, 10-fache serielle Verdünnungen in Physiologischer Pepton-Salz-Lösung (PPS), Eluat auf Agar, Rekultivierung	(a) in PPS: Abnahme um 5,5 lg KbE/cm^2 nach 20 d<br>(a) in Glukose: Abnahme um 3 lg KbE/cm^2 nach 20 d<br>(a) in PPS und 5 % NaCl: Abnahme um 5 lg KbE/cm^2 nach 20 d<br>(a) in Glukose und 5 % NaCl:	rostfreier Edelstahl	15 °C/2 % (a)<br>15 °C/43 % (b)<br>15 °C/75 % (c)	in den ersten d schneller Abfall, danach langsam	[76]

			Abnahme um 2 lg KbE/cm^2 nach 20 d<br>(b) in PPS: Abnahme um 2 lg KbE/cm^2 nach 20 d<br>(b) in Glukose: Abnahme um 2 lg KbE/cm^2 nach 20 d<br>(b) in PPS und 5 % NaCl: Abnahme um 2 lg KbE/cm^2 nach 20 d<br>(b) in Glukose und 5 % NaCl: Abnahme um 0,75 lg KbE/cm^2 nach 20 d<br>(c) in PPS: Abnahme um 4 lg KbE/cm^2 nach 20 d<br>(c) in Glukose: Abnahme um 0 lg KbE/cm^2 nach 20 d<br>(c) in PPS und 5 % NaCl: Abnahme um 3 lg KbE/cm^2 nach 20 d<br>(c) in Glukose und 5 % NaCl: Abnahme um 1 lg KbE/cm^2 nach 20 d				
	zunächst Start mit 3 lg	Streuplattierung auf Agar, Kultivierung	Abnahme um 2 lg nach 21 d;	rostfreier Edelstahl	15 °C/43 %	in Mischbiofilmen unter Austrocknung	[77]

	KbE/cm²; Bebrütung für 48-72 h, um Biofilm zu formieren: 7,3 lg KbE/cm²					wird *L. monocytogenes* schneller inaktiviert als in Monobiofilmen	
Neisseria gonorrhoeae	2 x ~ 20 µl Patienten-exsudat (mit nachgewiesener Infektion)	mit durch warmer Kochsalzlösung befeuchtetem Tupfer auf Agar aufgebracht, Kultivierung	Kultivierbarkeit nach 24 h auf Kunststoff/Laken sehr gering (3 % Wachstum); nach 10 min auf Kunststoff 31 % Wachstum, nach 1 h auf Kunststoff 20 % und auf Laken 16 % Wachstum	Kunststoff und Bettlaken aus Baumwolle/Polyester-Mischgewebe	15-20 °C/k.A.	Aufbringung auf gesamter Oberfläche eines 18 Millimeter (mm) großen Kreises aus Kunststoff und eines 1x1 Zentimeter (cm) großen Quadrats aus sterilem Bettlaken gegeben	[78]
	1 Tropfen positives Urethralsekret	ausgeschüttelt in 0,2 ml Pufferlösung, Eluat auf Agar, Rekultivierung	(a) Glas 2-17 h 3/3*, 24 h ¾, 48 h 0/2; Handtuch 3-6 h 2/2, 24 h 5/9, 48 h 0/2<br>(b) Glas 120 h 4/10	Glas/Handtuch	22 °C (a); 4 °C (b)	*Anzahl positiven Proben/Probenzahl	[79]
Pseudomonas aeruginosa	Probenkörper (1 cm²) bestrichen mit a) Trocken-inokulum: 5-6 lg/cm² oder b) Flüssig-inokulum: ~ 6 lg KbE/ml;	Einlegen Probenkörper in TSB (silberhaltige Proben mit 5 % Pferdeserum neutralisiert), Aufbringen auf Agar, Rekultivierung	a) nach 7 d 6,2 lg/cm² ohne und mit Silber;<br>b) nach 7 d 7,8 lg/cm² ohne und mit Silber	Polymer ohne und mit Silberimprägnierung	37 °C/feuchte Kammer	Teststamm ATCC 15442	[49]

	Applikation von 50 µl						
	8 lg KbE/ml		nach 48 h durchschnittlich < 2 lg KbE/ml	Türgriffe/Stühle auf dem Flur und im Warteraum/Schläuche des Spirometers	37 °C/k.A.		[80]
	7,5 lg KbE/ml (1:1) mit Urin, Blut, Salzlsg. Wasser; Applikation von 20 µl	Resuspension in TSB, Eluat auf Agar, Rekultivierung	alle KT: Abnahme um ~ 1 lg KbE/ml nach 8 Wo	Keramikboden/Baumwollstofffragmente/synthetische Fasern/Eierkarton-Matratzenauflage	RT/k.A.	KT 3 cm^2	[40]
	5,2 lg KbE/Baumwolltuch (2x2 cm)	in 4 ml steriler PBS gemixt, Eluat auf Agar, Rekultivierung	nach 2 h nicht mehr kultivierbar	Baumwolltuch	21 ±1 °C/50 ±5 %		[41]
	~ 6,5 lg KbE/Testfläche	Testfläche auf Agar, Kultivierung	13 d/23 d/33 d/33 d (qualitativ)	Baumwolle/Baumwolle-Polyester/Wolle /Seide	21-24 °C/30-49 %	klinische Isolate, Krankenhaustextilien	[35]
	~ 8,4 lg KbE; Applikation von 0,25 µl/25 cm^2	Kontaktkultur von kontaminierter Fläche, Kultivierung	< 2 d auf allen Oberflächen	Glas/PVC/Stahl/Aluminium, angetrocknet in 0,89 % NaCl-Lsg.	21 °C/31-35 %	Teststamm ATCC27853; Kalkulation der Überlebenszeit bezogen auf 250 KbE in der Kontaktkultur als zählbare Ausgangs-KbE	[36]
	6 lg KbE/ml; Applikation von 0,25 ml auf KT	Kontaktkultur	4 d/5 d/1 d	Formica/Edelstahl/Infusionsständer (Emaille)	20-22 °C/60-70 %	KT 8 cm^2	[46]
	für Trocknungsversuch: 6,4 lg KbE/cm^2, Applikation von 50 µl;	bei Trocknungsversuch: Alufolie in Bouillon ausgeschüttelt, Eluat	0 lg KbE/cm^2 nach 2 d Austrocknung	Austrocknungsversuche auf Alufolie	RT/40-50 %	klinische Isolate	[47]

	für Feuchtigkeits-versuch: 3-4 lg KbE/ml	auf Agar, Rekultivierung<br><br>bei Feuchtigkeits-untersuchungen: Ausspateln der Wasser-Bakterien-suspensionen auf Agar, Rekultivierung	Vermehrung in Leitungswasser (> 12 d), stirbt in Aqua bidest innerhalb von 4 d ab; bei Kaltvernebelung von Leitungs-wasser (bei 30/40 °C) bleibt Vermehrungs-fähigkeit erhalten (> 12 d)	Untersuchungen in feuchtem Milieu in Aqua bidest/sterilem Leitungswasser	RT/k.A.; bei Leitungswasser auch Kaltvernebelung bei 30-40 °C		
	6-7 lg KbE/ml	Direktausstrich auf Agar, Kultivierung	bei 0/32/58/77 % > 8 Mon/bei 99 % > 16 Mon)	Hausstaub	RT/0, 32, 42, 58, 77, 99 %	klinische Isolate, je höher die Feuchtigkeit war, desto häufiger traten Ver-unreinigungen durch Schimmelsporen auf	[48]
Salmonella enteritidis	~ 3-7 lg KbE/cm^2	angefeuchteter Baumwolltupfer, Überimpfung auf Agar, in 10 ml Maximum Recovery Diluent, Suspension auf Agar, Rekultivierung	bei Ausgangs-kontamination 2,4 lg KbE/cm^2 und 3,4 nach 8 h kein Nachweis; bei 5,4 lg Wiederfindung nach 8 h 2 lg, nach 24 h ~ 1,7 lg, nach 48 h kein Nachweis	Kunststoff	25 °C	Verpackungsmaterial	[67]
Salmonella enterica	9 lg KbE/100 µl	Resuspension, Eluat auf Agar, Rekultivierung	bei 5 °C nach 300 d Reduktion um 2-4 lg; bei 25 °C nach	Kunststoff/Lebensmittel	5, 15, 25 °C/ 22, 43, 58, 68, 93 %		[68]

			100 d Reduktion um 6 lg (*Salmonella chester*, *Salmonella oranienburg* > 200 d kultivierbar)				
	~ 9,3 lg KbE/ml	Resuspension in PBS, Eluat auf Agar, Rekultivierung	> 48 h	Petrischale	22-25 °C/40 %	Fokus lag nicht auf der verwendeten Oberfläche, sondern auf der Überlebensfähigkeit von gegen Austrocknen resistente Bakterien	[81]
Salmonella typhimurium	5,2 lg KbE/Baumwoll-tuch (2x2 cm)	in 4 ml steriler PBS gemixt, Eluat auf Agar, Rekultivierung	nach 7 h nicht mehr kultivierbar	Baumwolltuch, Glas	21 ±1 °C /50 ±5 %	k.A. für Glas	[41]
	3,6 lg KbE/ml, Applikation von 20 µl	Resupension, Eluat auf Agar, Rekultivierung	bis zu 6 Wo	Edelstahl	24 °C /45 %	*Salmonella typhimurium* ST4/74, verschiedene genetische Typen wurden auf Einfluss der Trocknung geprüft	[82]
	1 µl der Übernacht-kulturen wurden auf Agar inokuliert und bei 25 °C oder 28 °C für 6 d bebrütet, auf Kunststoff	Rehydratation und Ausplattieren auf Agar	nach 1 Mon bei 25 °C 59,7 ± 12,3 % rekultivierbar	Kunststoff	25 °C oder 28 °C/k.A.	*Salmonella typhimurium* ST19, bei Biofilmbildung ähnliche Rekultivierungsraten	[83]
			nach 1 Mon bei 25 °C waren 13,1 ± 9,6 % rekultivierbar			*Salmonella typhimurium* ST31, bei Biofilmbildung ähnliche Rekultivierungsraten	[83]

	überführt und getrocknet						
	Staub wurde mit wenigen Tropfen der Bakterien-suspension versehen und in Flaschen über unterschiedliche Zeiten fenster-nah aufbewahrt	Resuspension des Staubs mit Nährlösung und Ausplattierung auf Agar, Kultivierung	bis zu 4 Jahre und 2 Mon	Staub vom Fußboden	RT/k.A.		[84]
	5,2 lg KbE/Baumwoll-tuch (2x2 cm)	in 4 ml steriler PBS gemixt, Eluat auf Agar, Rekultivierung	nach 1 d nicht mehr kultivierbar	Baumwolltuch	21 ± 1 °C/50 ± 5 %		[41]
	~ 7-8 lg KbE/ml (= 6-7 lg KbE/Oberfläche); Applikation von 50 µl	Vortexen, Eluat auf Agar, Rekultivierung	mind. 30 d (Abnahme zwischen 3-6 lg KbE/Oberfläche)	Edelstahl	25 °C/33 %	KT Ø 2 cm	[85]
Serratia liquefaciens	7,2 lg KbE, Applikation von 20 µl	Vortexen 15 s in Wasser, Eluat auf Agar, Rekultivierung	mittlere Überlebensrate 3 d bei Antrocknung in Wasser oder Eiweiß	Glas	22 ± 2 °C/31 ± 3	Nachweisgrenze 2 KbE/KT	[26]
	~ 8,4 lg KbE; Applikation von 0,25 µl/25 cm^2	Kontaktkultur von kontaminierter Fläche, Kultivierung	52 KbE nach 2 d, ~ 36 KbE nach 3 d/~ 4 KbE nach 2 d/~ 112 KbE nach 1 d, 50 KbE nach 2 d, ~ 13 KbE nach 3 d/50 KbE nach 2 d	Glas/PVC/Stahl/Aluminium, jeweils angetrocknet in 0,89 % NaCl-Lsg.	21 °C/31-35 %	Teststamm DSM12485, Kalkulation der Über-lebenszeit bezogen auf 250 KbE in der Kon-taktkultur als zählbare Ausgangs-KbE	[36]
Serratia marcescens	~ 8,4 lg KbE; Applikation von 0,25 µl/25 cm^2						

	7,2 lg KbE, Applikation von 20 µl	Vortexen 15 s in Wasser, Eluat auf Agar, Rekultivierung	mittlere Überlebensrate 12 d bei Antrocknung in Wasser, 9 d bei Antrocknung in Eiweiß; nach 4 d ~3 lg Verlust nach 10 d ~ 5 lg Verlust	Glas	22 ± 2 °C/31 ± 3 %	Nachweisgrenze 2 KbE/KT	[26]
	für Trocknungs-versuch: 7,3 lg KbE/cm², Applikation von 50 µl<br><br>für Feuchtigkeits-versuch: 3-4 lg KbE/ml	bei Trocknungs-versuch: Alufolie in Bouillon ausgeschüttelt, Eluat auf Agar, Rekultivierung;<br>bei Feuchtigkeits-untersuchungen: Ausspateln der Wasser-Bakterien-Suspensionen auf Agar, Rekultivierung	2,6 lg KbE/cm² nach 25 d Austrocknung<br><br>Vermehrung in Aqua bidest (> 24 d)/und Leitungswasser (> 12 d)	Austrocknungsversuche auf Alufolie<br><br>Untersuchungen in feuchtem Milieu in Aqua bidest und sterilem Leitungswasser	RT und 40-50 %<br><br>RT/k.A.; bei Leitungswasser auch Kaltvernebelung bei 30-40 °C	klinische Isolate	[47]
	5,2 lg KbE/Baumwoll-tuch (2x2 cm)	in 4 ml steriler PBS gemixt, Eluat auf Agar, Rekultivierung	nach 1 h nicht mehr kultivierbar	Baumwolltuch/Glas	21±1 °C /50±5 %	k.A. für Glas	[41]
Serratia typhimurium	6 lg KbE/ml, Applikation von 25 µl	Resuspension (abgekratzt und abgeschwemmt), Eluat auf Agar, Rekultivierung	Edelstahl: (a, b, c) Abnahme nach 3 d um 4 lg KbE/cm²; (d) Abnahme nach 10 d um 0,25 lg KbE/cm²<br>ABK: (a)Abnahme nach 1 um 4,25 lg KbE/cm²; (b) Abnahme nach 3 d	rostfreier Edelstahl/Acrylnitril-Butadien-Kautschuk (ABK)	25 °C/32,5% (a); 25 °C/75,5 % (b); 6 °C/32,5 % (c); 6 °C/75,5 % (d)		[74]

			um 4,3 lg KbE/cm²; (c) Abnahme nach 10 d um 4,3 lg KbE/cm²; (d) Abnahme nach 2 d um 2,3 lg KbE/cm²				
Shigella dysenteriae	~ 5 lg KbE/cm², Applikation von 10 µl	Vortexen in 1 ml PBS, Eluat auf Agar, Rekultivierung	nach 4 h (nicht mehr kultivierbar), nach 5 d (mittels PCR bzw. Immunofluores-zenztechniken nachweisbar)	Kunststoff/Glas/Aluminium /Holz/Textil	25 °C/k.A., Dunkelheit	Typ 1, Stamm AE 21725; KT 1 cm²	[86]
Shigella sonnei	~ 5,7/5,4/5,1/ 4,8 lg KbE/ml	Resuspension/Eluat auf Agar, Rekultivierung	Überlebensrate im Hellen nach 24 h bei 5,7 und 5,4 lg 100 % /100 %/100 %; bei 5,1 lg 100 %/81 %/81 %; bei 4,8 lg 88 %/69 %/69 %; nach 48 h bei 5,7 lg 75 %/63 %/50 %; bei 4,8 lg 31 %/25 %/19 %; nach 72 h bei 5,7 lg 13 %/0 %/0 %; ab 5,4 lg 0 %/0 %/0 %	PVC /Polystyrol/Sprelacart für 1 h in die Bakteriensuspension	nach Lufttrocknung auf Fließpapier Lagerung im Dunkeln bzw. Hellen bei RT; zwischen heller und dunkler Lagerung kaum Unterschiede/k. A.	Teststamm 730/68; KT 10x10x1-1,5 mm	[87]
Shigella flexneri			Überlebensrate im Hellen nach 24 h bei 5,7 lg 100 %/100 %/83			Teststamm 4a „Bu"	

			%; bei 4,8 lg 50 %/42 %/25 %; nach 48 h bei 4,7 lg 67 %/58 %/33 %; bei 4,8 lg 0 %/0 %/0 %; nach 72 h 0 %/0 %/0 %				
Stenotrophomonas maltophilia	~ 6,5 lg KbE/Testfläche	Testfläche auf Agar, Kultivierung	7 d/7 d/7 d/7 d (qualitativ)	Baumwolle/Baumwolle-Polyester/Wolle /Seide	21-24 °C/30-49 %	klinische Isolate, Krankenhaustextilien, Rekultivierung bis 52 d nach Kontamination	[35]
Vibrio cholerae	8,2 lg KbE; Applikation von 50 µl	Vortexen in 2 ml 0,89 % NaCl, Eluat auf Agar, Rekultivierung	normal kultivierbarer Status 1 h/1 h/1,5 h/1,5 h /1,5 h/3,5 h/4 h/4 h; VBNC Status bis 7 d	Aluminium/Glas/Kunststoff /Stahl/Eisen/Papier/Textil/ Wolle	RT bei Dunkelheit/k.A.	Teststamm O1 El Tor N16961; 50 µl/KT 1 cm^2	[88]

2.1.2 Pilze

Schimmelpilze kommen in der Natur ubiquitär vor, sind thermotolerant und können auf Oberflächen abhängig vom Material 2 d bis > 30 d überleben (**Tab. 3**). Es ist zu berücksichtigen, dass sich Schimmelpilze ab einer RLF ≥ 75 % bei RT vermehren können, wodurch es zu Schimmelpilzbefall kommen kann [89]. Schimmelpilzsporen gelangen in der Regel über die Atemluft in die tiefen Atemwege; hier können sie bei hochgradig immunsupprimierten Patienten invasive Mykosen auslösen [90]. Auf Oberflächen im Krankenhaus wurden am häufigsten die Schimmelpilzspezies *Cladosporium*, *Aspergillus* und *Penicillium* nachgewiesen [91-93]. *Mucor* und *Aspergillus (A.)* spp. wurden im Staub einer Klimaanlage mit defektem Filter und in der Raumluft isoliert und waren offenbar mit mykotischer Endokarditis bei Patienten nach offener Herzchirurgie assoziiert [94].

Die Dermatophyten *Epidermophyton (E.) floccosum, Trichophyton (T.) mentagrophytes* und *Tricholosporum violaceum* überlebten in Hautschuppen 10 Jahre bei -20 °C, während *T. rubrum* und *T. verrucosum* unter gleichen Bedingungen nicht mehr kultivierbar waren [95]. Auf Oberflächen im Krankenhaus wurde z.B. *Microsporum canis* nachgewiesen [93]. Während in Deutschland in den 20er Jahren des vorigen Jahrhunderts *E. floccosum* und *Microsporum (M.) audouinii* als Erreger humanpathogener Dermatophytosen dominierten und *T. rubrum* nahezu unbedeutend war, stieg dessen Anteil an Dermatophytenisolaten von 41,7 % im Jahr 1950 auf 82,7 % im Jahr 1993, so dass *T. mentagrophytes var. interdigitale* nach und nach durch *T. rubrum* als Haupterreger der Tinea pedis und der Onychomykose abgelöst wurde. Mit der Einführung von Griseofulvin im Jahr 1958 wurden sowohl *M. audouinii* als auch *T. schoenleinii* praktisch ausgerottet [96]. Bei *Tinea pedis* war *T. rubrum* bei 86 % der Patienten, *T. mentagrophytes* bei 81 % der Patienten im Hausstaub nachweisbar [97]. Auch nach dem Verlassen von öffentlichen Bädern wurden beide Dermatophytenspezies an den bloßen Fußsohlen nachgewiesen. Durch Waschen und Abtrocknen fand keine komplette Elimination statt [98]. Seit Anfang des 20. Jahrhunderts steigt die Inzidenz von *Microsporum canis*-Infektionen in Europa, speziell in den mediterranen Ländern und Slowenien, stark an, wobei Hunde und Katzen das natürliche Reservoir sind [99]. Eine Weiterverbreitung ist jedoch auch über Kämme, Bürsten, Hüte, Möbel, Bettwäsche usw. möglich.

Sprosspilze können abhängig von den Expositionsbedingungen bis zu 3 Monaten überleben, obwohl die Überlebensdauer im Allgemeinen nur im Bereich von d, also kürzer als bei Schimmelpilzen und Dermatophyten ist (**Tab. 3**). Auch gegen Sprosspilze wie *Candida* spp. hat der menschliche Organismus durch die ständige Exposition ein funktionierendes Abwehrsystem entwickelt, so dass invasive Pilzinfektionen meist nur bei eingeschränkter Immunabwehr, Beeinträchtigung des Mikrobioms oder Katheter-assoziiert als endogene Infektion auftreten [100]. Infektionen können aber auch durch den Erwerb virulenter Stämme aus dem Patientenumfeld verursacht werden [101, 102].

Wie bei Bakterien erhöht sich auch bei Sprosspilzen die Persistenz bei Einbettung in eiweißhaltigen Medien [20, 27].

Bei Sprosspilzarten nahm die Überlebensdauer ubiquitär verbreiteter Arten wie *Rhodotorula rubra* und apathogener Cryptococcus-Arten sowie von *Cryptococcus neoformans* mit sinkender RLF zu. Sie waren bei 1 % RLF besonders lange lebensfähig. Dagegen überlebten bei hoher Luftfeuchte Schleimhautbesiedler wie *Candida (C.) glabrata, C. albicans* und *C. tropicalis* länger als bei Trockenheit. Eine Mittelstellung nahmen Arten ein, die häufig auf der Körperoberfläche des Menschen anzutreffen sind *wie C. parapsilosis* [20].

Bei 15 Sprosspilzarten verlängerte sich die Überlebenszeit mit Erniedrigung der Umgebungstemperatur. Insgesamt war die Überlebensfähigkeit bei den untersuchten Spezies bei 4 °C und 1 % RLF am längsten und bei 37 °C und 96 % RLF am kürzesten [20]. Anders verhält es sich bei der Freisetzung von Bioaerosolen im Innenraum. Bei 25 °C wurden mehr Pilze (Hauptanteil *Fusarium* und *Penicillium* spp.) freigesetzt als bei 37 und 15 °C, wobei sich die Zusammensetzung der Schimmelpilzarten in den 3 Temperaturbereichen deutlich unterschied [103].

Auch Lichtverhältnisse beeinflussen die Persistenz. Die Überlebensdauer von auf glatten Glasflächen angetrockneten *C. albicans* und *Rhodotorula rubra* verdoppelte sich bei Aufbewahrung in Dunkelheit im Vergleich zum Tageslicht und verlängerte sich z.B. für *C. albicans* von 44 auf 98 d [20].

In der Patientenumgebung gelang der Nachweis von *Candida* spp. (*C. glabrata*, *C. parapsilosis*, *C. tropicalis*, *C. albicans*, *C. metapsilosis*, *C. lusitaniae*) von trockenen Oberflächen in ~ 3 %, von feuchten Oberflächen in ~ 14 %, von MRSA und VRE von trockenen Flächen in ~ 4 %, von Fluorchinolon resistenten Gram-negativen Erregern von trockenen Oberflächen in ~ 1 % und von feuchten Oberflächen in ~ 8 % [104]. Im Unterschied zu anderen *Candida* spp. wurden durch *C. auris* Ausbrüche verursacht [105], d.h. *C. auris* unterscheidet sich in der nosokomialen Ausbreitung von anderen Sprosspilzen und Dermatophyten [106], indem auch durch die Umgebungskontamination eine Übertragung vor allem über die Hände und Medizinprodukte mit Ausbruchspotential möglich ist [106, 107]. Inzwischen sind sogar panresistente Stämme aufgetreten [108].

Tabelle 3: Persistenz von Schimmel- und Sprosspilzen und Bedingungen zur Rekultivierung

Erreger	Ausgangsinokulum	Rückgewinnung zur Kultivierung	Tenazität	Oberfläche	Temperatur/RLF	Bemerkung	Quelle
A. flavus	4-5 lg KbE/Testkörper	Testkörper (TK) in Bouillon, Eluat auf Agar, Rekultivierung	2 bis > 30 d/2-20 d/> 30 d/8 bis> 30 d (qualitativ)	Baumwolle/Polyester/ Polyethylen/Poly-urethan	22-25 °C/25-47 %	Isolierung unterschiedlicher Stämme aus Verbrennungswunden	[109]
	~ 5,5 lg KbE/Testkörper	TK in Bouillon (vortexen), Eluat auf Agar, Rekultivierung	Wiederfindung ~ 5,4 lg nach 24 h; ~ 5,2 lg nach 48 h; ~ 5,6 lg nach 5 d/~ 5,3 lg nach 24 h; ~ 3,8 lg nach 48 h; 0 lg nach 5 d	Aluminium/Kupfer	Raumklima (RK)		[110]
A. fumigatus	4-5 lg KbE/Testkörper	TK in Bouillon, Eluat auf Agar, Rekultivierung	1 bis > 30 d/5 bis > 30 d/> 30 d/5 bis > 30 d (qualitativ)	Baumwolle/Polyester/ Polyethylen/Poly-urethan	22-25 °C/25-47 %	Isolierung unterschiedlicher Stämme aus Verbrennungswunden	[109]
	~ 6,8 lg KbE/Testkörper	TK in Bouillon (vortexen), Eluat auf Agar, Rekultivierung	Wiederfindung ~ 6,3 lg nach 24 h; ~ 6,4 lg nach 96 h, 120 h und 24 h/~ 6 lg nach 48 h; ~ 1,7 lg nach 5 d	Aluminium/Kupfer	RK		[110]
	~ 6,5 lg KbE/Testfläche	TK auf Agar, Eluat auf Agar, Rekultivierung	> 30 d/> 30 d/> 30 d/27 d (qualitativ)	Baumwolle/Polyester/ Wolle/Seide	21-24 °C/30-49 %	klinische Isolate, Krankenhaustextilien,	[35]
A. niger	4-5 lg KbE/Testkörper	TK in Bouillon, Eluat auf Agar, Rekultivierung	3 bis > 30 d/> 30 d/> 30 d/2 bis > 30 d (qualitativ)	Baumwolle/Polyester/ Polyethylen/Poly-urethan	22-25 °C/25-47 %	Isolierung unterschiedlicher Stämme aus Verbrennungswunden	[109]
	~ 5,3 lg KbE/Testkörper	TK in Bouillon (vortexen), Eluat auf Agar, Rekultivierung	Wiederfindung ~ 5,2 lg nach 4 d; ~ 5,5 lg nach 24 d/~ 5 lg nach	Aluminium/Kupfer	RK		[110]

			4 d; ~ 5,1 lg nach 5 d; ~ 5,4 lg nach 24 d				
A. terreus	4-5 lg KbE/Testkörper	TK in Bouillon, Eluat auf Agar, Rekultivierung	2 bis > 30 d/2 bis > 30 d/> 30 d/12 bis > 30 d (qualitativ)	Baumwolle/Polyester /Polyethylen/Poly-urethan	22-25 °C/25-47 %	Isolierung unterschiedlicher Stämme aus Verbrennungswunden	[109]
C. albicans	4-5 lg KbE/Testkörper	TK in Bouillon, Eluat auf Agar, Rekultivierung	1-3 d/1 d/5-6 d/4-5 d (qualitativ)	Baumwolle/Polyester/ Polyethylen/Poly-urethan	22-25 °C/25-47 %	Isolierung unterschiedlicher Stämme aus Verbrennungswunden	[109]
	6 lg KbE/Testkörper	TK in Bouillon (vortexen), Eluat auf Agar, Rekultivierung	Rekultivierung bis 7 d nach Kontamination	Stahl (trocken)/feuchter Agar ohne Nährstoffe		Laborstamm; Wiederfindung (%) ~ 18/~ 89	[104]
	6 lg KbE / Testkörper	TK in Bouillon (vortexen), Eluat auf Agar, Rekultivierung	Wiederfindung (%) ~ 1/~ 1 nach 2 d; ~ 0,2/0,3 nach 3 d, 0/0 nach 7 d	Stahl/Glas (Belastung mit Muzin, BSA und Trypton)	22 ± 2 °C/45-62 %	klinisches Isolat	[111]
	~ 7,5 lg KbE/Testkörper	TK in Bouillon (vortexen), Eluat auf Agar, Rekultivierung	Wiederfindung ~ 7,4 lg nach 3 h; ~ 7,5 lg nach 6 h; ~ 6,8 lg nach 24 h; ~ 6,6 lg nach 48 h; ~ 6,5 lg nach 5 d /~ 4,9 lg nach 3 h, 5 lg nach 6 h; 0 lg nach 24 h	Aluminium/Kupfer	RK		[110]
	6,5 lg KbE/Testfläche	Testfläche auf Agar, Kultivierung	6 d/6 d/12 d/12 d (qualitativ)	Baumwolle/Polyester/ Wolle/Seide	21-24 °C/30-49 %	klinische Isolate, Krankenhaustextilien	[35]
	~ 6,1 lg KbE/KT	Überschichten mit Agar, Kultivierung	6 d (qualitativ)	Glaspetrischale	RT/50 %		[20]
	~ 4,8 lg KbE/Testfläche		120 d, 77 d, 48 d bzw. 34 d (qualitativ)	Leinen, Antrocknen in 80 % Serum, 2,5 % Albumin, Urin bzw. PBS	RT/49 %		

			4 d, 11 d bzw. 35 d (qualitativ)	Leinen, Antrocknen in PBS, Lagerung im Dunkeln	37 °C/68 %, 25 °C/38 % bzw. 4 °C/31 %		
	~ 6,8 lg/ml	wöchentlich Ausplattieren auf Agar, Kultivierung	nach 2 Wo Abfall auf ~ 2-4,5 lg, nach 52 Wo ~ 1,3-2,2 lg	Salzlösung 0,6/1,5/2,5/3,4/5 %; Tag-/Nachtrhythmus und Dunkelkontrolle 3,4 % NaCl	20, 25, 30, 35 °C	Isolierung von Strandsand in Hawaii; 10 d Kultivierung, Abschwemmen mit 0,35 % NaCl-Lsg., Überführung in Salzlösung mit steigender Temperatur rascheres Absterben; keine Korrelation zum Salzgehalt	[112]
	Probenkörper (1 cm^2) bestrichen mit Trockeninokulum: 5-6 lg/cm^2	Einlegen Probenkörper in TSB (silberhaltige Proben mit 5 % Pferdeserum neutralisiert), Eluat auf Agar, Rekultivierung	nach 7 d 6,3 KbE/cm^2 ohne Silber; nach 7 d 5,1 KbE/cm^2 mit Silber	Polymer ohne und mit Silberimprägnierung	37 °C/feuchte Kammer	Teststamm ATCC 10231	[49]
C. auris	6 lg KbE/Testkörper	TK in Bouillon (vortexen), Eluat auf Agar, Rekultivierung	Wiederfindung (%) nach 7 d ~ 38/~ 93	Stahl (trocken)/feuchter Agar ohne Nährstoffe		Laborstamm; Rekultivierung bis 7 d nach Kontamination	[104]
	~ 4,8 lg KbE/Testkörper	Anreicherungsbouillon und Direktausstrich, Rekultivierung	Wiederfindung (lg) nach 4 d ~ 3,5; nach 1 d, ~ 3,0; nach 14 d ~ 0,4	strukturierte Kunststoffoberfläche	25 °C/57 %	multiresistenter Stamm; Esteraseaktivität bis 28 d	[113]
	8 lg KbE/Kunststoff-deckglas	Auswaschen und US, Eluat auf Agar, Rekultivierung	in planktonischer Suspension nach 14 d 2 lg KbE/ml rekultivierbar; nach Adhäsion für 90 min an Kunststoff	Kunststoff, die Antrocknung erfolgte in PBS, 10 % fetalem Kälberserum oder künstlichem Speichel	RT/k.A.	durch Verwendung unterschiedlicher klinischer Isolate für die Zellsuspension und den Biofilm ist die	[114]

			(Biofilmbildung) nach 14 d ~ 4,3 lg KbE/ml rekultivierbar			Vergleichbarkeit evtl. beeinflusst	
C. candidum	~ 6,5 lg KbE/Testfläche	Testfläche auf Agar, Kultivierung	21 d/6 d/12 d/6 d (qualitativ)	Baumwolle/Polyester/ Wolle /Seide	21-24 °C/30-49 %	klinische Isolate, Krankenhaustextilien	[35]
C. glabrata	6 lg KbE/Testkörper	TK in Bouillon (vortexen), Eluat auf Agar, Rekultivierung	Wiederfindung (%) nach 7 d ~ 60/~ 90 nach 7 d	Stahl (trocken)/feuchter Agar ohne Nährstoffe	RT/k.A.	Laborstamm	[104]
	~ 4,8 lg KbE/ Testfläche	Überschichten mit Agar, Kultivierung	150 d, 102 d, 97 d bzw. 86 d (qualitativ)	Leinen, Antrocknen in 80 % Serum, 2,5 % Albumin, Urin bzw. PBS	RT/49 %		[20]
			12 d (qualitativ)	Glaspetrischale	RT/49 %		
	~ 4,8 lg KbE/Testfläche		15 d, 18 d bzw. 130 d (qualitativ)	Leinen, Antrocknen in PBS, Lagerung im Dunkeln	37 °C/68 %, 25 °C/38 % bzw. 4 °C/31 %		
	~ 6,5 lg KbE/Testfläche	Testfläche auf Agar	> 30 d/> 30 d/> 30 d/> 30 d (qualitativ)	Baumwolle/Polyester/ Wolle /Seide	21-24 °C/30-49%	klinische Isolate, Krankenhaustextilien	[35]
C. krusei	4-5 lg KbE/Testkörper	TK in Bouillon, Eluat auf Agar, Rekultivierung	1 d/8 d/3-7 d/4 d (qualitativ)	Baumwolle/Polyester/ Polyethylen/Poly-urethan	22-25 °C/25-47 %	Isolierung unterschiedlicher Stämme aus Verbrennungswunden	[109]
	~ 6,5 lg KbE/ Testfläche	Testfläche auf Agar, Kultivierung	3 d/6 d/> 30 d/21 d (qualitativ)	Baumwolle/Polyester/ Wolle/Seide	21-24 °C/30-49 %	klinische Isolate, Krankenhaustextilien	[35]
C. parapsilosis	4-5 lg KbE/ Testkörper	TK in Bouillon, Eluat auf Agar, Rekultivierung	9-27 d/27 bis > 30 d/> 30 d/> 30 d (qualitativ)	Baumwolle/Polyester/ Polyethylen/Poly-urethan	22-25 °C/25-47 %	Isolierung unterschiedlicher Stämme aus Verbrennungswunden	[109]
	6 lg KbE/Testkörper	TK (vortexen), Eluat auf Agar, Rekultivierung	Wiederfindung (%) nach 2 d ~ 21/~ 21,6; nach 3 d ~ 13,7/~ 14,9; nach 7 d ~ 1,9/~	Stahl/Glas (Belastung mit Muzin, BSA und Trypton)	22 ± 2 °C/45-62 %	klinisches Isolat	[111]

			3,9; nach 14 d ~ 1,3/~ 4,1				
	6 lg KbE/Testkörper	TK in Bouillon (vortexen), Eluat auf Agar, Rekultivierung	Wiederfindung (%) nach 7 d 60/100	Stahl (trocken)/feuchter Agar ohne Nährstoffe		Laborstamm	[104]
	~ 4,7 lg KbE/ Testkörper	Anreicherungs-bouillon, Eluat auf Agar bzw. Direktausstrich, Rekultivierung	Wiederfindung (lg) nach 1 d ~ 4,4 lg; nach 4 d ~ 4,3 lg nach 14 d ~ 3,3 lg; nach 21 d ~ 2,5 lg; nach 28 d 0,4 lg	strukturierte Kunststoffoberfläche	25 °C/57 %	multiresistenter Stamm; Esteraseaktivität bis 28 d	[113]
	~ 6,5 lg KbE/ Testfläche	Testfläche auf Agar, Kultivierung	> 30 d/> 30 d/> 30 d/> 30 d (qualitativ)	Baumwolle/Polyester/ Wolle /Seide	21-24 °C/30-49 %	klinische Isolate, Krankenhaustextilien	[35]
	~ 6,1 lg KbE/KT	Überschichten mit Agar, Kultivierung	55 d	Glaspetrischale	RT/50 %		[20]
C. tropicalis	4-5 lg KbE/Testkörper	TK in Bouillon, Eluat auf Agar, Rekultivierung	1-2 d/1-8 d/7-18 d/6-12 d (qualitativ)	Baumwolle/Polyester/ Polyethylen/Poly-urethan	22-25 °C/25-47 %	Isolierung unterschiedlicher Stämme aus Verbrennungswunden	[109]
	~ 6,6 lg KbE/Testfläche	Testfläche auf Agar, Kultivierung	3 d/9 d/> 30 d/21 d (qualitativ)	Baumwolle/Polyester/ Wolle /Seide	21-24 °C/30-49 %	klinische Isolate, Krankenhaustextilien	[35]
	~ 6,1 lg KbE/KT	Überschichten mit Agar, Kultivierung	8 d	Glaspetrischale	RT/50 %		[20]
Cryptococcus neoformans	~ 6,5 lg KbE/Testfläche	Testfläche auf Agar, Kultivierung	> 30 d/> 30 d/> 30 d/> 30 d (qualitativ)	Baumwolle, Baumwolle/Polyester/ Wolle /Seide	21-24 °C/30-49 %	klinische Isolate, Krankenhaustextilien	[35]
	~ 6,1 lg KbE/KT		27 d (qualitativ)	Glaspetrischale	RT/50 %		[20]

	~ 4,8 lg KbE/Testfläche	Überschichten mit Agar, Kultivierung	70 d, 102 d bzw. > 490 d (qualitativ)	Leinen, Antrocknen in PBS, Lagerung im Dunkeln	37 °C/68 %, 25 °C/38 % bzw. 4 °C/31 %		
Fusarium solani	~ 5,8 lg KbE/Testkörper	TK in Bouillon (Vortexen), Eluat auf Agar, Rekultivierung	Wiederfindung nach 3 h ~ 5,9 lg; nach 6 h ~ 5,8 lg; nach 24 h ~ 5,1 lg; nach 5 d ~ 4,4 lg;/nach 3 h ~ 4,5 lg; nach 6 h ~ 3,6 lg; nach 24 h 0 lg	Aluminium/Kupfer	RK	ähnliche Ergebnisse für *Fusarium (F.) culmonium*, *F. oxysporium*, *Penicillium chrysogenum*	[110]
Microsporum gypseum	~ 6,5 lg/ml	wöchentlich Ausplattieren auf Agar, Kultivierung	nach 2 Wo Abfall auf ~ 2,7-4,6 lg, nach 52 Wo Abfall auf ~ 2,0-3,4 lg	Salzlösung 0,6/1,5/2,5/3,4/5 %; Tag-/Nachtrhythmus und Dunkelkontrolle 3,4 % NaCl	20, 25, 30, 35 °C	Isolierung von Strandsand in Hawaii; 10 d Kultivierung, Abschwemmen mit 0,35 % NaCl-Lsg., Überführung in Salzlösung, mit steigender Temperatur rascheres Absterben; keine Korrelation zum Salzgehalt	[112]
Mucor spp.	4-5 lg KbE/ Testkörper	TK in Bouillon, Eluat auf Agar, Rekultivierung	21 d/24 d/21 d/20 d (qualitativ)	Baumwolle/Polyester/ Polyethylen/Poly-urethan	22-25 °C/25-47 %	Isolierung unterschiedlicher Stämme aus Verbrennungswunden	[109]
Paecilomyces spp.	4-5 lg KbE/ Testkörper	TK in Bouillon, Eluat auf Agar, Rekultivierung	< 1 d/5 d/4 d/11 d (qualitativ)	Baumwolle/Polyester/ Polyethylen/Poly-urethan	22-25 °C/25-47 %	Isolierung unterschiedlicher Stämme aus Verbrennungswunden	[109]
Rhodotorula rubra	~ 6,1 lg KbE/KT	Überschichten mit Agar, Kultivierung	40 d	Glaspetrischale	RT/50 %		[20]
	~ 4,8 lg KbE/ Testfläche		350 d, 308 d, 205 d bzw. 253 d	Leinen, Antrocknen in 80 % Serum, 2,5 %	RT/49 %		

			(qualitativ)	Albumin, Urin bzw. PBS			
			32 d, 85 d bzw. 385 d (qualitativ)	Leinen, Antrocknen in PBS	37 °C/68 %, 25 °/38 % bzw. 4 °C/31 %		
Saccharomyces cerevisiae	~ 4-6 lg KbE/cm²	angefeuchteter Baumwolltupfer, in 10 ml Maximum Recovery Diluent, Suspension auf Agar, Rekultivierung	bei Ausgangs-kontamination von 4,7 lg nach 8 h Wiederfindung ~ 1,3 lg, nach 24 h bei 1 lg; bei Ausgangs-kontamination von 6,4 lg nach 48 h Wiederfindung 3,9 lg	Kunststoff	25 °C	Verpackungsmaterial	[67]
Trichosporon cutaneum	~ 7,5 lg/ml	wöchentlich Ausplattieren auf Agar	nach 2 Wo Abfall auf ~ 2,8-6,5 lg, nach 6-7 Wo kein Überleben	Salzlösung 0,6/1,5/2,5/3,4/5 %; Tag-/Nachtrhythmus und Dunkelkontrolle 3,4 % NaCl	20, 25, 30, 35 °C	Isolierung von Strandsand in Hawaii; 10 d Kultivierung, Abschwemmen mit 0,35 % NaCl-Lsg., Überführung in Salzlösung, mit steigender Temperatur rascheres Absterben; keine Korrelation zum Salzgehalt	[112]
Trichophyton menta-grophytes	~ 6,5 lg; 10 d Kultivierung, Abschwemmen mit 0,35 % NaCl-Lösung, Überführung in Salzlösung		nach 2 Wo Abfall auf ~ 3,2-5,6 lg, Abfall nach 52 Wo auf ~ 1,6-3,2 lg				

2.1.3 Viren

Inwieweit Flächen als Infektionsquelle für Viren relevant sind, lässt sich bisher nur indirekt anhand der Verfolgung der Virusausbreitung vom Patienten und des Vorkommens von Viren in der Patientenumgebung ableiten, da die infektiöse Dosis mit wenigen Ausnahmen nicht bekannt ist. In beiden Situationen steigt die Infektionsgefährdung mit höherer Tenazität. Viren aus dem Darmtrakt können ihre Infektiosität bei RT recht lange behalten, wobei das Spektrum zwischen mehreren Stunden und 3 Monaten variiert. Blutübertragbare Viren sind etwa 1 Woche stabil (**Tab. 4**). Die meisten respiratorischen Viren behalten ihre Infektiosität auf unbelebten Oberflächen nur wenige d (**Tab. 4**, [115]). Für SARS-CoV-2 ist die Übertragung über Flächen offenbar nicht relevant [116], auch wenn die Tenazität auf Flächen bis zu 7 d nachweisbar war (**Tab. 4**).

Bei der Untersuchung von Einflussfaktoren auf die Tenazität ist zu berücksichtigen, dass das Ergebnis jeweils nur für die untersuchten Virusspezies zutrifft. Behüllte Viren, insbesondere respiratorische Viren wie Influenza-, Parainfluenza-, Corona-, Respiratory Syncytial-, Masern- und Rötelnviren, aber auch Herpes simplex- und Varizella-zoster-Viren behalten die Replikationsfähigkeit länger bei geringer RLF von 20-30 % [23]. Nur das Zytomegalievirus wird häufiger von feuchten Oberflächen isoliert [117]. Unbehüllte Viren wie Adeno-, Entero- und Rhinoviren sind länger replizierfähig bei 70-90 % RLF (**Tab. 4**, [118]).

Für die meisten Viren, wie Astro-, Adeno-, Polioviren, Herpes simplex- und Hepatitis-A-Virus ist eine niedrige Temperatur (4 °C) mit längerer Dauer der Replizierbarkeit verbunden [119]. Bei enterischen Viren stieg die Tenazität in Wasser mit steigender Temperatur > 20 °C [120, 121]. Bei Noro-, Polio- und Hepatitis A Viren war die Tenazität bei > 80 % RLF höher [122]. Das wurde für das Poliovirus bestätigt, indem die Stabilität bei 95 % RLF signifikant größer war als bei 25 % RLF [123]. Bei Coronaviren war der Einfluss der RLF unterschiedlich mit höherer Tenazität bei 20 % und 80 % und vergleichsweise niedrigerer Tenazität bei 50 % [124].

Auch organische Belastung (Verschmutzung) beeinflussen die Tenazität von Viren auf Flächen (**Tab. 4**).

Bei SARS-CoV-2 war der Einfluss von interferierenden Substanzen, Temperatur (20 oder 35 °C) und RLF meist nur von moderatem Einfluss (**Tab. 4**). Morris et al. [125] haben auf der Grundlage der Prüfung der Stabilität von SARS-CoV-2 auf einer inerten Oberfläche bei neun Temperatur- und Feuchtigkeitsbedingungen mit einem mechanistischen, quantitativen Modell erstmals eine Vorhersage abgeleitet, wie Temperatur und Feuchtigkeit die Tenazität verändern. SARS-CoV-2 blieb am längsten bei niedrigen Temperaturen und extremer relativer Luftfeuchtigkeit (bis 85 %) infektiös. Die geschätzte mittlere Halbwertszeit des Virus betrug > 24 h bei 10 ˚C und 40 % RLF, aber ~ 1,5 h bei 27 ˚C und 65 % RLF. Das Modell erklärt anhand der chemischen Grundlagen, warum die Inaktivierungsrate bei

behüllten Viren mit steigender Temperatur zunimmt und eine U-förmige Abhängigkeit von der Luftfeuchtigkeit aufweist. Das Modell prognostiziert genau die vorhandenen Ergebnisse zum Einfluss von Temperatur und RLF bei fünf verschiedenen humanen Coronaviren. Das deutet darauf hin, dass gemeinsame Mechanismen die Stabilität vieler Viren beeinflussen können.

Auf porösen Oberflächen überleben z.B. Corona-, Influenza-, das Avian-Metapneumovirus, Poliovirus Typ 1 und humanes enterisches Adenovirus Typ 40 [122, 126] länger als auf nicht porösen Oberflächen (**Tab. 4**). Ein Grund kann die geringere Viruselution bei der Rückgewinnung aus porösen Materialien sein [127]. Auch die Kapillarwirkung innerhalb der Hohlräume und die schnellere Verdampfung der Aerosole könnte von Einfluss sein [128]. Auf Kupferoberflächen ist die Tenazität von Viren aufgrund der oligodynamischen Wirkung von Kupfer geringer als auf Kunststoffoberflächen (**Tab. 4**).

Erwartungsgemäß ist auch die Menge des Inokulums von Einfluss, nachgewiesen, z.B. für das transmissible Gastroenteritis- und das Maushepatitisvirus [124]. Auch bei SARS-CoV-2 waren geringere Inokula mit kürzerer Tenazität von 2-4 d bis zum Verlust der Infektiosität [129, 130] im Vergleich zu längeren Zeiten von 21 d [131] bzw. 7-28 d [132] bei größeren Inokula assoziiert.

Bei SARS-CoV-2 wurde nachgewiesen, dass unter dem Einfluss von simuliertem Sonnenlicht 90 % der Viren, die in künstlichem Speichel auf die Oberfläche aufgebracht wurden, bei simulierter sommerlicher Exposition alle 6,8 min, bei winterlicher Exposition alle 14,3 min inaktiviert wurden [133]. Dagegen war im Dunkeln innerhalb von 1 h kein signifikanter Abfall feststellbar (**Tab. 4**; [133]). Auch im Aerosol war der Einfluss von Sonnenlicht reproduzierbar, während die RLF allein (20-70 %) ohne Einfluss war [134]. Durch Bestrahlung (Abstand 3 cm) mit UVC (Dosis 1,048 Millijoule (mJ)/cm^2) wurde SARS-CoV-2 (infektiöser Titer von 5×10^6 $TCID_{50}$/ml) nach 9 min komplett inaktiviert, während durch UVA (Dosis 292 mJ/cm^2) der Titer nach 9 min nur um 1 lg reduziert wurde [135].

Bisher wurde nicht untersucht, ob nach Anwendung von Reinigungs- und Desinfektionsmitteln zurückbleibende Rückstände Einfluss auf die Tenazität haben.

Unter Berücksichtigung aller Einflussfaktoren können unter Laborbedingungen generierte Daten nur eine grobe Orientierung geben. Im Zweifelsfall ist bei der Bewertung der Daten in **Tab. 4** von der ungünstigen Situation auszugehen.

Die Relevanz von Oberflächen in Gesundheitseinrichtungen als Kontaminationsquelle für Viren ist noch schwieriger als für Mikroorganismen nachzuweisen, weil die Isolierung von Flächen deutlich aufwendiger ist. Nach Entlassung von Patienten mit einer Norovirusinfektion kam es nach Neuaufnahme zu Infektionen, weil die Infektionsdosis für Noroviren sehr gering ist (1 bis 10 bis 100 Viruspartikel) [136]. Ein großer Ausbruch durch Noroviren konnte nur durch Schließung der

betroffenen Abteilungen, umfangreiche Desinfektion und Beurlaubung bzw. Kohortenbildung des Personals beherrscht werden [137]. In einer retrospektiven Kohortenstudie ergab sich allerdings ein sehr geringes Risiko, weil nur 2 von 1106 exponierten Patienten den identischen Norovirusstamm vom entlassenen Patienten erworben hatten [138]. Obwohl bei der nosokomialen Übertragung von Rotaviren die Hand dominiert, sind auch Flächen relevant für die Verbreitung [139]. Bei einem Simulationsversuch zur Virusausbreitung über Flächen unter Verwendung des Cauliflower Mosaikvirus konnte gezeigt werden, dass das Virus innerhalb von 10 h außerhalb der Isoliereinheit auf 41 % der beprobten Oberflächen nachweisbar war [140]. Ob die Menge zur Infektionsübertragung ausreicht, wurde nicht untersucht. Nach dem Auftreten von MERS wurde, obwohl der Ursprung zoonotisch ist, das Risiko der Weiterverbreitung über Flächen untersucht. Im Ergebnis wurde in der Umgebung hospitalisierter beatmeter Patienten trotz eines strengen Desinfektionsregimes und Unterdruckbelüftung eine Kontamination mit Virus-RNA nachgewiesen. Aufgrund der Tenazität von bis zu 9 d (**Tab. 4**) und des Vorkommens in der Patientenumgebung schlussfolgerten die Autoren, dass eine sorgfältige insbesondere patientennahe Flächendesinfektion zur Prävention beitragen kann [141], obwohl der Nachweis von RNA nicht mit Infektiosität gleichzusetzen ist. Bei SARS-CoV-2 wurde untersucht, ob das Tröpfchenvolumen (1-50 µl) die Virusansammlung auf einer Fläche [142] beeinflusst. Es ergab sich keine Abhängigkeit.

SARS-CoV-2 ist ein eindrückliches Beispiel, wie die Prophylaxe einer neuen Infektionskrankheit anhand der Erkenntnisse zur Tenazität, Chemoresistenz und Übertragung des infektiösen Agens auf der Basis krankenhaushygienischer Strategien in atemberaubendem Tempo etabliert wurde und fortlaufend angepasst wird. Wie die anderen Coronaviren wurde SARS-CoV-2 auf Flächen [143] mit einer Korrelation zwischen Patientennähe und Flächenkontamination nachgewiesen [144], so dass das Risiko einer Weiterverbreitung aufgrund der Tenazität von bis zu 7 d auf Flächen (**Tab. 4**) durch sorgfältige Flächendesinfektion zu unterbinden ist [145, 146] (**Tab. 4**). Das gilt für alle Materialien (**Tab. 4**), auch wenn ihr Einfluss auf die Tenazität unterschiedlich ist [147]. Zeitabhängig sinkt die rückgewinnbare Virusmenge nahezu linear und ist auf Kunststoff nach 72 h, Edelstahl nach 48 h, Karton nach 24 h und Kupfer nach 4 h unkritisch [148]. Da die infektiöse Dosis unbekannt ist, bleibt die Risikoabschätzung offen. In einem Fallbericht wird das Vorkommen von SARS-CoV-2 auf Oberflächen im Haushalt so interpretiert, dass eine Übertragung von Flächen infrage kommt, wenn diese kürzlich durch Husten oder Niesen kontaminiert, berührt und nachfolgend Mund, *Vestibulum nasi* oder Augen berührt wurden [149]. Wie in dieser Bewertung ist auch in den wenigen anderen Fällen, in denen eine Übertragung über Flächen vermutet wird, eine Übertragung über die Atemwege nicht auszuschließen [150]. Vermutlich ist das Infektionsrisiko jedoch nicht sehr hoch, weil in Abstrichproben von Oberflächen auf einer Notfallstation und einer Station für subintensive Pflege nur in zwei von 26 Proben geringe Mengen von SARS-CoV-2-RNA nachweisbar waren, die in der Zellkultur keinen

zytopathischen Effekt auslösten [151]. Möglicherweise reduzieren Rückstände der eingesetzten Flächendesinfektionsmittel die Tenazität. Analog weisen Studien zur quantitativen mikrobiellen Risikobewertung (QMRA) darauf hin, dass das Risiko einer SARS-CoV-2-Infektion über den Übertragungsweg von Flächen gering ist und im Allgemeinen unter 1:10.000 liegt, was bedeutet, dass die Wahrscheinlichkeit einer Infektion bei jedem Kontakt mit einer kontaminierten Oberfläche weniger als 1:10.000 beträgt [152-154].

Unter Berücksichtigung der Übertragungswege und der Umgebungskontamination hat Japan auf die 3 C „closed spaces with poor ventilation", "crowded spaces with many people" und "close contact" fokussiert. Zusätzlich ist eine ausreichende Belüftung in Innenräumen anzustreben, wobei hierfür bisher keine Standards abgeleitet werden können [155]. Die Notwendigkeit der Desinfektion von Oberflächen richtet sich nach der zu erwartenden Kontamination und den Kontaktmöglichkeiten. In Isoliereinheiten für Patienten mit COVID-19 und in Einheiten zur Unterbringung von Verdachtsfällen ist die Flächendesinfektion in Anbetracht der Nachweisbarkeit von SARS-CoV-2 in der gesamten Patientenumgebung und der Tenazität von bis zu 7 d indiziert, obwohl die Infektiosität der Oberflächen offenbar nur gering ist. Aber auch im Haushalt konnte in einer allerdings nur retrospektiven Fragebogen-basierten Studie ermittelt werden, dass Schutzmaske und tägliche Anwendung von Chlor- und Ethanol-basierten Desinfektionsmitteln zur Flächendekontamination bzw. Händedesinfektion das Infektionsrisiko signifikant senken [156]. Santarpia et al. [157] leiten anhand der Datenlage ab, dass im Fall des Verdachts oder bestätigter COVID-19 innerhalb der letzten 24 h im Haushalt auch die Flächen desinfiziert werden sollten.

Tabelle 4: Tenazität von Viren nach Isolierung von Flächen

Erreger	Methode	Ausgangs-inokulum/ applizierte Menge	Tenazität[a]	Quantifizierung /Restvirustiter (b)	Keimträger	Temperatur/ RLF	Bemerkung	Quelle
**Vorwiegende Übertragung durch Kontakt**								
Adenovirus	Elution, Zellkultur	~ 7 lg $CCID_{50}$/KT; Applikation von 0,2 ml	> 12 Wo	nach 1 d 7-8 lg, nach 7 d 6-7 lg, nach 2 Wo 5-7 lg, nach 4 Wo 2,4-7,9 lg, nach 8 Wo 3,4-5,7 lg $CCID_{50}$/KT	Glas/Kunststoff/Porzellan/Edelstahl (je 2,5 cm^2)	25 °C, 37 °C/ 7 %, 55 %, 93 %	Adenovirus (AD) Typ 2; Lagerung im Dunkeln; Nachweisgrenze 1,2 lg; kein Einfluss der Oberfläche; tendenziell geringere Tenazität bei 3 % und 7 % RLF und z.T. bei 37 °C	[158]
	Elution, Zellkultur	2000 plaque-bildende Einheiten (PFU)/KT; Applikation 20 µl	bis 49 d/28-49 d	nach 7 d ~ 20 % Resttiter, nach 14 d ~ 8 %/nach 7 d ~ 7 %, nach 14 d ~ 3%	Kunststoff (Ø 7 mm)/Aluminiumfolie (Ø 6 mm; 120 KT je Material)	22 °C/RL	okulare Laborstämme (AD5, 8, 19) und klinische Isolate (AD8 Cray, AD19 Kowalski, and AD5 McEwen)	[159]
	Elution, Zellkultur	~ 6 lg PFU/20 µl (bei 1 cm^2) bzw. 50 µl (bei 3 cm^2)	15 d; 15 d; 15 d/30 d; 30 d; 15 d/15 d; 15 d; 30 d/ > 30 d; > 30 d; > 30 d auf Aluminium bei fäkaler Belastung und 4 °C/90 %		Aluminium/Porzellan/ Latex/Papier (1 bzw. 3 cm^2)	4 °C/90 %; 20 °C/85 %; 20 °C/50 %	verdünnt in PBS oder 20 %iger Stuhl-Suspension; 3-5 h forcierte Trocknung, dann Lagerung, tendenziell in Stuhl höhere Tenazität	[122]

			sowie 20 °C/85 % > 60 d					
Adenovirus Typ 3	Abschaben, Resus-pension, Zellkultur	~ 7 lg $TCID_{50}$/ml; Applikation von 500 µl	> 9 d	Resttiter nach 24 h/48 h/72 h/6 d/9 d: ~ 6,1 lg/5,8 lg/5,3 lg/5,7 lg/4,2 lg	Polystyrenpetrischale	21-25 °C	angetrocknet in 10 % fetalem Kälberserum; Nachweisgrenze 1,8 lg	[160]
				Resttiter nach 24 h/48 h/72 h/6 d/9 d: ~ 6,8 lg/6,2 lg/6,1 lg/6 lg/5 lg			in Suspension mit 10 % fetalem Kälberserum; Nach-weisgrenze 1,8 lg	
Cytomegalievirus	Elution, Zellkultur	4-6,9 lg PFU/ml Urin, Applikation von 0,2 ml	1-2 h/4-8 h		Baumwolltuch/Plexiglas	25-27 °C/n. b.	Applikation von Urin erkrankter Patienten	[161]
Ebolavirus		4-6 lg $TCID_{50}$; Applikation von 20 µl	bei 4 °C > 50 d auf Glas und Kunststoff (Resttiter 2 lg)	bei 4 °C nach 14 d Reduktion von 6 lg auf 5,5 lg auf Glas und von 5,8 lg auf 3,8 lg auf Kunststoff	Kunststoff/Glas (Ø 7 mm)	4 °C/55 ± 5%	Patientenstamm ZEBOV E718; nach Applikation 30 min Trocknung, Nachweisgrenze 10 $TCID_{50}$/ml; bei RT keine Rückgewinnung von Edelstahl	[162]
	Abspülen mit 150 µl, Zellkultur	7 lg PFU; Applikation von 20 µl auf 1 cm^2	6,2 d; 2,5 d	n.b.	Banknote (1 cm^2)	21 °C/55 %; 32 °C/80 %	nach 1 h Trocknung Exposition in Dunkelheit	[163]
	Elution, Zellkultur	7,3 lg PFU	> 5,9 d	nach 15,4 h Reduktion auf 37 % des Ausgangs-inokulums, nach 5,9 d Reduktion auf 4 lg	Glas/Silikonkautschuk/ lackierte Aluminium-legierung (5 x 5 mm)	20-25 °C/30-40 %	Patientenstamm Zaire	[164]

Hendra-Virus (HeV)		~ 6,25 lg $TCID_{50}$/ml, Applikation von 10 µl	bei 22 °C 60 min, bei 37 °C < 15 min	bei 22 °C: nach 30 min ~ 2,7 lg	nichtporöses Polystyren	22 °C/n. b., 37 °C/n. b.	Nachweisgrenze 126 $TCID_{50}$/ml; bei 37 °C nur geringe Unterschiede zum Nipah-Virus	[165]
Lassavirus		7,1 lg PFU	> 9,7 d	nach 25,3 h Reduktion auf 37 % des Ausgangs-inokulums, nach 9,7 d Reduktion auf 4 lg	Glas/Silikonkautschuk/ lackierte Aluminium-legierung (5 x 5 mm)	20-25 °C/30-40 %	Patientenstamm Josiah	[164]
Marburgvirus		4-7 lg $TCID_{50}$, Applikation von 20 µl	Bei 4 °C > 50 d auf Glas und Kunststoff (Resttiter 2 lg)	bei 4 °C nach 14 d Reduktion von 6 lg auf 5,5 lg auf Glas, von 6 lg auf 3,5 lg auf Kunststoff	Kunststoff/Edelstahl/ Glas (Ø 7 mm)	4 °C/55 ± 5 %	Patientenstamm Lake Victoria	[162]
Nipah-Virus (NiV)		~ 6,25 lg $TCID_{50}$/ml, Applikation von 10 µl	bei 22 °C: nach 30 min ~ 3,7 lg, nach 60 min ~ 2,7 lg	bei 22 °C: nach 30 min ~ 2,7 lg	nichtporöses Polystyren	22 °C/n. b., 37 °C/n. b.	Nachweisgrenze 126 $TCID_{50}$/ml; bei 37 °C nur geringe Unterschiede zwischen beiden Viren	[165]
Sindbis Virus		7,2 lg PFU	> 14,6 d	nach 36,9 h Reduktion auf 37 % des Ausgangsinokulums, nach 14,6 d Reduktion auf 4 lg	Glas/Silikonkautschuk/ lackierte Aluminium-legierung (5 x 5 mm)	20-25 °C/30-40 %	Stamm AR-339 (ATCC VR-1248)	[164]
Vacciniavirus		7 lg $CCID_{50}$/KT, Applikation von 0,2 ml	> 5; > 4; > 5; > 4; > 4; > 4 Wo	2,5; 2,0; 2,0; 2,5; 2,0; 2,0	Glas (2,5 cm^2)	25 °C/96 %; 25 °C/55 %; 25 °C/7 %; 37 °C/96 %; 37 °C/55 %; 37 °C/3 %	Nachweisgrenze 1,5 lg $CCID_{50}$; kein Unterschied in der Tenazität auf Glas, Keramik, Edelstahl und Vinyl-Asbest	[158]

		8 lg PFU/ml	bis 56 d	4,7 ± 4,4 lg	verzinkter Stahl	6-7 °C/1-10 %		[166]
		6-6,5 lg KID_{50}/ml	bis 20 Wo	4,25 lg KID_{50}/ml	Glasröhrchen	6-8 °C/n. b.	getrocknet und zellgebunden	[167]
**Übertragung durch Kontakt, ausgehend vom Gastrointestinaltrakt (+ Surrogatviren)**								
Adenovirus, Typ 40	Elution, Zellkultur	5-5,7 lg IE/KT; Applikation von 20 µl (bei 1 cm^2) bzw. 50 µl (bei 3 cm^2)	> 7 d	Titerabnahme (lg) nach 7 d in PBS 3,5; 3,8/<br>4,5; 3,8	Papier/Porzellan (Papier: 1 cm^2, Porzellan: 3 cm^2)	4 °C/90 ± 5 %;<br>20 °C/90 ± 5 %	verdünnt in PBS oder 20 %iger Stuhlsuspension	[168]
Astrovirus, Serotyp 4	Elution, Zellkultur	5-5,7 lg IE/KT; Applikation von 20 µl (bei 1 cm^2) bzw. 50 µl (bei 3 cm^2)	4 °C: 90 d/60 d; 20 °C: 60 d/7 d	Titerabnahme (lg) N_t/N_0<br>nach 7 d in PBS bei -2; -4/<br>-5; -4	Papier/Porzellan (Papier: 1 cm^2, Porzellan: 3 cm^2)	4 °C/90 ± 5 %; 20 °C/90 ± 5 %	verdünnt in PBS oder 20 %iger Stuhlsuspension	[168]
Coxsackie-Virus	Elution, Zellkultur	6,8 lg $CCID_{50}$/KT; Applikation von 0,2 ml	2 Wo	nach 1 d 5,7 lg bei 55 % RFL; nach 7 d 5 lg bzw. 3 lg bei 55 % RLF und 25 °C bzw. 37 °C; nach 14 d 2 lg	Glas (2,5 cm^2)	25 °C/7 %, 55 %, 96 %; 37 °C/3 %, 55 %, 93 %	Coxsackie-Virus B3; Lagerung im Dunkeln; Nachweisgrenze 1,2 lg; tendenziell geringere Tenazität bei 3 % und 7 % RLF	[158]
	Elution, Zellkultur	6,5 lg $TCID_{50}$/50 µl	bis 6 Wo	4,39 lg 10 $TCID_{50}$/50 (nach 5 d)	Petrischale	20 ± 2 °C/n. b.	Detektionslimit bei 0,5 lg $TCID_{50}$/50 µl	[169]
Echovirus	Zellkultur	max. 300 PFU/10 ml Virussus-pension	168 h; 72 h<br>168 h; 168 h	0,25; n.b.; 0; -2,5 lg N_t/N_0	Zellulose-Ester-Membran-Filter	22 ± 3 °C/> 80 %;<br>22 ± 3 °C/20 %:<br>5 ± 3 °C/> 80 %;		[170]

						5 ± 3 °C/20 %		
Felines Calicivirus	Elution, Zellkultur	9 lg PFU/ml	> 168 h	7 lg N_t/N_0	Laminat/Keramik/Edelstahl	20-24 °C/75-88 %		[171]
	Elution (30 min Vortexen bei 140 Umdrehungen pro min (rpm), Zellkultur	7 lg $TCID_{50}$/ml, Applikation von 20 µl auf 1 cm^2	90 % Reduktion in Virustiter: 0-4 h, 0-4 h, 0-4 h, 12-24 h, 4-8 h, 0-4 h nicht nachweisbar: 8-12 h, 24-48 h, 8-12 h, 48-72 h, 48-72 h, 24	n. b.	Computertastatur/Computermaus/ Messing/Telefontasten/ Telefonhörer/Telefonkabel	n.b.	Messing (Material für Türklinke und Wasserhahn)	[172]
	Elution, Zellkultur	6 lg PFU	bis 15 d; bis 7d/bis 3 d; bis 1 d/bis 7d; bis < 1 d	< 1,30; 1,30/< 3,00; < 3,10/< 0,80; < 3,80	Teppichfasern aus Wolle/Teppichfasern aus Nylon/Glas	25 °C/30 %; 25 °C/70 %	als Surrogat für humanes Norovirus	[173]
Hepatitis A Virus (HAV)	Elution, Zellkultur	6 lg PFU/ml	> 1 Mon	höchster D-Wert[b] (90 % Reduktion) 12 d/8,4 d	Holz/Edelstahl	15 °C, 25 °C, 32 °C, 40 °C/30 %, 50 %, 70 %	Temperatur und RLF haben signifikanten Einfluss auf die Wiederfindungsrate	[174]
		3-4 lg PFU/ml; Applikation von 10 µl	4 h bis > 7 d	Wiederfindungsrate nach 100 h: 5-50 %	Edelstahl	5 °C, 20 °C, 35 °C/25 ± 5 %, 55 ± 5 %, 80 ± 5 %, 95 ± 5 %	Wiederfindungsrate proportional zur Temperatur und Luftfeuchtigkeit (sensibel bei hoher Temperatur und Luftfeuchtigkeit)	[123]

Attachment to: Commission for Hospital Hygiene and Infection Prevention (KRINKO). Hygiene requirements for cleaning and disinfection of surfaces: recommendation of the Commission for Hospital Hygiene and Infection Prevention (KRINKO) at the Robert Koch Institute. GMS Hyg Infect Control. 2023;18:Doc13. DOI: 10.3205/dgkh000468

		5-5,7 lg IE/KT, Applikation von 20 µl (bei 1 cm^2) bzw. 50 µl (bei 3 cm^2)	> 7 d	Titerabnahme (lg) N_t/N_0 nach 7 d in PBS bei -2,1; -2/ -0,5; -0,5	Papier/Porzellan	4 °C, 20 °C/90 ± 5 %	verdünnt in PBS und oder 20 %iger Stuhl-Suspension, KT 1 cm^2 bzw. 3 cm^2; fäkale Belastung hat geringen Einfluss auf Persistenz	[168]
		6,4 lg Genom-kopien/100 µl	bis 90 d	Edelstahl/PVC: nach 4 d bei 23 °C und 4 °C ~ 65/~ 50 % der initialen Beladung<br><br>90 d: Edelstahl 23 °C 0 %; 4 °C 100 %/PVC 23 °C 10 %; 4 °C 70 % der initialen Beladung	Edelstahl/PVC	23 °C/n. b.; 4 °C/n. b.	20 µl auf KT (Ø 1 cm)	[175]
		4,4 lg Genom-kopien/100 µl	8 d	Edelstahl/PVC: nach 4 d bei 23 °C und 4 °C max. 15 % der initialen Beladung; nach 8 d 0-5 %; (nur auf Edelstahl bei 4 °C nach 80 d ~ 5 %)				
	Elution, Zellkultur	~ 6 lg PFU/20 µl (bei 1 cm^2) bzw. 50 µl (bei 3 cm^2)	> 60 d; > 60 d; > 60 d/> 60 d; 30 d; > 60 d/> 60 d; > 60 d; > 60 d/> 30 d; > 30 d; > 30 d		Aluminium/Porzellan/ Latex/Papier (1 bzw. 3 cm^2)	4 °C/90 %; 20 °C/85 %; 20 °C/50 %	verdünnt in PBS oder 20 %iger Stuhl-Suspension; 3-5 h forcierte Trocknung, dann Lagerung	[122]
Escherichia Virus (MS2-Phage)	Elution, Zellkultur	6 lg PFU/ml	> 1 Monat	höchster	Holz/Edelstahl	15 °C, 25 °C, 32 °C, 40	Surrogat für Noroviren;	[174]

				D-Wert[b] (90 % Reduktion) 19,8 d/13,2 d		°C/30 %, 50 %, 70 %	Temperatur und RLF haben signifikanten Einfluss auf Wiederfindungsrate	
Murines Hepatitis Virus und (MHV) Transmissibles Gastroenteritis Virus	Elution, Zellkultur	4-5 lg PFU/10 µl	> 28 d; > 28 d; > 28 d; > 28 d; bis 3 d; bis 14 d; bis 120 h; bis 12 h; < 6 h	Titerreduktion bei 4 °C/20 % nach 28 d 0,5 lg, bei 4 °C/50 % nach 21 d 3,5 lg, bei 4 °C/80 % nach 28 d 3,2 lg; bei 20 °C/20 % nach 28 d 2 lg, bei 20 °C/50 % nach 3 d 2 lg bzw. für MHV nach 5 d 3 lg, bei 20 °C/80 % nach 3 d 1 lg und nach 14 d 3 lg, für MHV nach 5 d 2,2 lg und nach 11 d 5 lg; bei 40 °C/20 % nach 5 d 3,5 lg, für MHV nach 5 d 4,7 lg, bei 40 °C/80 % nach 3h 2,8 lg, für MHV 4,1 lg	Edelstahl (1 cm^2)	4 °C/20 %; 4 °C/50 %; 4 °C/80 %; 20 °C/20 %; 20 °C/50 %; 20 °C/80 %; 40 °C/20 %; 40 °C/50 %; 40 °C/80 %	beide Surrogate für SARS-CoV-1 ähnliche Tenazität; höchste Tenazität bei 4 °C, geringer bei 20 °C und erneut geringer bei 40 °C; RLF von geringerem Einfluss	[124]
Murines Norovirus	Elution, Zellkultur	4-4,5 lg PFU/cm^2	> 120 min außer Kupfer	nach 30 min (trocken): für alle Kupferproben kein Nachweis/Edelstahl 4,1 lg	Kupfer 100 %/95 %/70 %/Edelstahl	RT/n. b.	trocken: Applikation von 1 µl	[176]
				nach 120 min (trocken): Edelstahl 3,1 lg				

				nach 120 min (feucht): nicht nachweisbar/~ 1,2/ ~ 1,9/~ 4 lg			feucht (Applikation von 20 µl, trocknet in 30 min); bei 4 °C verzögerte Tenazität	
Poliovirus Typ 1	Abstrich, Zellkultur	4,4 lg PFU/ml	> 90 min	nach 90 min Reduktion von 4,4 lg auf 2,6 lg	Arbeitsplatte (10x25 cm)	n.b.	suspendiert in 10 % Stuhllösung, Antrocknung	[177]
	Elution, Zellkultur	~ 6 lg PFU/20 µl (bei 1 cm^2) bzw. 50 µl (bei 3 cm^2)	7 d; 2 d; 3 d/> 60 d; 30 d; 1 d/30 d; 15 d; 30 d/> 30 d; > 30 d; > 30 d		Aluminium/Porzellan/ Latex/Papier (1 bzw. 3 cm^2)	4 °C/90 %; 20 °C/85 %; 20 °C/50 %	Teststamm LSc 2a; verdünnt in PBS oder 20 %iger Stuhl-Suspension; 3-5 h forcierte Trocknung, dann Lagerung	[122]
		max. 300 PFU/10 ml Virussus-pension	168 h; 72 h 168 h; 168 h	0,25; n.b.; 0; -2,5 lg N_t/N_0	Zellulose-Ester-Membran-Filter	22 ± 3 °C/> 80 %; 22 ± 3 °C/20 %: 5 ± 3 °C/> 80 %; 5 ± 3 °C/20 %		[170]
		~ 12 lg PFU/10 µl	>3 Wo auf allen Ober-flächen	90 % Reduktion nach 2,4 d/3,4 d/2,6 d; 99 % Reduktion nach 5,2 d/7,4 d/5,9 d	Stahl/Baumwolle/Kunst-stoff (25 cm^2)	18-20 °C/40-60 %	Filtrierung vor Beprobung	[178]
		3-4 lg PFU; Applikation von 10 µl	bis 4 h; bis 12 h	4 h: 12 %; 12 h: 28 % des Inokulums	Edelstahl	20 °C/95 ± 5 %	überlebt bei extrem hoher RLF am längsten	[123]
Poliovirus Typ 2	Elution, Zellkultur	8,1 lg PFU/KT; Applikation von 0,2 ml	bis 8 Wo bei 7 % RLF; bis 4 Wo bei 25 °C und 7 % sowie	nach 1 d 7 lg bzw. 6 lg bei 55 % RLF und 25 °C bzw. 37 °C, nach 7 d 5 lg bzw. <	Glas (2,5 cm^2)	25 °C/7 %, 55 %, 96 %; 37 °C/3 %, 55 %, 93 %	Lagerung im Dunkeln; Nachweisgrenze 1 lg tendenziell höhere	[158]

			96 % RLF sowie bei 37 °C und 3 % RLF	1,2 lg bei 25 °C bzw. 37 °C und 55 % RLF; nach 14 d bei 25 °C 5 lg, 3 lg bzw. 6 lg bei 7 %, 55 % und 96 % RLF			Tenazität bei 3 % und 7 % RLF	
	Elution, Zellkultur	5-5,7 lg IE/KT; Applikation von 20 µl (bei 1 cm^2) bzw. 50 µl (bei 3 cm^2)	> 7 d	Titerabnahme (lg) N_t/N_0 nach 7 d in PBS bei -3,6; -4,3/ -4; -4,4	Papier/Porzellan (1 cm^2 bzw. 3 cm^2)	4 °C, 20 °C/90 ± 5 %	verdünnt in PBS und oder 20 %iger Stuhl-Suspension	[168]
Rotavirus	Elution, Zellkultur	~ 6 lg PFU/20 µl (bei 1 cm^2) bzw. 50 µl (bei 3 cm^2)	> 60 d; > 60 d; > 60 d/> 60 d; > 60 d; > 60 d/> 60 d; > 60 d; > 60 d		Aluminium/Porzellan/ Latex/Papier (1 bzw. 3 cm^2)	4 °C/90 %; 20 °C/85 %; 20 °C/50 %	verdünnt in PBS (oder 20 %iger Stuhl-Suspension); 3-5 h forcierte Trocknung, dann Lagerung	[122]
	Abstrich, Zellkultur	3-4 lg PFU/ml	bis 90 min	~ 1 lg PFU/ml	Arbeitsplatte (10x25 cm)	n.b.	suspendiert in 10 % Stuhllösung, Antrocknung	[177]
	Elution mit US 10 min, Zellkultur	7 lg PFU/ml	bei 22 °C und 4 °C > 10 d, bei 36 °C auf nicht poröser Fläche 6 d	bei 22 °C und 25 % und 50 % RLF nach 10 d ~ 10 % der Ausgangsmenge infektiös, bei 85 % RLF > 99,9 % Reduktion nach 48 h; bei 4 °C tendenziell höhere Tenazität	Glas/Edelstahl/glatter Kunststoff/rauer Kunststoff (1 cm Durchmesser); bei 4°C nur Edelstahl	22 ± 2 °C/25 ± 5 %, 50 ± 5 %, 85 ± 5 %; 4 °C/50 ± 5 %, 85 ± 5 %; 36 ± 1 °C 25 ± 5 %, 50 ± 5 %	Viren in Stuhl suspendiert; signifikanter Einfluss von RLF, aber nicht von Temperatur	[179]
	Elution, Zellkultur	5-5,7 lg IE/KT; Applikation von 20 µl (bei	> 7 d	Titerabnahme nach 7 d in PBS um 1,5; 1,2/1; 1,2 lg	Papier/Porzellan (1 cm^2 bzw. 3 cm^2)	4 °C, 20 °C/90 ± 5 %	verdünnt in PBS und oder 20 %iger Stuhl-Suspension; fäkale	[168]

		1 cm^2) bzw. 50 µl (bei 3 cm^2)					Belastung hat geringen Einfluss auf Persistenz	
Tulane Virus (Rhesus enteric calicivirus)	Elution, Zellkultur	4,7 lg PFU/ml, Applikation von 50 µl	> 28 d/> 14 d/> 14 d	Reduktion nach 14 d um 0,5/07/1 lg, kalkulierter D-Wert[b] für Acryl 18,8 ± 0,34 d. für Edelstahl 13,3 ± 0,36 d	Wasser/Acryl/Edelstahl (7,6 cm^2)	RT/16-22 %	als Surrogat für humanes Norovirus; Nachweisgrenze 5 lg PFU/ml; Exposition bei Tageslicht; zwischen 1-7 d kein signifikanter Titerverlust auf Acryl und Edelstahl	[180]
**respiratorische und/oder aerogene Übertragung (+ Surrogatviren)**								
Endemische Humane Coronaviren	Elution mit Ultraschall, Zellkultur	5,7 lg $TCID_{50}$/ml, Applikation von 10 µl	HCoV-229E: 12 h, 12 h, 6 h; HCoV-OC43: 3 h, 1 h, 1 h	Restinfektiosität 10-20 %	Aluminium/sterile Schwämme/Operations-handschuhe aus Latex (je 1 cm^2)	21 °C/55-70 %	Stämme HCoV-229E und HCoV-OC43; nach Kontamination Trocknung	[181]
	Elution, Zellkultur	3 lg PFU/20 µl	3 d/5 d/≤40min/120 min/30 min	Restinfektiosität auf PVC, Keramik, Glas und Stahl ≤ 1 lg PFU	Silikongummi/PVC, Keramik, Glas, Stahl/Messing/70 % Kupfer/90 % Kupfer (je 1 cm^2)	21 °C/30-40 %	Stamm HCoV-229E	[182]
	Abschaben, Resus-pension, Zellkultur	~ 7 lg $TCID_{50}$/ml; Applikation von 500 µl	bis 48 h	nach 48 h: 2 lg	Polystyrenpetrischale	21-25 °C	Stamm HCoV-229E; angetrocknet in 10 % fetalem Kälberserum; Nachweisgrenze 1,8 lg	[160]
			bis 8 d	nach 8 d: 2 lg			Stamm HCoV-229E; in Suspension mit 10 % fetalem Kälberserum; Nachweisgrenze 1,8 lg	

Influenza A Viren	Zellkultur-basierter ELISA	3,1 lg $TCID_{50}$/ KT (A/NC-H1N1); 4,8 lg $TCID_{50}$/ KT (A/Br-H1N1)	bis 168 d	A/NC-H1N1: -1,22; -0,32; -0,28 lg $TCID_{50}$/ml A/Br-H1N1: -1,30; -1,32; -2,03 lg $TCID_{50}$/ml	Edelstahl	18 °C/20 %; 25 °C/35 %; 25 °C/55 %	Stämme A/Brisbane/59/2007 (A/Br-H1N1), A/New Caledonia/20/1999 (A/NC-H1N1), suspendiert in fetalem Rinderserum	[183]
	Elution, Zellkultur	5 lg $TCID_{50}$/50 µl	bis 5 d	~ 0,5 lg $TCID_{50}$/50 µl	Petrischalendeckel	RT		[169]
		4-6 lg PFU/ml; 10 µl mit 0,3 % BSA	7 d/14 d/14 d	99 % Reduktion nach 7,3 d/17,7 h/34,3 h	Edelstahl/Baumwolle/ Mikrofaser	19,5-19,7 °C/10,2-55,6 %	Prüfstämme Influenza A/Bris/59/07/H1N1 (NIBSC), Influenza A/Cal/4/09/H1N1 (NIBSC), Influenza A/Cal/7/09/H1N1 (NIBSC), Influenza A/PR/8/34/H1N1, Influenza A/Sol/3/06/H1N1 (NIBSC), Austrocknung; Licht hatte keinen Einfluss, Höhe der Ausgangsinokula hat Einfluss auf Tenazität	[184]
		3-4 lg $TCID_{50}$/0,1 ml	48 h, 72 h, 24 h, 24 h, 12 h, 24h	nach 48 h Kunststoff/Edelstahl : ~ 1 lg $TCID_{50}$/0,1 ml	Kunststoff/Edelstahl/ Magazine/Baumwolle/ Papiertaschentuch/Stoff-taschentuch	27,8-28,3 °C/35-40 %	Detektionslimit bei 0,5 lg $TCID_{50}$/0,1 ml	[185]
		6 lg PFU/ml, Applikation von 10 µl	2-9 h	Plaque Assay: nach 9 h ~ 1,4 lg PFU/ml	Telefonhörer/Holz/Ta-statur/Edelstahl/Spül-tuch	RT/23-24 %	Stämme A/PuertoRico/8/34 (PR8) und	[186]

							A/Cambridge/AHO4/ 2009 (pandemic H1N1)	
		6 lg $TCID_{50}$/ml; Applikation von 5 µl	< 4 h	50 % Reduktionsrate nicht kalkulierbar	Stahl/Kunststoff	20 °C/40 %; 30 °C/30 %; 30 °C/80 %	Stamm A/Mexico/4108/2009 (H1N1)	[187]
Influenza B Virus		4 lg $TCID_{50}$/0,1 ml	48 h/48 h, 8 h/12 h/8 h/8 h	nach 48 h Kunststoff/Edelstahl : ~ < 1 lg $TCID_{50}$/0,1 ml	Kunststoff/Edelstahl/ Magazine/Baumwolle/ Papiertaschentuch/ Stofftaschentuch	26,7-28,9 °C/55-56 %		[185]
Middle East Respiratory Syndrome Corona-Virus (MERS-CoV)	Elution, Zellkultur	6 lg $TCID_{50}$/ml; Applikation von 5 µl	bis 72 h; bis 48 h; bis 24 h		Stahl/Kunststoff	20 °C/40 %; 30 °C/30 %; 30 °C/80 %	Stamm HCoV-EMC/2012	[187]
Parainfluenza-virus		3,2 lg $TCID_{50}$/ml	feucht: bis zu 10 h; trocken: bis max. 4 h/feucht: bis 8 h; trocken bis 4 h	Recovery Rate: 20 % nach 10 h/50 % nach 8 h	Edelstahl/Laminat	22 °C/n. b.		[188]
Respiratory Syncytial-Virus		5 lg $TCID_{50}$/ml	8 h; ~ 2,5 h; ~ 5,3 h; 1 h; 1 h	Laminat nach 7 h Reduktion um 4,5 lg	Laminat/Textil (Baumwoll-Polyester)/Gummihand-schuh/Papiertuch/Hände	22-25 °C/35-50 %		[189]
Rhinovirus Typ 14		7 lg PFU/10 µl	bis 25 h	Halbwertszeit ($TCID_{50}$): 0,24 h; 0,55 h; 1,42 h	Edelstahl	20 °C/20 ± 5 %; 50 ± 5 %; 80 ± 5 %	Testung in Nasalsekret, Tryptose -Phosphat-Bouillon und Muzin	[190]
Rhinovirus Typ 2		2 lg PFU/ml	bis 4 d	nach 3 d ~ 0,6 lg	Edelstahl	n. b.	2-10 min Lufttrocknung	[191]

SARS-CoV-1		6 lg TCID$_{50}$/ml; Applikation von 300 µl	4 d/4 d/4 d/5 d/5 d/5 d	nur einige PFU	Holz/Glas/Papier/Metall/ Kleidung/Filterpapier (je 1,5 cm^2)	20 °C/n.b.	Stamm P9	[192]
	Resus-pension (100 µl), Zellkultur	7 lg TCID$_{50}$/ml	bis 28 d	Reduktion des Virustiters um ~ 5 lg	Kunststoff	22-25 °C/ 40-50 %		[193]
	Elution, Zellkultur	3,4 lg TCID$_{50}$/ml	auf Kunststoff bis 72 h	Titerreduktion auf 0,7 bzw. 0,6 lg TCID$_{50}$/ml auf Kunststoff nach 72 h; auf Edelstahl nach 48; auf Pappe nach 24 h; auf Kupfer nach 4 h keine Rekultivierung	Kunststoff/Edelstahl/ Pappe/Kupfer	21-23 °C/40 %	Tor 2 (AY274119.3); im Aerosol nach 3 h Reduktion von 4,6 lg auf 3,5 lg; Nachweisgrenze 0,7 lg/ml (auf Kupfer 1,2 lg/ml)	[129]
		4 lg TCID$_{50}$/ml	< 5 min/3 h/24 h	n. b.	Papier	RT/n.b.	Stamm GVU 6109; KT 1 cm^2	[194]
		5 lg TCID$_{50}$/ml	5 min/1 h/24 h		Baumwolle			
		6 lg TCID$_{50}$/ml	1 h/24 h/2d		undurchlässige Einwegkittel			
	Re-suspension (100 µl), Zellkultur	7 lg TCID$_{50}$/ml		nach 5 d, 7 d bzw. 13 d Verlust von 1 lg, 2,4 lg bzw. 4,7 lg	Kunststoff (24- well Platte)	22-25 °C/40-50 %	Stamm HKU 39849	[193]
	Abschaben, Resus-pension, Zellkultur	~ 7 lg TCID$_{50}$/ml; Applikation von 500 µl	> 9 d	Resttiter nach 24 h/48 h/72 h/6 d/9d: ~ 5 lg/4,8 lg/4 lg/3,7 lg/2 lg	Polystyrenpetrischale	21-25 °C	klinisches Isolat, Stamm FFM-1; angetrocknet in 10 % fetalem Kälberserum, Nachweisgrenze 1,8 lg	[160]

				Resttiter nach 24 h/48 h/72 h/6 d/9d: 6,5 lg/6,3 lg/6,2lg/6lg/5,2 lg			klinisches Isolat, Stamm FFM-1; in Suspension mit 10 % fetalem Kälberserum, Nachweisgrenze 1,8 lg, Nachweisgrenze 1,8 lg	
SARS-CoV-2	Elution, Zellkultur	5,5 lg $TCID_{50}$/ml; Applikation von 10 µl, 1 h angetrocknet	bei 20 °C bis 28 d, bei Baumwolle bis 7 d nachweisbar, bei 40 °C < 24 h nachweis-bar	nach 14 d bei 20 °C $TCID_{50}$/ml ~ 4,2 lg/4 lg/3,2 lg/3,8 lg/unter Nachweisgrenze/3,1 lg; D-Werte[b] bei 20 °C 6 (1,8[a] d)/6,9 (2,1 d)/9,1 (2,7 d)/6,3 (1,9 d) 5,6 (1,7 d)/6,3 (1,9 d); bei 30 °C 1,7 (12,6 h)/2 (14,7 h)/4,3 (32,7 h)/1,5 (10,5 h)/1,7 (11 h)/1,4 (10,2 h); bei 40 °C 4,9 (1,5 h)/4,8 (1,4 h)/5,4 (1,6 h)/6,7(2h)/-/9,9 (3h)	Edelstahl/Papierbank-note/Polymerbank-note/Glas/Baumwolle/ Vinyl (je 1-1,5 cm^2)	20, 30, 40 °C/50 %	Stamm Australia/SA01/2020) ; Nachweisgrenze 0,8 lg $TCID_{50}$; [a] Zeitdauer (d) für 50 %ige Reduktion	[132]
		7,9 lg $TCID_{50}$/ml; Applikation von 10 µl	bis 21 d, < 24 h auf Baumwolle	Rückgewinnung nach 7 d ~ 2,7 lg/2 lg/2,8 lg/nicht nachweisbar (n.n.)/2,3 lg/2,3 lg/1,1 lg/n.n.	Edelstahl/Gesichtsschild/ Nitrilhand-schuh/Chemikalien-handschuh/N95 Maske/N100 Maske/Tyvek-Schutzanzug/Baumwolle	20 °C/35-40 %	Stamm hCoV-19/Canada/ON-VIDO-01/2020; Testung bei Belastung mit BSA, Muzin und Trypton; nach Applikation von 10 µl Lufttrocknung 1	[131]

					(Edelstahl 1 cm^2, übrige 1,4 cm^2)		h; Nachweisgrenze ~ 0,6 $TCID_{50}$	
	Zellkultur	3,6 lg $TCID_{50}$/ml	auf Kunststoff bis 72 h	Titerreduktion auf 0,8 lg $TCID_{50}$/ml auf Kunststoff nach 72 h; auf Edelstahl nach 48 h; auf Pappe nach 24 h; auf Kupfer nach 4 h keine Rekultivierung; Reduktion um 50 % nach 5,6 h auf Stahl, 6,8 h auf Kunststoff , 3,2 h auf Pappe und 0,5 h auf Kupfer	Kunststoff/Edelstahl/ Pappe/Kupfer	21-23 °C/40 %	Stamm nCoV-WA1-2020 (MN985325.1); im Aerosol nach 3 h Reduktion von 5,5 lg auf 2,7 lg	[129]
	Elution, Zellkultur	7,8 lg $TCID_{50}$/ml, Applikation von 5 µl	Papier/Ta-schentuch: bis 3 h, Holz/Kleidung : bis 2 d, Glas/Geld-schein: bis 4 d, Edelstahl/ Kunststoff: bis 7 d, OP-Maske: 7 d	Wiederfindungsrate	Papier/Taschentuch/Holz /Kleidung/Glas/Geld-schein/Edelstahl/Kunst-stoff/OP-Maske innere Schicht/OP-Maske äußere Schicht (je 1 cm^2)	22 °C/65 %	Detektionslimit bei 100 $TCID_{50}$/ml,	[130]
		6,2 ± 5,9 lg $TCID_{50}$/ml Speichel, Applikation von 5 µl ml	> 13 min bei 0,3 Watt (W)/cm^2	linearer Abfall; Verlust von 90 % alle 6,8 min, 8 min bzw. 12,8 min bei 1,6; 0,7 bzw. 0,3	Edelstahl	20 ± 4 °C/19 ± 5 %	Stamm USA-WA1/2020; nach Applikation 30 min Lufttrocknung bei 24 ± 1 °C und 29 ± 2 %	[133]

				W/cm²; im Dunkeln Verlust zwischen 0-0,15 $TCID_{50}$/min			RLF Einwirkung von simuliertem Sonnenlicht (Xenonlampe 280-400 Nanometer (nm) UV); Wiederfindungsrate im Speichel 2,8 ± 0,1 lg vor und 3,0 ± 0,2 lg nach 30 min Trocknung	
		6,5 lg $TCID_{50}$/ml; Applikation von 10 µl	bis 20 min im Sonnenlicht	Absterberate (%)/min: nach 7 h im Dunkeln 1,25 %/min bei 20 °C; 0,58 %/min bei 35 °C; bei 79 W/cm² 22,8 %/min bei 20 °C; 25,5 %/min bei 35 °C; bei 35 °C und 79 W/cm² Virus für 20 min nachweis-bar; bei 442 W/cm² nach 5 min kein Virus-nachweis	Edelstahl		Sonnenlichtsimulation mit Xenonlampe; 79 W/cm² entspricht bewölktem Wetter; Wiederfindung nach 30 min Trocknung 3,2 lg; Belastung mit 1 % BSA + 1 % Hefeextrakt erhöht Tenazität gering aber signifikant (106 %/min statt 94 %/min)	[195]
	Zellkultur	~ 2,8 lg $TCID_{50}$/ml	> 18,6 h	bei 24 °C Reduktion der $TCID_{50}$ um 50 % zwischen 6,3 h und 18,6 h mit steigender RLF, aber Reduktion auf 1-8,9 h bei 35 °C	Edelstahl/Kunststoff/ Nitrilhandschuh	24-35 °C/20-80 %	Virusverdünnung mit simuliertem Speichel 1:10; Nachweisgrenze 0,2 lg $TCID_{50}$/ml; Tropfengröße von 1-50 µl und	[142]

							Oberflächenmaterial waren ohne Einfluss; bei Erhöhung von Temperatur oder RLF reduzierte Tenazität	
**Sexuelle Übertragung**								
Herpes simplex-Virus Typ 1	Elution, Zellkultur	7,9 $TCID_{50}$/0,1 ml; Applikation auf 0,5 cm^2	> 2 h	nach 30 min kein Titerverlust, zwischen 30 und 60 min ~ 1 lg Verlust; nach 2 h weitere 0,2 lg Verlust, Virusisolation nach Fingerkontakt über 2 h Periode	Kunststofftürklinke/ Chromwasserhahn	23-27 °C/n. b.	Einbettung in Zellkulturmedium, kein Unterschied zwischen beiden Oberflächen	[196]
			2 h	nach 30 min kein Titerverlust, zwischen 30 und 60 min ~ 2 lg Verlust; nach 2 h weitere 0,7 lg Verlust, Virusisolation nach Fingerkontakt über 2 h Periode; bei Einbettung in Wasser nach 30 min ~ 1 lg Verlust, nach 60 min ~ 2 lg Verlust, nach 2 h ~ 3 lg Verlust		21-25 °C	nach Einbettung in Speichel kein Unterschied zwischen beiden Oberflächen	[197]

		5,6 lg PFU/KT; Applikation von 0,2 ml	1 d unter allen Bedingungen, 8 Wo bei 3 % bzw. 7 %	nach 1 d 4 lg bei 55 % RLF, nach 7 und 14 d 3,4 lg bei 3 bzw. 7 % RLF, nach 6 und 8 Wo ~ 2 lg bei 3 bzw. 7 % RLF	Glas (2,5 cm^2)	25 °C/7 %, 55 %, 96 %; 37 °C/3 %, 55 %, 93 %	Lagerung im Dunkeln; Nachweisgrenze 1 lg; tendenziell höhere Tenazität bei 3 % und 7 % RLF	[158]
	Abschaben, Elution, Zellkultur	~ 7 lg $TCID_{50}$/ml; Applikation von 500 µl	> 9 d	Resttiter nach 24 h/48 h/72 h/6 d/9 d: ~ 4,5 lg/2,7 lg/2,3 lg/2,3 lg/1,9 lg	Polystyrenpetrischale	21-25 °C/n. b.	angetrocknet in 10 % fetalem Kälberserum; Nachweisgrenze 1,8 lg	[160]
			bis 3 d	Resttiter nach 24 h/48 h/72 h/6 d/9 d: ~ 6,5 lg/6,8 lg/6,1 lg/5,8 lg/6,1 lg			in Suspension mit 10 % fetalem Kälberserum; Nachweisgrenze 1,8 lg	
Herpes simplex-Virus Typ 2	Elution, Zellkultur	3,9-6,9 lg PFU/ml (Urin)	bis 4,5 h	2,9 lg $TCID_{50}$/ml	Polystyrol	37-40 °C/hohe RLF		[198]
Humanes Immundefizienz-Virus (HIV)	Infektiositätsassay (T-Lymphozyten)/Messung der Reverse Transkriptase Aktivität	Flüssiginokulum 128000 Zerfälle pro Minute (cpm)/ml Reverse Transkriptase; Trockeninokulum: 25000 cpm/ml Reverse Transkriptase	flüssig > 20 d; trocken ~ 10 d Überleben cpm/ml (Konzentration der Reverse Transkriptase)	zwischen 0 d ,2 d und 4 d kein signifikanter Unterschied, nach 7 d leichter Abfall	versiegeltes Reagenzröhrchen/Petrischale	20-22 °C	Virus widerstandsfähig bei RT (sowohl trocken als auch in feuchtem Medium)	[199]

Papillomvirus	Zellkultur	unterschied-liche Ausgangs-inokula (~ 100-434 Fokusbildende Einheiten	bis 7 d (auch unter trockenen Bedingungen)	Fokusbildende Einheiten: 34±4	Rohr	RT/n. b.	genitaler HPV-16 und boviner PV-1; Virusinaktivierung unabhängig von Ausgangsinokula	[200]
**Übertragung durch Blut**								
Eastern Equine Encephalo-myelitis-Virus	n.b.	*in vivo*	Nachweis bis 6 d		Federkiel			[201]
Gelbfiebervirus	Zellkultur	8,3 lg PFU/ml	90 d	Resttiter nach 0/2/4/7/14/21/28 und 40 d: 8,3/8,7/8,3/8/7,5/6, 5/5,5/5/< 1,7/< 1,7 lg	Filterpapier (Ø 5,5 cm)	RT		[202]
Hepatitis B Virus (HBV)	Radio-immuno-assay (RIA) und HBV-Infektion nach intra-venöser Applikation des re-hydrierten Inokulums beim Schim-pansen	n.b./0,1 ml HBsAg positives Plasma	1 Wo		silanisiertes Röhrchen	25 °C/42 %	Exposition in Dunkelheit	[203]
	RIA	n.b./0,1 ml HBV-positives Blut	> 2 Wo	Verlust der Antigenaktivität nach 14 d 15-20 %	Edelstahl/Wattestäbchen	25 ± 1 °C/42 ± 2 %		[204]

Hepatitis-C-Virus (HCV)	Elution, Zellkultur	n.b./33 µl infiziertes Spender-plasmas a) Niedrigtiter-Inokulum: 4 lg IE/ml); b) Hochtiter-Inokulum: 6 lg IE/ml)	a) und b) > 40 d bei 4 °C/RT	a) und b) nach 40 d ~ 100 % HCV-positive Spots bei 4 °C/RT<br>a) > 1000 relative Luciferase Aktivität (RLA)<br>b) > 100.000 RLA nach 20 d bei 4 °C/RT	Well-Platten	4 °C,RT, 37 °C/n. b.	je niedriger die Temperatur, desto länger infektiös	[205]
	Zellkultur	~ 4,75 lg $TCID_{50}$/ml	> 7 d (ohne Serumzusatz), mit Serumzu-satz bis 6 d	nach 7 d ~ 1,5 lg	Edelstahl	RT/n.b.		[206]
Venezuelan equine encephalitis virus		n.b.	7 d/7 d/11,4 d	Reduktion um 4 lg	Gummi/Aluminium/Glas	20-25 °C/30-40 %		[207]
		9,8 lg PFU/ml	40 d	nach 0/2/4/7/14/21/28 und 40 d: 8/7,2/6,3/6/4,8/3,8 /3 und < 1,7 lg, nach 60 d n.n.	Filterpapier (Ø 5,5 cm)	RT/n.b.		[202]
		6,5 lg PFU	> 9,7 d	nach 42,7 h Reduktion auf 37 % des Ausgangs-inokulums, nach 11,4 d Reduktion auf 4 lg	Glas/Silikonkautschuk/ lackierte Aluminium-legierung (5 x 5 mm)	20-25 °C/30-40 %	Patientenstamm Trinidad donkey	[164]
West-Nil-Virus		9 lg PFU/ml	60 d	Resttiter nach 0/2/4/7/14/21/2 8/40/60 und 90 d: 6,5/6,0/6,3/5,8/5,0/	Filterpapier (Ø 5,5 cm)	RT/n. b.		[202]

				5,3/3,7/1,8/< 1,7 lg, nach 90 d n.n.				

[a] > bedeutet der letzte Messzeitpunkt [b] D-Wert = Zeit, in der der Virustiter um 1 lg reduziert wird

2.2 Infektiöse Dosis

Vor allem für Erreger mit geringer Infektionsdosis ist das Infektionsrisiko nach Akquirierung aus der Umgebung gegeben (**Tab. 5**). Die Angaben beziehen sich auf die Aufnahme über den Gastrointestinaltrakt, nicht nach Exposition von Schleimhäuten oder Wunden durch Kontakt oder aerogen nach Aufwirbelung von Flächen. Es ist zu berücksichtigen, dass abhängig von der Abwehrlage, z.B. bei Immunsuppression, Neonaten und Stoffwechselerkrankungen, die infektiöse Dosis geringer sein kann.

Tabelle 5: Infektiöse Dosis ausgewählter Krankheitserreger nach oraler Aufnahme

Infektionsdosis	Erreger	Quelle
1-100 infektiöse Viruspartikel bzw. KbE	Noro-, Rotavirus, EHEC, ETEC, *C. difficile*, MRSA	[25, 136, 208-213]
$< 10^3$ KbE	VRE, *Acinetobacter* spp., *Klebsiella* spp., *C. jejuni*	[213, 214]
$\geq 10^3$ KbE	*Salmonella enteritidis*	[215]
$> 10^5$ KbE	*E. coli, S. aureus*	[216]

2.3 Biofilmbildung

Im Biofilm ist die Persistenz deutlich erhöht und die Empfindlichkeit gegen antimikrobielle Wirkstoffe nimmt um das 10 bis > 1000-fache ab [217, 218]. Besonders Gram-negative Erreger, die sich im feuchten Milieu vermehren, sind zur raschen Biofilmbildung befähigt. Aber auch auf trockenen Krankenhausoberflächen wurden Biofilme nachgewiesen, in denen vegetative Bakterien wochen- bis monatelang (oder länger) auf Oberflächen überleben können [218]. Auf häufig berührten patientennahen Flächen auf Intensivtherapiestationen (ITS) wurden durchschnittlich 10^4 Bakterien/cm^2 nachgewiesen, darunter *Enterococcus faecium, S. aureus, Klebsiella pneumoniae, A. baumannii, Pseudomonas (P.) aeruginosa* und *Enterobacter* spp., wobei auf allen Oberflächen Biofilme nachweisbar waren [219]. Zusätzlich ist zu beachten, dass Mikroorganismen im Biofilm eine Toleranz gegenüber Desinfektionsmitteln aufweisen, die aus der Lebensform im Biofilm hervorgeht und verschwindet, wenn sie herausgelöst werden. Sie sollte nicht mit Resistenz verwechselt werden, die genetisch determiniert ist.

Die Fähigkeit von Reinigungs- und Desinfektionsmitteln, Biofilme zu zerstören, die Einführung neuer Oberflächenmaterialien, die die Bildung von Biofilmen hemmen und der Einsatz probiotischer Reinigungsmittel, die aufgrund der kompetitiven Exklusion die Vermehrung von Krankheitserregern verhindern bzw. eindämmen [220], könnten in der Zukunft eine wachsende Rolle spielen [218]. Derzeit dominieren regelmäßige Flächenreinigung und ggf. desinfizierende Flächenreinigung als Maßnahmen zur Verhinderung der Anhaftung und Vermehrung mit nachfolgender Biofilmbildung auf Flächen.

Gleichzeitig sollen Feuchtreservoire eliminiert werden, da insbesondere an Wasser adaptierte Mikroorganismen wie *P. aeruginosa, aber auch E. coli* O 157 und *Bacillus (B.) atrophaeus* subsp. *globigii* zur Biofilmbildung befähigt sind [221]. Das ist insbesondere in Sanitärbereichen wichtig.

3 Toxikologische und ökotoxikologische Merkmale mikrobizider Wirkstoffe

Bei der Bewertung der Risiken (**Tab. 6**) wurden tierexperimentelle Daten zur mutagenen, karzinogenen und teratogenen Potenz nur dann berücksichtigt, wenn sich trotz sachgerechter Anwendung, z.B. aufgrund möglicher inhalativer Exposition, Risiken ergeben könnten. Die Zusammenstellung soll eine orientierende Hilfestellung für die Auswahl von Desinfektionsmitteln unter dem Gesichtspunkt der Risiken für den Menschen, insbesondere für die u. U. ein Berufsleben lang exponierten Mitarbeiter, und für die Umwelt geben. Sind in einem Produkt mehrere Wirkstoffe oder spezielle Hilfs-/Beistoffe enthalten, können dadurch toxikologische Eigenschaften beeinflusst werden, was die Bewertung des gesamten Endprodukts erforderlich macht.

Aufgrund des vom Bundesumweltamt [222] für 2-Propanol in der Innenraumluft veröffentlichten Richtwerts II (Gefahrenwert) von 45 mg/Kubikmeter (m^3) und des Richtwerts I (Vorsorgewert) von 22 mg/m^3 soll folgende Anmerkung gegeben werden: In Schulen und Kindergärten wurde als Median für 2-Propanol 14 Mikrogramm (µg)/m^3 gemessen, ohne dass dort 2-Propanol zur Desinfektion eingesetzt wird. Nach Neubau und Renovierung können die Werte bis etwa 700 µg/m^3 erreichen. In Operationseinheiten wurde im Vereinigten Königreich (UK) als Median 8 mg/m^3 gemessen [223]. Solange 2-Propanol nur zur Händedesinfektion oder kleinflächig patientennah eingesetzt wird, ist die Einhaltung des Richtwerts anzunehmen. Da die Geruchsschwelle bei 65 mg/m^3 liegt [224], sollte bei auffälliger andauernder olfaktorischer Wahrnehmung der Einsatz von 2-Propanol überprüft werden.

Attachment to: Commission for Hospital Hygiene and Infection Prevention (KRINKO). Hygiene requirements for cleaning and disinfection of surfaces: recommendation of the Commission for Hospital Hygiene and Infection Prevention (KRINKO) at the Robert Koch Institute. GMS Hyg Infect Control. 2023;18:Doc13. DOI: 10.3205/dgkh000468

Tabelle 6: Toxikologische und ökotoxikologische Merkmale für mikrobizide Wirkstoffe bzw. Stoffklassen (Daten aus Kramer u. Assadian 2008) [225]

Wirkstoff	Rückstände auf Oberflächen	Toxikologische Eigenschaften[2]	Mögliche Nebenwirkungen trotz sachgerechter Anwendung wegen Flüchtigkeit[2]	Biologische Abbaubarkeit
**Kurzkettige aliphatische Alkohole**	nach Lufttrocknung keine Rückstände	toxikologisch unbedenklich		rasch und vollständig
**Formaldehyd**	gering	mäßig toxisch[1], stark schleimhautreizend, hohe Sensibilisierungspotenz, abhängig vom Testmodell mutagen, karzinogen, neurotoxisch, aufgrund epidemiologischer Daten Einstufung durch die IARC als humankanzerogen Kategorie 1B	Allergie; da der Innenraumleitwert der WHO von 0,1 mg/m³ bei der Flächendesinfektion überschritten wird, ist bei lebenslanger Exposition am Arbeitsplatz ein gering erhöhtes Krebsrisiko nicht auszuschließen	rasch und vollständig
**Glutaral**	gering	Reizung der Atemwege, dermale Resorption, Kontaktallergen, Augenreizung, Rhinitis	Asthma, Kopfschmerz, Augenreizung, Rhinitis	rasch und vollständig
**Glyoxal**	gering	dermale Resorption, Kontaktallergen	Keine Hinweise	rasch und vollständig
**Aliphatische Carbonsäuren (Ameisensäure, Milchsäure)**	gering	toxikologisch unbedenklich		rasch und vollständig
**Peroxide: Wasserstoff-peroxid, Kalium-peroxomonosulfat**	rascher Zerfall	schwach toxisch[2], keine Sensibilisierung, hohe inhalative Toxizität bei Verneblung	unbedenklich	rasch und vollständig

**Peressigsäure**	rascher Zerfall	keine Sensibilisierung, neurotoxisch, Verdacht auf karzinogene Wirkung (Kategorie 3[1])	bei täglicher großflächiger Anwendung Symptome analog sick building syndrome möglich	rasch und vollständig
**Halogene (NaOCl/Chloramin T, Chlordioxid)**	rascher Zerfall, bei Chloramin bleibt das Sulfonamidmole-kül zurück	geruchsbelästigend, selten Sensibilisierung	bei kleinflächiger Anwendung von Chlordioxid Unterschreitung des 15 min Werts für berufsbedingte Exposition mit 0,3 ppm und für 8 h mit 0,1 ppm (Geruchsschwelle 0,1 ppm)	unvollständig, Bildung von AOX
**Quartäre Ammoniumver-bindungen (QAV)**	ausgeprägt mit langer Haftung	dermale Resorption, Kontaktallergen, mit Asthma assoziiert, inhalative Resorption	Gesundheitsgefährdung bei großflächiger Langzeitanwendung	mäßig
**Alklylamine**	Haftung mit unbekannter Dauer	kein Anhalt für Risiken		gut
**Glucoprotamin**	Haftung mit unbekannter Dauer	sehr schwaches Kontaktallergen	unbedenklich	rasch und vollständig
**Phenolderivate**	Adhäsion an Kunststoffe, Gummi und poröse Materialien	unangenehmer Geruch, dermale Resorption, vereinzelt Sensibilisierungen	unbedenklich	strukturabhängig mäßig bis gering

[1] gemäß Verordnung (EG) Nr. 1272/2008 über die Einstufung, Kennzeichnung und Verpackung (CLP) von Stoffen und Gemischen [226]

[2] bezieht sich auf die in Flächendesinfektionsmitteln im Gesundheitswesen verwendeten Wirkstoffen und Konzentrationen

4 Methoden zur Bewertung der Ergebnisqualität der Reinigung bzw. desinfizierenden Flächenreinigung

Da die Methoden verschiedene Endpunkte nutzen, sind sie nicht unmittelbar miteinander vergleichbar. Hinzu kommt, dass es unbekannt ist, wie „sauber" bzw. erregerarm eine Fläche sein muss, dass von ihr kein Risiko zur Übertragung nosokomialer Erreger ausgeht. Sofern das Monitoring jedoch gezielt zur Qualitätsverbesserung einer Maßnahme eingesetzt wird, ist die Auswahl der Methode nachgeordnet, so dass die Entscheidung von den Möglichkeiten der Einrichtung und dem Aufwand abhängig gemacht werden sollte.

4.1 Audit

Während die alleinige Sichtkontrolle nicht zielführend ist, ermöglichen Audits mit detaillierten Checklisten eine realistische Bewertung [227].

4.2 Mikrobiologische Beprobung

Die mikrobiologische Untersuchung von Oberflächen mittels Kontaktkultur oder Abstrich wird seit Jahrzehnten angewandt. Bei der Kontaktkultur (sog. „Abklatsch"-Methode) werden vorgefertigte Agar-Platten auf die zu untersuchende Oberfläche aufgepresst. Sofern es sich um wischdesinfizierte Oberflächen handelt, ist abhängig vom eingesetzten Wirkstoff ein Agar mit Neutralisierungzusatz zu verwenden. Nach Bebrütung über 24-48 h werden die Anzahl koloniebildender Einheiten (KbE) gezählt und nach Einschätzung des Krankenhaushygienikers ggf. eine Differenzierung vorgenommen. Mit der Abstrichmethode wird eine Oberfläche mit einem sterilen, angefeuchteten Watteträger oder einem sterilen Wischtuch abgewischt (Neutralisierung beachten). In der Regel wird nicht auf anaerobe Bakterien untersucht. Mitunter wird zur Beurteilung des Hygienestatus das Vorkommen sog. Indikatororganismen analysiert, z.B. von Enterobacteriaceae im Küchenbereich. Grundsätzlich sind anhand von KbE keine Aussagen zu einem Infektionsrisiko ableitbar [228]. Auch wenn es Vorschläge für Benchmarks zum Nachweis aerober Bakterien auf gereinigten und desinfizierten Oberflächen gibt, die von < 2,5 KbE/cm^2 bis 5 KbE/cm^2 reichen [229, 230], muss vor Ort eine krankenhaushygienische Interpretation erfolgen. Typische nosokomiale Infektionserreger sollen auf sachgerecht gereinigten und desinfizierten Flächen nicht nachweisbar sein (< 1 KbE/cm^2). In Ausbruchssituationen wird die Untersuchung auf das Vorkommen des jeweiligen Erregers empfohlen [230, 231]. Da auch zwischen der Koloniezahl und dem Vorkommen von Indikatororganismen keine oder nur geringe Korrelationen gefunden werden, wurde vorgeschlagen, beide Verfahren zu kombinieren [229, 230].

Die mikrobiologische Methode ist zeit- und kostenaufwändig, bedarf der standardisierten Probenahme und Laborkapazität, der größte Nachteil besteht aber im Zeitaufwand von 1-2 d, bis ein Ergebnis vorliegt. Deswegen werden mikrobiologische Untersuchungen nur zur Analyse von Ausbruchssituationen empfohlen [230-232].

4.3 ATP-Methode

Der Nachweis von Adenosintriphosphat (ATP) zeigt die Anwesenheit von organischem Material einschließlich mikrobieller Kontamination an. Die Biolumineszenztests verschiedener Hersteller basieren auf einer chemischen Reaktion, die mit Luciferase katalysiert wird (D-Luciferin + 0_2 + ATP $\xrightarrow{\text{Luciferase}}$ Oxyluciferin + CO_2 + Adenosinmonophosphat (AMP) + Diphosphat (PP) + Licht). Die Licht-Menge (Biolumineszenz), die durch diese Reaktion generiert wird und proportional zur Menge von ATP ist, wird in relativen Lichteinheiten (RLU) angegeben. Die Messung der Lichtintensität mit einem Bioluminimeter ermöglicht ein schnelles, unmittelbares Monitoring der Anwesenheit von organischem Material. Als Benchmark im klinischen Setting wurden in verschiedenen Studien bei unterschiedlich großen beprobten Flächen und Probematerialien unterschiedlicher Firmen zur Definition von „reinen" oder „akzeptablen" Oberflächen unterschiedliche RLU mit 100, 250 bzw. 500 RLU angegeben [233, 234]. Bei einer Adjustierung der ATP-Ergebnisse auf die beprobte Fläche (in der Regel 100 cm^2) wurden „Grenzwerte" von 5 RLU/cm^2 [235] und 10 RLU/cm^2 [236] vorgeschlagen. In einer neueren Untersuchung wurden „Grenzwerte" von 40 RLU/cm^2 für Oberflächen mit mittlerem Risiko und 50 RLU/cm^2 für Oberflächen mit niedrigem Risiko angegeben [237]. Die ATP Messung erfasst nicht nur mikrobielles ATP, sondern ATP in organischem Material. ATP-Tests verschiedener Hersteller wiesen in der Labor-Testsituation über einen weiten Bereich lineare Zusammenhänge zwischen dem Nachweis aerober Bakterien und RUL auf, waren in der Empfindlichkeit der Detektion organischen Materials unterschiedlich und wiesen eine unterschiedlich starke negative Beeinflussung durch verschiedene Reinigungs- und Desinfektionsmittel auf [238, 239]. Darüber hinaus konnte gezeigt werden, dass auch die Beschaffenheit der Oberflächen selbst die Ergebnisse der ATP-Messung stark beeinflussen kann. Auf raueren Oberflächen wurde die mikrobiologische Erregerlast mit der Biolumineszenzmethode deutlich unterschätzt [240].

Die ATP-Methode wurde ursprünglich zur Hygienekontrolle in der Lebensmittelindustrie entwickelt und hat sich dort als geeignet erwiesen, nicht nur mikrobielle Verunreinigungen, sondern auch Reste von Lebensmitteln und anderem organischen Material anzuzeigen. Bereits 2013 warnten Shama und Malik [241] vor dem unkritischen Einsatz der ATP-Methode im medizinischen Setting und betonten, dass der ATP-Nachweis nicht als Surrogat-Indikator für die Anwesenheit von Mikroorganismen gewertet werden dürfe. Inzwischen liegen Reviews zur Bewertung der Biolumineszenzmethode im

klinischen Setting vor. Bereits 2014 hatten Amodio und Dino die ATP-Methode anhand von 12 Studien aus UK, den Vereinigten Staaten von Amerika (USA) und Brasilien, die 2000-2011 publiziert worden waren, evaluiert [233]. Die Autoren beschrieben die ATP-Methode als schnelle, objektive Monitoring-Methode, mahnten allerdings die Standardisierung auf nationalem und internationalem Level an. Als Limitation wurden darüber hinaus die relativ hohen Kosten für die Tests angegeben. Das Review von Nante et al. [234] umfasst 27 zwischen 2000 und 2017 erschienene Arbeiten unter Einschluss der von Amodio und Dino [233] berücksichtigten Arbeiten. Es wird über große Unterschiede in den Benchmarks zur Unterscheidung rein vs. unrein berichtet (45-1000 RLU), wobei Benchmarks von 250 und 500 RLU am häufigsten eingesetzt wurden und manche Autoren unterschiedliche Benchmarks für unterschiedliche Testsysteme oder auch unterschiedliche Benchmarks für unterschiedliche Flächen nutzten, z.B. 100 RLU für patientennahe Flächen und 300 RLU für Fußböden [236]. In 14 Studien wurden die RLU-Werte mit mikrobiologischen Untersuchungen (in der Regel Anaerobier und Benchmark 2,5 KbE/cm^2) verglichen. In 3 Studien wurde keine, in 11 Studien schwache Korrelationen zwischen KbE und RLU gefunden. Die Autoren schlussfolgern, dass die ATP-Biolumineszenzmethode keine sichere Methode zur Detektion von Bakterien darstellt, weil sie nicht nur Bakterien, sondern auch anderes organisches Material wie Urin, Blut, Milch etc. miterfasst, und durch Reinigungs- und Desinfektionsmittel negativ beeinflusst wird. Insgesamt wird die Schlussfolgerung abgeleitet, dass die ATP-Methode eine nützliche Methode zur Untersuchung der Krankenhaushygiene sei, wenn die Grenzen der Methode beachtet werden.

4.4 Fluoreszenzverfahren

Das Fluoreszenzverfahren wurde 2006 von Carling et al. nach Einsatz in 3 Kliniken mit einer selbst hergestellten fluoreszierenden Lösung eingeführt [242]. Inzwischen werden fluoreszierende Lösungen von verschiedenen Herstellern zur Kontrolle der Reinigungsleistung angeboten. Bei dem Fluoreszenzverfahren wird eine Fläche mit dem fluoreszierenden Farbstoff markiert. Dieser kann mit einem Stift, Stempel oder Spray aufgetragen werden. Mit einer Schwarzlichtlampe wird dann – nach einmaliger Reinigung oder nach mehreren d – getestet, ob die Markierung durch die Reinigung entfernt wurde. Die Bewertung erfolgt semiquantitativ: eine nicht entfernte Markierung wird mit 0 Punkten, eine teilweise Entfernung mit einem und eine vollständige Entfernung mit 2 Punkten bewertet. Die Methode ist einfach, vergleichsweise preiswert und eignet sich für die unmittelbare Darstellung der Reinigungsleistung, weshalb sie auch für Schulungszwecke und direktes Feedback genutzt wird. Es ist zu beachten, dass die Methode nicht auf alten, angegriffenen Oberflächen funktioniert und nur die Qualität der Reinigung (Wischen mit Druck) anzeigt, aber keinen Hinweis auf einen Desinfektionserfolg gibt [243]. Vor diesem Hintergrund ist es nicht überraschend, dass beim Vergleich der Fluoreszenzmethode mit anderen quantitativen Methoden (aerobe Koloniezahl sowie

Nachweis und ATP-Biolumineszenz) nur ein geringer Zusammenhang zwischen Fluoreszenzmethode und aerober Koloniezahl sowie ATP-Nachweis – abhängig von der behandelten Oberfläche – gefunden wurde und > 20 % der mit Fluoreszenz als „rein“ definierten Flächen > 2,5 KbE Aerobier/cm^2 aufwiesen bzw. über 40 % ATP > 250 RLU ergaben [244]. In einer anderen Untersuchung wurde die UV-Methode zur Kontrolle der Reinigungsleistung eingesetzt und die Ergebnisse wurden mit dem Nachweis von *C. difficile*-Sporen verglichen. Selbst bei optimalen Fluoreszenz-Werten waren in 41 % der Flächen noch *C. difficile*-Sporen nachweisbar [245]. Snyder et al. [246] haben, bezogen auf die mikrobiologische Untersuchung (Benchmark < 5 KbE/cm^2), ebenfalls nur eine geringe Sensitivität (51 %) und Spezifität (56 %) der Fluoreszenzmethode festgestellt.

Im Rahmen verschiedener Interventionsstudien [242, 243, 245, 247-255] erwies sich die Fluoreszenzmethode zum Monitoring als geeignet. Durch den Einsatz der Monitoring-Methode insbesondere im Rahmen von Schulungen konnten deutliche Verbesserungen in der Reinigungsleistung erzielt werden. Nach Hausemann et al. [243] führten in Frankfurt am Main alle Kliniken regelmäßig optische Kontrollen durch und über 80 % der Kliniken setzten im Jahr 2016 zusätzlich die Fluoreszenz-Methode für das Monitoring der Reinigungsleistung ein. Auch in stationären Pflegeeinrichtungen wurde die Fluoreszenzmethode mit Erfolg eingesetzt [256].

4.5 Optische Kontrolle

Mit der optischen Kontrolle werden Flächen auf Schmutzreste, Staub, Putzschlieren etc. überprüft [257]. Als „sauber“ eingestufte Flächen können dennoch mikrobiell verunreinigt sein, was mikrobiologisch und mittels der Biolumineszenzmethode für ATP nachgewiesen wurde, d.h. die tatsächliche mikrobiologische Last einer Fläche wird durch alleinige optische Kontrolle unterschätzt [235, 258-260]. Die geringe Sensitivität der optischen Kontrolle wurde auch in einer Studie im Vergleich mit einem ATP-Biolumineszenztest und dem Nachweis aerober KbE bestätigt [261]. In einer prospektiven Studie zur Überprüfung der Schlussdesinfektion auf patientennahen Handkontaktflächen wurden visuelle Kontrolle, Fluoreszenzmethode und ATP-Biolumineszenz mit der mikrobiologischen Untersuchung verglichen [246]. 72 % der getesteten Oberflächen wurden bei der mikrobiologischen Untersuchung als „rein“ (< 5 KbE/cm^2) eingestuft. Die Sensitivität der visuellen Kontrolle, der Fluoreszenzmethode bzw. der ATP-Biolumineszenz für eine saubere Oberfläche betrug 60 %, 51 % bzw. 70 %, die Spezifität lag bei 52 %, 56 % bzw. 44 %. Unter Hinweis auf die nur schwachen Assoziationen der verschiedenen Methoden im Vergleich zur mikrobiologischen Testung empfehlen die Autoren die visuelle Kontrolle als mindestens ebenso gut geeignet für die Überprüfung einer Schlussdesinfektion wie die kommerziellen Methoden des Fluoreszenz- und des ATP-Tests. Dabei wurde positiv bewertet, dass die optische Kontrolle auch der Wahrnehmung des Patienten entspricht

sowie einfach und preiswert durchführbar ist. Knape et al. [262] verglichen die visuelle Kontrolle mit der ATP-Biolumineszenz-Methode. Angesichts der Schwierigkeiten der Interpretation der variablen und punktuellen ATP-Ergebnisse schätzen sie aus der Perspektive der Qualitätskontrolle die optische Kontrolle als besonders gut geeignet ein, da hier rasch ein Überblick über alle Oberflächen erreicht werden kann und die Unsicherheiten in der Bewertung geringer sind. Angesichts der Subjektivität der Methode vermuten die Autoren jedoch, dass die objektive ATP-Methode von den Reinigungskräften besser akzeptiert wird. Hayden et al. [2] konnten nachweisen, dass mittels visueller Kontrolle der desinfizierenden Flächenreinigung mit einem QAV durch einen trainierten Beobachter die Flächenbelastung mit VRE signifikant und die VRE-Kolonisation tendenziell reduziert wurde.

5 Prüfmethoden zur Bestimmung der Wirksamkeit von Flächendesinfektionsverfahren

Bei den Prüfmethoden werden Suspensionsversuche und praxisnahe Tests unterschieden. Suspensionsversuche dienen vorrangig der Orientierung für die Prüfbedingungen in den nachfolgenden praxisnahen Tests. Hierbei werden die Testorganismen mit einem großen Überschuss an Desinfektionsmittel gemischt (1:10). Daraus lassen sich nur sehr bedingt Rückschlüsse auf die Wirkung eines Desinfektionsmittels auf einer Oberfläche ziehen. Die praxisnahen Prüfungen berücksichtigen weitgehend die Anwendungsbedingungen durch Wischen (d.h. mit mechanischer Einwirkung) oder Sprühen (d.h. ohne mechanische Einwirkung). Zur Prüfung mit Mechanik wird der 4-Felder-Test angewendet, bei dem ein mit einer definierten Menge an Testorganismen + Testanschmutzung kontaminiertes Testfeld auf einem PVC-Streifen mit einem Desinfektionsmittel getränkten Tuch unter genau festgelegten Bedingungen gewischt wird. Anschließend wird untersucht, wie viele der Testorganismen nach der vorgesehenen Einwirkzeit noch nachweisbar sind. Dabei wird gleichzeitig die Verschleppung von Testorganismen auf weitere auf dem PVC-Streifen befindliche Felder untersucht, die zuvor nicht kontaminiert waren und die in denselben Wischvorgang einbezogen sind. Für die Prüfung ohne Mechanik wird eine im Verhältnis deutlich größere Menge des Desinfektionsmittels auf ein kontaminiertes Edelstahlplättchen aufgebracht. Nach der Einwirkzeit wird ebenfalls die Menge der nicht abgetöteten Testorganismen ermittelt.

Tabelle 7: Prüforganismen zur Deklarierung der unterschiedlichen Wirkbereiche gemäß VAH [263-266]

Wirkspektrum	Testorganismen
Bakterizid[a]	*S. aureus, Enterococcus hirae, P. aeruginosa; Proteus mirabilis*[b], *E. coli*[b], falls diese im qualitativen Suspensionstest resistenter als *P. aeruginosa* sind
Tuberkulozid	*Mycobacterium (M.) terrae*
Mykobakterizid	*M. terrae* + *M. avium*
Levurozid[a]	*C. albicans*
Fungizid	*A. brasiliensis* (quantifizierbare Testung nur mit Sporen möglich)
Sporizid	*C. difficile*-Sporen
Begrenzt viruzid	Vacciniavirus (Stamm Elstree bzw. MVA), zusätzlich Bovines Virusdiarrhoe-Virus (BVDV)[b] bei oxidativ wirksamen Produkten
Begrenzt viruzid PLUS	Adenovirus Typ 5, murines Norovirus (MNV)
Viruzid	Adenovirus Typ 5, MNV, Poliovirus Typ 1, Simianvirus 40 (SV40)[b], murines Parvovirus (Minute Virus of Mice; MVM)

[a] Basisanforderung für Flächendesinfektionsmittel ist die bakterizide und levurozide Wirkung

[b] nicht in DIN EN Normen enthalten

Der Hersteller gibt das Wirkspektrum und die Anwendungsbedingungen (Konzentration, Zeit, mit oder ohne Mechanik) in der Produktinformation an.

Interessenkonflikt. Dieser informative Anhang wurde ehrenamtlich und ohne Einflussnahme kommerzieller Interessengruppen im Auftrag der Kommission für Krankenhaushygiene und Infektionsprävention unter der Leitung von Prof. Dr. Axel Kramer in einer interdisziplinären Arbeitsgruppe, bestehend aus PD Dr. Maren Eggers (Stuttgart), Prof. Hans-Curt Flemming (Duisburg-Essen), Prof. Ursel Heudorf (Frankfurt am Main), MD MSc. Martin Misailovski (Göttingen), Prof. Simone Scheithauer (Göttingen) und Prof. Ulrike Seifert (Greifswald) erstellt. Vom Robert Koch-Institut waren Dr. Franziska Lexow und Marc Thanheiser beteiligt. Der informative Anhang wurde durch die Arbeitsgruppe vorbereitet und nach ausführlicher Diskussion in der Kommission abgestimmt.

Literatur

1. Allen KD, Green HT (1987) Hospital outbreak of multi-resistant Acinetobacter anitratus: an airborne mode of spread? J Hosp Infect 9(2):110-119. https://doi.org/10.1016/0195-6701(87)90048-x
2. Hayden MK, Bonten MJM, Blom DW, Lyle EA, van de Vijver DAMC, Weinstein RA (2006) Reduction in acquisition of vancomycin-resistant enterococcus after enforcement of routine environmental cleaning measures. Clin Infect Dis 42(11):1552-1560. https://doi.org/Doi 10.1086/503845
3. Mitchell BG, Dancer SJ, Anderson M, Dehn E (2015) Risk of organism acquisition from prior room occupants: a systematic review and meta-analysis. J Hosp Infect 91(3):211-217. https://doi.org/10.1016/j.jhin.2015.08.005
4. Wu HM, Fornek M, Schwab KJ et al (2005) A norovirus outbreak at a long-term-care facility: the role of environmental surface contamination. Infect Control Hosp Epidemiol 26(10):802-810. https://doi.org/10.1086/502497
5. Suleyman G, Alangaden G, Bardossy AC (2018) The Role of Environmental Contamination in the Transmission of Nosocomial Pathogens and Healthcare-Associated Infections. Curr Infect Dis Rep 20(6):12. https://doi.org/10.1007/s11908-018-0620-2
6. Oie S, Kamiya A (1996) Survival of methicillin-resistant Staphylococcus aureus (MRSA) on naturally contaminated dry mops. J Hosp Infect 34(2):145-149. https://doi.org/10.1016/s0195-6701(96)90140-1
7. Donskey CJ (2004) The role of the intestinal tract as a reservoir and source for transmission of nosocomial pathogens. Clin Infect Dis 39(2):219-226. https://doi.org/10.1086/422002
8. Otter JA, Yezli S, French GL (2011) The role played by contaminated surfaces in the transmission of nosocomial pathogens. Infect Control Hosp Epidemiol 32(7):687-699. https://doi.org/10.1086/660363
9. Stewart PS (2015) Antimicrobial Tolerance in Biofilms. Microbiol Spectr 3(3). https://doi.org/10.1128/microbiolspec.MB-0010-2014
10. Wingender J, Flemming HC (2011) Biofilms in drinking water and their role as reservoir for pathogens. Int J Hyg Environ Health 214(6):417-423. https://doi.org/10.1016/j.ijheh.2011.05.009
11. Yan J, Bassler BL (2019) Surviving as a Community: Antibiotic Tolerance and Persistence in Bacterial Biofilms. Cell Host Microbe 26(1):15-21. https://doi.org/10.1016/j.chom.2019.06.002
12. Li L, Mendis N, Trigui H, Oliver JD, Faucher SP (2014) The importance of the viable but non-culturable state in human bacterial pathogens. Front Microbiol 5:258. https://doi.org/10.3389/fmicb.2014.00258
13. Wideman NE, Oliver JD, Crandall PG, Jarvis NA (2021) Detection and Potential Virulence of Viable but Non-Culturable (VBNC) Listeria monocytogenes: A Review. Microorganisms 9(1):194. https://doi.org/10.3390/microorganisms9010194
14. Hübner NO, Hübner C, Kramer A, Assadian O (2011) Survival of bacterial pathogens on paper and bacterial retrieval from paper to hands: preliminary results. Am J Nurs 111(12):30-34. https://doi.org/10.1097/01.NAJ.0000408181.37017.82
15. Rangel-Frausto MS, Houston AK, Bale MJ, Fu C, Wenzel RP (1994) An experimental model for study of Candida survival and transmission in human volunteers. Eur J Clin Microbiol Infect Dis 13(7):590-595. https://doi.org/10.1007/BF01971311
16. von Rheinbaben F, Schunemann S, Gross T, Wolff MH (2000) Transmission of viruses via contact in a household setting: experiments using bacteriophage phi X174 as a model virus. J Hosp Infect 46(1):61-66. https://doi.org/10.1053/jhin.2000.0794
17. Kramer A, Assadian O (2014) Survival of Microorganisms on Inanimate Surfaces. In: Borkow G (Hrsg) Use of Biocidal Surfaces for Reduction of Healthcare Acquired Infections Springer, Cham, S 7–26

18. Schubert H (1989) Über bakterielle Absterbekurven. In: Weichardt W (Hrsg) Ergebnisse der Hygiene Bakteriologie Immunitätsforschung und experimentellen Therapie, Bd. 22. Springer, Berlin, S 69-137
19. Neely AN (2000) A survey of gram-negative bacteria survival on hospital fabrics and plastics. J Burn Care Rehabil 21(6):523-527. https://doi.org/10.1097/00004630-200021060-00009
20. Blaschke-Hellmessen R, Kreuz M, Sprung M (1985) Umweltresistenz und natürliche Keimreservoire medizinisch bedeutsamer Sproßpilze. Z gesamte Hyg 31(12):712-715
21. Grass G, Rensing C, Solioz M (2011) Metallic copper as an antimicrobial surface. Appl Environ Microbiol 77(5):1541-1547. https://doi.org/10.1128/aem.02766-10
22. Ojeil M, Jermann C, Holah J, Denyer SP, Maillard JY (2013) Evaluation of new in vitro efficacy test for antimicrobial surface activity reflecting UK hospital conditions. J Hosp Infect 85(4):274-281. https://doi.org/10.1016/j.jhin.2013.08.007
23. Tang JW (2009) The effect of environmental parameters on the survival of airborne infectious agents. J R Soc Interface 6 (Suppl 6):S737-S746. https://doi.org/10.1098/rsif.2009.0227.focus
24. McDade JJ, Hall LB (1964) Survival of Staphylococcus Aureus in the Environment. II. Effect of Elevated Temperature on Surface-Exposed Staphylococci. Am J Hyg 80:184-191. https://doi.org/10.1093/oxfordjournals.aje.a120467
25. Robine E, Derangere D, Robin D (2000) Survival of a Pseudomonas fluorescens and Enterococcus faecalis aerosol on inert surfaces. Int J Food Microbiol 55(1-3):229-234. https://doi.org/10.1016/s0168-1605(00)00188-4
26. Jawad A, Heritage J, Snelling AM, Gascoyne-Binzi DM, Hawkey PM (1996) Influence of relative humidity and suspending menstrua on survival of Acinetobacter spp. on dry surfaces. J Clin Microbiol 34(12):2881-2887. https://doi.org/10.1128/jcm.34.12.2881-2887.1996
27. Gsell O (1968) Meningokokkeninfektionen. In: Gsell O, Mohr O (Hrsg) Krankheiten durch Bakterien, Bd. II/1. Springer, Berlin, Heidelberg, S 133-173
28. Wood JP, Meyer KM, Kelly TJ et al (2015) Environmental Persistence of Bacillus anthracis and Bacillus subtilis Spores. PLoS One 10(9):e0138083. https://doi.org/10.1371/journal.pone.0138083
29. Zeidler S, Müller V (2019) The role of compatible solutes in desiccation resistance of Acinetobacter baumannii. MicrobiologyOpen 8(5):e00740. https://doi.org/10.1002/mbo3.740
30. Kim KH, Fekety R, Batts DH et al (1981) Isolation of Clostridium difficile from the environment and contacts of patients with antibiotic-associated colitis. J Infect Dis 143(1):42-50. https://doi.org/10.1093/infdis/143.1.42
31. Otter JA, French GL (2009) Survival of nosocomial bacteria and spores on surfaces and inactivation by hydrogen peroxide vapor. J Clin Microbiol 47(1):205-207. https://doi.org/10.1128/JCM.02004-08
32. Jump RL, Pultz MJ, Donskey CJ (2007) Vegetative Clostridium difficile survives in room air on moist surfaces and in gastric contents with reduced acidity: a potential mechanism to explain the association between proton pump inhibitors and C. difficile-associated diarrhea? Antimicrob Agents Chemother 51(8):2883-2887. https://doi.org/10.1128/AAC.01443-06
33. Crosbie WE, Wright HD (1941) Diphtheria bacilli in floor dust. Lancet 237(6143):656-659. https://doi.org/https://doi.org/10.1016/S0140-6736(00)61019-X
34. Augustine JL, Renshaw HW (1986) Survival of Corynebacterium pseudotuberculosis in axenic purulent exudate on common barnyard fomites. Am J Vet Res 47(4):713-715
35. Koca O, Altoparlak U, Ayyildiz A, Kaynar H (2012) Persistence of nosocomial pathogens on various fabrics. Eurasian J Med 44(1):28-31. https://doi.org/10.5152/eajm.2012.06
36. Katzenberger RH, Rosel A, Vonberg RP (2021) Bacterial survival on inanimate surfaces: a field study. BMC Res Notes 14(1):97. https://doi.org/10.1186/s13104-021-05492-0
37. Wendt C, Wiesenthal B, Dietz E, Rüden H (1998) Survival of vancomycin-resistant and vancomycin-susceptible enterococci on dry surfaces. J Clin Microbiol 36(12):3734-3736. https://doi.org/10.1128/jcm.36.12.3734-3736.1998

38. Wendt C, Dietze B, Dietz E, Ruden H (1997) Survival of Acinetobacter baumannii on dry surfaces. J Clin Microbiol 35(6):1394-1397. https://doi.org/10.1128/jcm.35.6.1394-1397.1997
39. Neely AN, Maley MP (2000) Survival of enterococci and staphylococci on hospital fabrics and plastic. J Clin Microbiol 38(2):724-726. https://doi.org/10.1128/JCM.38.2.724-726.2000
40. Esteves DC, Pereira VC, Souza JM et al (2016) Influence of biological fluids in bacterial viability on different hospital surfaces and fomites. Am J Infect Control 44(3):311-314. https://doi.org/10.1016/j.ajic.2015.09.033
41. Hirai Y (1991) Survival of bacteria under dry conditions; from a viewpoint of nosocomial infection. J Hosp Infect 19(3):191-200. https://doi.org/10.1016/0195-6701(91)90223-u
42. Bale MJ, Bennett PM, Hinton M, Beringer JE (1990) The survival of genetically engineered microorganisms and bacteria on inanimate surfaces and in animals. In: Fry JC, Day MJ (Hrsg) Bacterial Genetics in Natural Environments. Springer, Dordrecht, S 231-239 https://doi.org/10.1007/978-94-009-1834-4_18
43. Smith CR (1942) Survival of Tubercle Bacilli. American Review of Tuberculosis 45(3):334-345. https://doi.org/10.1164/art.1942.45.3.334
44. Marshall B, Levy S (2011) Microbial contamination of musical wind instruments. Int J Environ Health Res 21(4):275-285. https://doi.org/10.1080/09603123.2010.550033
45. Zarpellon MN, Gales AC, Sasaki AL et al (2015) Survival of vancomycin-intermediate Staphylococcus aureus on hospital surfaces. J Hosp Infect 90(4):347-350. https://doi.org/10.1016/j.jhin.2015.04.005
46. Webster C, Towner KJ, Humphreys H (2000) Survival of Acinetobacter on three clinically related inanimate surfaces. Infect Control Hosp Epidemiol 21(4):246. https://doi.org/10.1086/503214
47. Dickgiesser N (1978) Untersuchungen über das Verhalten grampositiver und gramnegativer Bakterien in trockenem und feuchtem Milieu. Zentralbl Bakteriol Mikrobiol Hyg B 167:48-62
48. Gundermann K (1972) Untersuchungen zur Lebensdauer von Bakterienstämmen im Staub unter dem Einfluß unterschiedlicher Luftfeuchtigkeit. Zentralbl Bakteriol Mikrobiol Hyg B 156:422-429
49. Kampf G, Dietze B, Grosse-Siestrup C, Wendt C, Martiny H (1998) Microbicidal activity of a new silver-containing polymer, SPI-ARGENT II. Antimicrob Agents Chemother 42(9):2440-2442. https://doi.org/10.1128/AAC.42.9.2440
50. Wagenvoort JH, Penders RJ (1997) Long-term in-vitro survival of an epidemic MRSA phage-group III-29 strain. J Hosp Infect 35(4):322-325. https://doi.org/10.1016/s0195-6701(97)90229-2
51. Noyce JO, Michels H, Keevil CW (2006) Potential use of copper surfaces to reduce survival of epidemic meticillin-resistant Staphylococcus aureus in the healthcare environment. J Hosp Infect 63(3):289-297. https://doi.org/10.1016/j.jhin.2005.12.008
52. Wagenvoort JH, Sluijsmans W, Penders RJ (2000) Better environmental survival of outbreak vs. sporadic MRSA isolates. J Hosp Infect 45(3):231-234. https://doi.org/10.1053/jhin.2000.0757
53. Giannouli M, Antunes LC, Marchetti V, Triassi M, Visca P, Zarrilli R (2013) Virulence-related traits of epidemic Acinetobacter baumannii strains belonging to the international clonal lineages I-III and to the emerging genotypes ST25 and ST78. BMC Infect Dis 13:282. https://doi.org/10.1186/1471-2334-13-282
54. Weese JS, Jarlot C, Morley PS (2009) Survival of Streptococcus equi on surfaces in an outdoor environment. Can Vet J 50(9):968-970
55. Durham AE, Hall YS, Kulp L, Underwood C (2018) A study of the environmental survival of Streptococcus equi subspecies equi. Equine Vet J 50(6):861-864. https://doi.org/10.1111/evj.12840
56. Ingham SC, Wadhera RK, Chu CH, DeVita MD (2006) Survival of Streptococcus pyogenes on foods and food contact surfaces. J Food Prot 69(5):1159-1163. https://doi.org/10.4315/0362-028x-69.5.1159

57. Marks LR, Reddinger RM, Hakansson AP (2014) Biofilm formation enhances fomite survival of Streptococcus pneumoniae and Streptococcus pyogenes. Infect Immun 82(3):1141-1146. https://doi.org/10.1128/IAI.01310-13
58. Tagg JR, Ragland NL (1991) Applications of BLIS typing to studies of the survival on surfaces of salivary streptococci and staphylococci. J Appl Bacteriol 71(4):339-342. https://doi.org/10.1111/j.1365-2672.1991.tb03797.x
59. Espinal P, Marti S, Vila J (2012) Effect of biofilm formation on the survival of Acinetobacter baumannii on dry surfaces. J Hosp Infect 80(1):56-60. https://doi.org/10.1016/j.jhin.2011.08.013
60. Jawad A, Snelling AM, Heritage J, Hawkey PM (1998) Exceptional desiccation tolerance of Acinetobacter radioresistens. J Hosp Infect 39(3):235-240. https://doi.org/10.1016/s0195-6701(98)90263-8
61. Jawad A, Seifert H, Snelling AM, Heritage J, Hawkey PM (1998) Survival of Acinetobacter baumannii on dry surfaces: comparison of outbreak and sporadic isolates. J Clin Microbiol 36(7):1938-1941. https://doi.org/10.1128/jcm.36.7.1938-1941.1998
62. Antunes LC, Imperi F, Carattoli A, Visca P (2011) Deciphering the multifactorial nature of Acinetobacter baumannii pathogenicity. PLoS One 6(8):e22674. https://doi.org/10.1371/journal.pone.0022674
63. Farrow JM, 3rd, Wells G, Pesci EC (2018) Desiccation tolerance in Acinetobacter baumannii is mediated by the two-component response regulator BfmR. PLoS One 13(10):e0205638. https://doi.org/10.1371/journal.pone.0205638
64. Musa EK, Desai N, Casewell MW (1990) The survival of Acinetobacter calcoaceticus inoculated on fingertips and on formica. J Hosp Infect 15(3):219-227. https://doi.org/10.1016/0195-6701(90)90029-n
65. Oosterom J, de Wilde GJA, de Boer E, de Blaauw LH, Karman H (1983) Survival of Campylobacter jejuni during Poultry Processing and Pig Slaughtering. J Food Prot 46(8):702-706. https://doi.org/10.4315/0362-028X-46.8.702
66. Boucher SN, Chamberlain AH, Adams MR (1998) Enhanced Survival of Campylobacter jejuni in Association with Wood. J Food Prot 61(1):26-30. https://doi.org/10.4315/0362-028x-61.1.26
67. Siroli L, Patrignani F, Serrazanetti DI et al (2017) Survival of Spoilage and Pathogenic Microorganisms on Cardboard and Plastic Packaging Materials. Front Microbiol 8:2606. https://doi.org/10.3389/fmicb.2017.02606
68. Hokunan H, Koyama K, Hasegawa M, Kawamura S, Koseki S (2016) Survival Kinetics of Salmonella enterica and Enterohemorrhagic Escherichia coli on a Plastic Surface at Low Relative Humidity and on Low-Water Activity Foods. J Food Prot 79(10):1680-1692. https://doi.org/10.4315/0362-028x.jfp-16-081
69. Williams AP, Avery LM, Killham K, Jones DL (2005) Persistence of Escherichia coli O157 on farm surfaces under different environmental conditions. J Appl Microbiol 98(5):1075-1083. https://doi.org/10.1111/j.1365-2672.2004.02530.x
70. Ak NO, Cliver DO, Kaspar CW (1994) Decontamination of Plastic and Wooden Cutting Boards for Kitchen Use. J Food Prot 57(1):23-30. https://doi.org/10.4315/0362-028x-57.1.23
71. Abrishami SH, Tall BD, Bruursema TJ, Epstein PS, Shah DB (1994) Bacterial Adherence and Viability on cutting board surfaces. J Food Saf 14(2):153-172. https://doi.org/https://doi.org/10.1111/j.1745-4565.1994.tb00591.x
72. Richter WR, Sunderman MM, Wendling MQS et al (2020) Evaluation of altered environmental conditions as a decontamination approach for nonspore-forming biological agents. J Appl Microbiol 128(4):1050-1059. https://doi.org/10.1111/jam.14532
73. Böhmler G, Gerwert J, Scupin E, Sinell HJ (1996) Zur Epidemiologie der Helicobacteriose des Menschen; Untersuchungen zur Überlebensfähigkeit des Erregers in Lebensmitteln. Dtsch Tierarztl Wochenschr 103(10):438-443

74. Helke DM, Wong ACL (1994) Survival and Growth Characteristics of Listeria monocytogenes and Salmonella typhimurium on Stainless Steel and Buna-N Rubber. J Food Prot 57(11):963-968. https://doi.org/10.4315/0362-028X-57.11.963
75. Hansen LT, Vogel BF (2011) Desiccation of adhering and biofilm Listeria monocytogenes on stainless steel: Survival and transfer to salmon products. Int J Food Microbiol 146(1):88-93. https://doi.org/10.1016/j.ijfoodmicro.2011.01.032
76. Vogel BF, Hansen LT, Mordhorst H, Gram L (2010) The survival of Listeria monocytogenes during long term desiccation is facilitated by sodium chloride and organic material. Int J Food Microbiol 140(2-3):192-200. https://doi.org/10.1016/j.ijfoodmicro.2010.03.035
77. Daneshvar Alavi HE, Truelstrup Hansen L (2013) Kinetics of biofilm formation and desiccation survival of Listeria monocytogenes in single and dual species biofilms with Pseudomonas fluorescens, Serratia proteamaculans or Shewanella baltica on food-grade stainless steel surfaces. Biofouling 29(10):1253-1268. https://doi.org/10.1080/08927014.2013.835805
78. Perez JL, Gomez E, Sauca G (1990) Survival of gonococci from urethral discharge on fomites. Eur J Clin Microbiol Infect Dis 9(1):54-55. https://doi.org/10.1007/BF01969538
79. Elmros T (1977) Survival of Neisseria gonorrhoeae on surfaces. Acta Derm Venereol 57(2):177-180. https://doi.org/102340/0001555557177180
80. Panagea S, Winstanley C, Walshaw MJ, Ledson MJ, Hart CA (2005) Environmental contamination with an epidemic strain of Pseudomonas aeruginosa in a Liverpool cystic fibrosis centre, and study of its survival on dry surfaces. J Hosp Infect 59(2):102-107. https://doi.org/10.1016/j.jhin.2004.09.018
81. Abdelhamid AG, Yousef AE (2019) The microbial lipopeptide paenibacterin disrupts desiccation resistance in Salmonella enterica serovars Tennessee and Eimsbuettel. Appl Environ Microbiol 85(14):e00739-00719. https://doi.org/10.1128/AEM.00739-19
82. Finn S, Händler K, Condell O et al (2013) ProP is required for the survival of desiccated Salmonella enterica serovar typhimurium cells on a stainless steel surface. Appl Environ Microbiol 79(14):4376-4384. https://doi.org/10.1128/aem.00515-13
83. Ramachandran G, Aheto K, Shirtliff ME, Tennant SM (2016) Poor biofilm-forming ability and long-term survival of invasive Salmonella Typhimurium ST313. Pathog Dis 74(5). https://doi.org/10.1093/femspd/ftw049
84. Robertson MH (1972) Survival of S. typhimurium in floor dust--a possible reservoir of infection in institutions. Public Health 87(1):39-45. https://doi.org/10.1016/s0033-3506(72)80034-9
85. Margas E, Meneses N, Conde-Petit B, Dodd CER, Holah J (2014) Survival and death kinetics of Salmonella strains at low relative humidity, attached to stainless steel surfaces. Int J Food Microbiol 187:33-40. https://doi.org/https://doi.org/10.1016/j.ijfoodmicro.2014.06.027
86. Islam MS, Hossain MA, Khan SI et al (2001) Survival of Shigella dysenteriae type 1 on fomites. J Health Popul Nutr 19(3):177-182
87. Nass W (1977) Zur Überlebensdauer von Shigellen auf Plastwerkstoffen. Z ges Hyg 23:395-397
88. Barua D (1970) Survival of cholera vibrios in food, water and fomites. Public Health Pap 40:29-31
89. Johansson P, Ekstrand-Tobin A, Svensson T, Bok G (2012) Laboratory study to determine the critical moisture level for mould growth on building materials. Int Biodeterior Biodegradation 73:23-32. https://doi.org/https://doi.org/10.1016/j.ibiod.2012.05.014
90. Zilberberg MD, Nathanson BH, Harrington R, Spalding JR, Shorr AF (2018) Epidemiology and Outcomes of Hospitalizations With Invasive Aspergillosis in the United States, 2009-2013. Clin Infect Dis 67(5):727-735. https://doi.org/10.1093/cid/ciy181
91. Faure O, Fricker-Hidalgo H, Lebeau B, Mallaret MR, Ambroise-Thomas P, Grillot R (2002) Eight-year surveillance of environmental fungal contamination in hospital operating rooms and haematological units. J Hosp Infect 50(2):155-160. https://doi.org/10.1053/jhin.2001.1148

92. Santos PE, Córdoba S, Carrillo-Muñoz A, Rodero L, Rubeglio E, Soria M (2010) [Epidemiology of fungaemia in a paediatric hospital of high complexity]. Rev Iberoam Micol 27(4):200-202. https://doi.org/10.1016/j.riam.2010.07.002
93. Garcia-Cruz CP, Aguilar MJN, Arroyo-Helguera OE (2012) Fungal and Bacterial Contamination on Indoor Surfaces of a Hospital in Mexico. Jundishapur J Microbiol 5(3):460-464. https://doi.org/10.5812/jjm.2625
94. Mishra B, Mandal A, Kumar N (1992) Mycotic prosthetic-valve endocarditis. J Hosp Infect 20(2):122-125. https://doi.org/10.1016/0195-6701(92)90115-3
95. Hosseinpour L, Zareei M, Boroujeni ZB, Yaghoubi R, Hashemi S (2017) Survival of Dermatophytes in Skin Scales after 10 Years Storage. Infect Epidemiol Microbiol 3(3):96-99. https://doi.org/10.18869/modares.iem.3.3.96
96. Seebacher C, Bouchara JP, Mignon B (2008) Updates on the epidemiology of dermatophyte infections. Mycopathologia 166(5-6):335-352. https://doi.org/10.1007/s11046-008-9100-9
97. Sugimoto R, Katoh T, Nishioka K (1995) Isolation of dermatophytes from house dust on a medium containing gentamicin and flucytosine. Mycoses 38(9-10):405-410. https://doi.org/10.1111/j.1439-0507.1995.tb00072.x
98. Watanabe K, Taniguchi H, Katoh T (2000) Adhesion of dermatophytes to healthy feet and its simple treatment. Mycoses 43(1-2):45-50. https://doi.org/10.1046/j.1439-0507.2000.00546.x
99. Roberts DT (1992) Prevalence of dermatophyte onychomycosis in the United Kingdom: results of an omnibus survey. Br J Dermatol 126(Suppl 39):23-27. https://doi.org/10.1111/j.1365-2133.1992.tb00005.x
100. Yapar N (2014) Epidemiology and risk factors for invasive candidiasis. Ther Clin Risk Manag 10:95-105. https://doi.org/10.2147/TCRM.S40160
101. Pertowski CA, Baron RC, Lasker BA, Werner SB, Jarvis WR (1995) Nosocomial outbreak of Candida albicans sternal wound infections following cardiac surgery traced to a scrub nurse. J Infect Dis 172(3):817-822. https://doi.org/10.1093/infdis/172.3.817
102. Perlroth J, Choi B, Spellberg B (2007) Nosocomial fungal infections: epidemiology, diagnosis, and treatment. Med Mycol 45(4):321-346. https://doi.org/10.1080/13693780701218689
103. Abbasi F, Samaei MR (2019) The effect of temperature on airborne filamentous fungi in the indoor and outdoor space of a hospital. Environ Sci Pollut Res Int 26(17):16868-16876. https://doi.org/10.1007/s11356-017-0939-5
104. Piedrahita CT, Cadnum JL, Jencson AL, Shaikh AA, Ghannoum MA, Donskey CJ (2017) Environmental Surfaces in Healthcare Facilities are a Potential Source for Transmission of Candida auris and Other Candida Species. Infect Control Hosp Epidemiol 38(9):1107-1109. https://doi.org/10.1017/ice.2017.127
105. Ruiz-Gaitán A, Moret AM, Tasias-Pitarch M et al (2018) An outbreak due to Candida auris with prolonged colonisation and candidaemia in a tertiary care European hospital. Mycoses 61(7):498-505. https://doi.org/10.1111/myc.12781
106. Meis JF, Chowdhary A (2018) Candida auris: a global fungal public health threat. Lancet Infect Dis 18(12):1298-1299. https://doi.org/10.1016/s1473-3099(18)30609-1
107. Tsay S, Welsh RM, Adams EH et al (2017) Notes from the Field: Ongoing Transmission of Candida auris in Health Care Facilities - United States, June 2016-May 2017. MMWR Morb Mortal Wkly Rep 66(19):514-515. https://doi.org/10.15585/mmwr.mm6619a7
108. Lyman M, Forsberg K, Reuben J et al (2021) Notes from the Field: Transmission of Pan-Resistant and Echinocandin-Resistant Candida auris in Health Care Facilities - Texas and the District of Columbia, January-April 2021. MMWR Morb Mortal Wkly Rep 70(29):1022-1023. https://doi.org/10.15585/mmwr.mm7029a2
109. Neely AN, Orloff MM (2001) Survival of some medically important fungi on hospital fabrics and plastics. J Clin Microbiol 39(9):3360-3361. https://doi.org/10.1128/JCM.39.9.3360-3361.2001

110. Weaver L, Michels HT, Keevil CW (2010) Potential for preventing spread of fungi in air-conditioning systems constructed using copper instead of aluminium. Lett Appl Microbiol 50(1):18-23. https://doi.org/10.1111/j.1472-765X.2009.02753.x
111. Traore O, Springthorpe VS, Sattar SA (2002) A quantitative study of the survival of two species of Candida on porous and non-porous environmental surfaces and hands. J Appl Microbiol 92(3):549-555. https://doi.org/10.1046/j.1365-2672.2002.01560.x
112. Anderson JH (1979) In vitro survival of human pathogenic fungi in seawater. Sabouraudia 17(1):1-12
113. Welsh RM, Bentz ML, Shams A et al (2017) Survival, Persistence, and Isolation of the Emerging Multidrug-Resistant Pathogenic Yeast Candida auris on a Plastic Health Care Surface. J Clin Microbiol 55(10):2996-3005. https://doi.org/10.1128/Jcm.00921-17
114. Short B, Brown J, Delaney C et al (2019) Candida auris exhibits resilient biofilm characteristics in vitro: implications for environmental persistence. J Hosp Infect 103(1):92-96. https://doi.org/10.1016/j.jhin.2019.06.006
115. Ren SY, Wang WB, Hao YG et al (2020) Stability and infectivity of coronaviruses in inanimate environments. World J Clin Cases 8(8):1391-1399. https://doi.org/10.12998/wjcc.v8.i8.1391
116. Kampf G, Pfaender S, Goldman E, Steinmann E (2021) SARS-CoV-2 Detection Rates from Surface Samples Do Not Implicate Public Surfaces as Relevant Sources for Transmission. Hygiene 1(1):24-40. https://doi.org/10.3390/hygiene1010003
117. Stowell JD, Forlin-Passoni D, Din E et al (2012) Cytomegalovirus survival on common environmental surfaces: opportunities for viral transmission. J Infect Dis 205(2):211-214. https://doi.org/10.1093/infdis/jir722
118. Tanner BD (2009) Reduction in infection risk through treatment of microbially contaminated surfaces with a novel, portable, saturated steam vapor disinfection system. Am J Infect Control 37(1):20-27. https://doi.org/10.1016/j.ajic.2008.03.008
119. Kramer A, Schwebke I, Kampf G (2006) How long do nosocomial pathogens persist on inanimate surfaces? A systematic review. BMC Infect Dis 6:130. https://doi.org/10.1186/1471-2334-6-130
120. Espinosa AC, Mazari-Hiriart M, Espinosa R, Maruri-Avidal L, Méndez E, Arias CF (2008) Infectivity and genome persistence of rotavirus and astrovirus in groundwater and surface water. Water Res 42(10-11):2618-2628. https://doi.org/10.1016/j.watres.2008.01.018
121. John DE, Rose JB (2005) Review of factors affecting microbial survival in groundwater. Environ Sci Technol 39(19):7345-7356. https://doi.org/10.1021/es047995w
122. Abad FX, Pinto RM, Bosch A (1994) Survival of enteric viruses on environmental fomites. Appl Environ Microbiol 60(10):3704-3710. https://doi.org/10.1128/AEM.60.10.3704-3710.1994
123. Mbithi JN, Springthorpe VS, Sattar SA (1991) Effect of relative humidity and air temperature on survival of hepatitis A virus on environmental surfaces. Appl Environ Microbiol 57(5):1394-1399. https://doi.org/10.1128/aem.57.5.1394-1399.1991
124. Casanova LM, Jeon S, Rutala WA, Weber DJ, Sobsey MD (2010) Effects of air temperature and relative humidity on coronavirus survival on surfaces. Appl Environ Microbiol 76(9):2712-2717. https://doi.org/10.1128/aem.02291-09
125. Morris DH, Yinda KC, Gamble A et al (2021) Mechanistic theory predicts the effects of temperature and humidity on inactivation of SARS-CoV-2 and other enveloped viruses. Elife 10:e65902. https://doi.org/10.7554/eLife.65902
126. Mattison K, Karthikeyan K, Abebe M et al (2007) Survival of calicivirus in foods and on surfaces: experiments with feline calicivirus as a surrogate for norovirus. J Food Prot 70(2):500-503. https://doi.org/10.4315/0362-028x-70.2.500
127. Tiwari A, Patnayak DP, Chander Y, Parsad M, Goyal SM (2006) Survival of two avian respiratory viruses on porous and nonporous surfaces. Avian Dis 50(2):284-287. https://doi.org/10.1637/7453-101205r.1

128. Chatterjee P, Kelly S, Qi M, Werner RM (2020) Characteristics and Quality of US Nursing Homes Reporting Cases of Coronavirus Disease 2019 (COVID-19). JAMA Netw Open 3(7):e2016930. https://doi.org/10.1001/jamanetworkopen.2020.16930
129. van Doremalen N, Bushmaker T, Morris DH et al (2020) Aerosol and Surface Stability of SARS-CoV-2 as Compared with SARS-CoV-1. N Engl J Med 382(16):1564-1567. https://doi.org/10.1056/NEJMc2004973
130. Chin AWH, Chu JTS, Perera MRA et al (2020) Stability of SARS-CoV-2 in different environmental conditions. Lancet Microbe 1(1):e10. https://doi.org/10.1016/S2666-5247(20)30003-3
131. Kasloff SB, Leung A, Strong JE, Funk D, Cutts T (2021) Stability of SARS-CoV-2 on critical personal protective equipment. Sci Rep 11(1):984. https://doi.org/10.1038/s41598-020-80098-3
132. Riddell S, Goldie S, Hill A, Eagles D, Drew TW (2020) The effect of temperature on persistence of SARS-CoV-2 on common surfaces. Virol J 17(1):145. https://doi.org/10.1186/s12985-020-01418-7
133. Ratnesar-Shumate S, Williams G, Green B et al (2020) Simulated Sunlight Rapidly Inactivates SARS-CoV-2 on Surfaces. J Infect Dis 222(2):214-222. https://doi.org/10.1093/infdis/jiaa274
134. Schuit M, Ratnesar-Shumate S, Yolitz J et al (2020) Airborne SARS-CoV-2 Is Rapidly Inactivated by Simulated Sunlight. J Infect Dis 222(4):564-571. https://doi.org/10.1093/infdis/jiaa334
135. Heilingloh CS, Aufderhorst UW, Schipper L et al (2020) Susceptibility of SARS-CoV-2 to UV irradiation. Am J Infect Control 48(10):1273-1275. https://doi.org/10.1016/j.ajic.2020.07.031
136. Yezli S, Otter JA (2011) Minimum Infective Dose of the Major Human Respiratory and Enteric Viruses Transmitted Through Food and the Environment. Food Environ Virol 3(1):1-30. https://doi.org/10.1007/s12560-011-9056-7
137. Johnston CP, Qiu H, Ticehurst JR et al (2007) Outbreak management and implications of a nosocomial norovirus outbreak. Clin Infect Dis 45(5):534-540. https://doi.org/10.1086/520666
138. Fraenkel CJ, Böttiger B, Söderlund-Strand A, Inghammar M (2021) Risk of environmental transmission of norovirus infection from prior room occupants. J Hosp Infect 117:74-80. https://doi.org/10.1016/j.jhin.2021.08.026
139. Gelber SE, Ratner AJ (2002) Hospital-acquired viral pathogens in the neonatal intensive care unit. Semin Perinatol 26(5):346-356. https://doi.org/10.1053/sper.2002.36268
140. Rawlinson S, Ciric L, Cloutman-Green E (2020) COVID-19 pandemic - let's not forget surfaces. J Hosp Infect 105(4):790-791. https://doi.org/10.1016/j.jhin.2020.05.022
141. Khan RM, Al-Dorzi HM, Al Johani S et al (2016) Middle East respiratory syndrome coronavirus on inanimate surfaces: A risk for health care transmission. Am J Infect Control 44(11):1387-1389. https://doi.org/10.1016/j.ajic.2016.05.006
142. Biryukov J, Boydston JA, Dunning RA et al (2020) Increasing Temperature and Relative Humidity Accelerates Inactivation of SARS-CoV-2 on Surfaces. mSphere 5(4):e00441-00420. https://doi.org/10.1128/mSphere.00441-20
143. Lee SE, Lee DY, Lee WG et al (2020) Detection of Novel Coronavirus on the Surface of Environmental Materials Contaminated by COVID-19 Patients in the Republic of Korea. Osong Public Health Res Perspect 11(3):128-132. https://doi.org/10.24171/j.phrp.2020.11.3.03
144. Razzini K, Castrica M, Menchetti L et al (2020) SARS-CoV-2 RNA detection in the air and on surfaces in the COVID-19 ward of a hospital in Milan, Italy. Sci Total Environ 742:140540. https://doi.org/10.1016/j.scitotenv.2020.140540
145. Marquès M, Domingo JL (2021) Contamination of inert surfaces by SARS-CoV-2: Persistence, stability and infectivity. A review. Environ Res 193:110559. https://doi.org/10.1016/j.envres.2020.110559

146. Kampf G, Brüggemann Y, Kaba HEJ et al (2020) Potential sources, modes of transmission and effectiveness of prevention measures against SARS-CoV-2. J Hosp Infect 106(4):678-697. https://doi.org/10.1016/j.jhin.2020.09.022
147. Deyab MA (2020) Coronaviruses widespread on nonliving surfaces: important questions and promising answers. Z Naturforsch C J Biosci 75(9-10):363-367. https://doi.org/10.1515/znc-2020-0105
148. Suman R, Javaid M, Haleem A, Vaishya R, Bahl S, Nandan D (2020) Sustainability of Coronavirus on Different Surfaces. J Clin Exp Hepatol 10(4):386-390. https://doi.org/10.1016/j.jceh.2020.04.020
149. Xie C, Zhao H, Li K et al (2020) The evidence of indirect transmission of SARS-CoV-2 reported in Guangzhou, China. BMC Public Health 20(1):1202. https://doi.org/10.1186/s12889-020-09296-y
150. Meyerowitz EA, Richterman A, Gandhi RT, Sax PE (2021) Transmission of SARS-CoV-2: A Review of Viral, Host, and Environmental Factors. Ann Intern Med 174(1):69-79. https://doi.org/10.7326/m20-5008
151. Colaneri M, Seminari E, Novati S et al (2020) Severe acute respiratory syndrome coronavirus 2 RNA contamination of inanimate surfaces and virus viability in a health care emergency unit. Clin Microbiol Infect 26(8):1094.e1-1094.e5. https://doi.org/10.1016/j.cmi.2020.05.009
152. Wilson AM, Weir MH, Bloomfield SF, Scott EA, Reynolds KA (2021) Modeling COVID-19 infection risks for a single hand-to-fomite scenario and potential risk reductions offered by surface disinfection. Am J Infect Control 49(6):846-848. https://doi.org/10.1016/j.ajic.2020.11.013
153. Harvey AP, Fuhrmeister ER, Cantrell M et al (2020) Longitudinal monitoring of SARS-CoV-2 RNA on high-touch surfaces in a community setting. medRxiv. https://doi.org/10.1101/2020.10.27.20220905
154. Pitol AK, Julian TR (2021) Community Transmission of SARS-CoV-2 by Surfaces: Risks and Risk Reduction Strategies. Environ Sci Technol Lett 8(3):263-269. https://doi.org/10.1021/acs.estlett.0c00966
155. Azuma K, Yanagi U, Kagi N, Kim H, Ogata M, Hayashi M (2020) Environmental factors involved in SARS-CoV-2 transmission: effect and role of indoor environmental quality in the strategy for COVID-19 infection control. Environ Health Prev Med 25(1):66. https://doi.org/10.1186/s12199-020-00904-2
156. Wang Y, Tian H, Zhang L et al (2020) Reduction of secondary transmission of SARS-CoV-2 in households by face mask use, disinfection and social distancing: a cohort study in Beijing, China. BMJ Glob Health 5(5). https://doi.org/10.1136/bmjgh-2020-002794
157. Santarpia JL, Rivera DN, Herrera VL et al (2020) Aerosol and surface contamination of SARS-CoV-2 observed in quarantine and isolation care. Sci Rep 10(1):12732. https://doi.org/10.1038/s41598-020-69286-3
158. Mahl MC, Sadler C (1975) Virus survival on inanimate surfaces. Can J Microbiol 21(6):819-823. https://doi.org/10.1139/m75-121
159. Gordon YJ, Gordon RY, Romanowski E, Araullo-Cruz TP (1993) Prolonged recovery of desiccated adenoviral serotypes 5, 8, and 19 from plastic and metal surfaces in vitro. Ophthalmology 100(12):1835-1839; discussion 1839-1840. https://doi.org/10.1016/s0161-6420(93)31389-8
160. Rabenau HF, Cinatl J, Morgenstern B, Bauer G, Preiser W, Doerr HW (2005) Stability and inactivation of SARS coronavirus. Med Microbiol Immunol 194(1-2):1-6. https://doi.org/10.1007/s00430-004-0219-0
161. Faix RG (1985) Survival of cytomegalovirus on environmental surfaces. J Pediatr 106(4):649-652. https://doi.org/10.1016/s0022-3476(85)80096-2
162. Piercy TJ, Smither SJ, Steward JA, Eastaugh L, Lever MS (2010) The survival of filoviruses in liquids, on solid substrates and in a dynamic aerosol. J Appl Microbiol 109(5):1531-1539. https://doi.org/10.1111/j.1365-2672.2010.04778.x

163. Westhoff Smith D, Hill-Batorski L, N'Jai A et al (2016) Ebola Virus Stability Under Hospital and Environmental Conditions. J Infect Dis 214(Suppl 3):S142-S144. https://doi.org/10.1093/infdis/jiw167
164. Sagripanti JL, Rom AM, Holland LE (2010) Persistence in darkness of virulent alphaviruses, Ebola virus, and Lassa virus deposited on solid surfaces. Arch Virol 155(12):2035-2039. https://doi.org/10.1007/s00705-010-0791-0
165. Fogarty R, Halpin K, Hyatt AD, Daszak P, Mungall BA (2008) Henipavirus susceptibility to environmental variables. Virus Res 132(1-2):140-144. https://doi.org/10.1016/j.virusres.2007.11.010
166. Wood JP, Choi YW, Wendling MQ, Rogers JV, Chappie DJ (2013) Environmental persistence of vaccinia virus on materials. Lett Appl Microbiol 57(5):399-404. https://doi.org/10.1111/lam.12126
167. Mahnel H (1987) Experimentelle Ergebnisse über die Stabilität von Pockenviren unter Labor- und Umweltbedingungen. J Vet Med B 34(6):449-464
168. Abad FX, Villena C, Guix S, Caballero S, Pinto RM, Bosch A (2001) Potential role of fomites in the vehicular transmission of human astroviruses. Appl Environ Microbiol 67(9):3904-3907. https://doi.org/10.1128/AEM.67.9.3904-3907.2001
169. Firquet S, Beaujard S, Lobert PE et al (2015) Survival of Enveloped and Non-Enveloped Viruses on Inanimate Surfaces. Microbes Environ 30(2):140-144. https://doi.org/10.1264/jsme2.ME14145
170. Mocé-Llivina L, Papageorgiou GT, Jofre J (2006) A membrane-based quantitative carrier test to assess the virucidal activity of disinfectants and persistence of viruses on porous fomites. J Virol Methods 135(1):49-55. https://doi.org/10.1016/j.jviromet.2006.01.021
171. D'Souza DH, Sair A, Williams K et al (2006) Persistence of caliciviruses on environmental surfaces and their transfer to food. Int J Food Microbiol 108(1):84-91. https://doi.org/10.1016/j.ijfoodmicro.2005.10.024
172. Clay S, Maherchandani S, Malik YS, Goyal SM (2006) Survival on uncommon fomites of feline calicivirus, a surrogate of noroviruses. Am J Infect Control 34(1):41-43. https://doi.org/10.1016/j.ajic.2005.05.013
173. Buckley D, Fraser A, Huang G, Jiang X (2017) Recovery optimization and survival of the human norovirus surrogates feline calicivirus and murine norovirus on carpet. Appl Environ Microbiol 83(22). https://doi.org/10.1128/aem.01336-17
174. Kim SJ, Si J, Lee JE, Ko G (2012) Temperature and humidity influences on inactivation kinetics of enteric viruses on surfaces. Environ Sci Technol 46(24):13303-13310. https://doi.org/10.1021/es3032105
175. Trudel-Ferland M, Jubinville E, Jean J (2021) Persistence of Hepatitis A Virus RNA in water, on non-porous surfaces, and on blueberries. Front Microbiol 12:618352. https://doi.org/10.3389/fmicb.2021.618352
176. Warnes SL, Keevil CW (2013) Inactivation of norovirus on dry copper alloy surfaces. PLoS One 8(9):e75017. https://doi.org/10.1371/journal.pone.0075017
177. Keswick BH, Pickering LK, DuPont HL, Woodward WE (1983) Survival and detection of rotaviruses on environmental surfaces in day care centers. Appl Environ Microbiol 46(4):813-816. https://doi.org/doi:10.1128/aem.46.4.813-816.1983
178. Tamrakar S, Henley J, Gurian P et al (2017) Persistence analysis of poliovirus on three different types of fomites. J Appl Microbiol 122(2):522-530. https://doi.org/10.1111/jam.13299
179. Sattar SA, Lloyd-Evans N, Springthorpe VS, Nair RC (1986) Institutional outbreaks of rotavirus diarrhoea: potential role of fomites and environmental surfaces as vehicles for virus transmission. J Hyg (Lond) 96(2):277-289. https://doi.org/10.1017/s0022172400066055
180. Arthur SE, Gibson KE (2016) Environmental persistence of Tulane virus - a surrogate for human norovirus. Can J Microbiol 62(5):449-454. https://doi.org/10.1139/cjm-2015-0756

181. Sizun J, Yu MWN, Talbot PJ (2000) Survival of human coronaviruses 229E and OC43 in suspension and after drying onsurfaces: a possible source ofhospital-acquired infections. J Hosp Infect 46(1):55-60. https://doi.org/https://doi.org/10.1053/jhin.2000.0795
182. Warnes SL, Little ZR, Keevil CW (2015) Human coronavirus 229E remains infectious on common touch surface materials. mBio 6(6):e01697-15. https://doi.org/10.1128/mBio.01697-15
183. Perry KA, Coulliette AD, Rose LJ, Shams AM, Edwards JR, Noble-Wang JA (2016) Persistence of influenza A (H1N1) virus on stainless steel surfaces. Appl Environ Microbiol 82(11):3239-3245. https://doi.org/10.1128/Aem.04046-15
184. Thompson KA, Bennett AM (2017) Persistence of influenza on surfaces. J Hosp Infect 95(2):194-199. https://doi.org/10.1016/j.jhin.2016.12.003
185. Bean B, Moore BM, Sterner B, Peterson LR, Gerding DN, Balfour HH, Jr. (1982) Survival of influenza viruses on environmental surfaces. J Infect Dis 146(1):47-51. https://doi.org/10.1093/infdis/146.1.47
186. Greatorex JS, Digard P, Curran MD et al (2011) Survival of influenza A(H1N1) on materials found in households: implications for infection control. PLoS One 6(11):e27932. https://doi.org/10.1371/journal.pone.0027932
187. van Doremalen N, Bushmaker T, Munster VJ (2013) Stability of Middle East respiratory syndrome coronavirus (MERS-CoV) under different environmental conditions. Euro Surveill 18(38):pii=20590. https://doi.org/10.2807/1560-7917.es2013.18.38.20590
188. Brady MT, Evans J, Cuartas J (1990) Survival and disinfection of parainfluenza viruses on environmental surfaces. Am J Infect Control 18(1):18-23. https://doi.org/10.1016/0196-6553(90)90206-8
189. Hall CB, Douglas RJ, Geiman JM (1980) Possible transmission by fomites of respiratory syncytial virus. J Infect Dis 141(1):98-102. https://doi.org/10.1093/infdis/141.1.98
190. Sattar SA, Karim YG, Springthorpe VS, Johnson-Lussenburg CM (1987) Survival of human rhinovirus type 14 dried onto nonporous inanimate surfaces: effect of relative humidity and suspending medium. Can J Microbiol 33(9):802-806. https://doi.org/10.1139/m87-136
191. Reed SE (1975) An investigation of the possible transmission of Rhinovirus colds through indirect contact. J Hyg (Lond) 75(2):249-258. https://doi.org/10.1017/s0022172400047288
192. Duan SM, Zhao XS, Wen RF et al (2003) Stability of SARS coronavirus in human specimens and environment and its sensitivity to heating and UV irradiation. Biomed Environ Sci 16(3):246-255
193. Chan KH, Peiris JS, Lam SY, Poon LL, Yuen KY, Seto WH (2011) The Effects of Temperature and Relative Humidity on the Viability of the SARS Coronavirus. Adv Virol 2011:734690. https://doi.org/10.1155/2011/734690
194. Lai MY, Cheng PK, Lim WW (2005) Survival of severe acute respiratory syndrome coronavirus. Clin Infect Dis 41(7):e67-71. https://doi.org/10.1086/433186
195. Raiteux J, Eschlimann M, Marangon A et al (2021) Inactivation of SARS-CoV-2 by Simulated Sunlight on Contaminated Surfaces. Microbiol Spectr 9(1):e0033321. https://doi.org/10.1128/Spectrum.00333-21
196. Bardell D (1990) Survival of herpes simplex virus type 1 on some frequently touched objects in the home and public buildings. Microbios 63(256-257):145-150
197. Bardell D (1993) Survival of herpes simplex virus type 1 in saliva and tap water contaminating some common objects. Microbios 74(299):81-87
198. Nerurkar LS, West F, May M, Madden DL, Sever JL (1983) Survival of herpes simplex virus in water specimens collected from hot tubs in spa facilities and on plastic surfaces. JAMA 250(22):3081-3083. https://doi.org/10.1001/jama.1983.03340220049032
199. Barre-Sinoussi F, Nugeyre MT, Chermann JC (1985) Resistance of AIDS virus at room temperature. Lancet 2(8457):721-722. https://doi.org/10.1016/s0140-6736(85)92955-1
200. Roden RB, Lowy DR, Schiller JT (1997) Papillomavirus is resistant to desiccation. J Infect Dis 176(4):1076-1079. https://doi.org/10.1086/516515

201. The Center Food Security Public Health (CFSPH) (2017) Eastern, Western and Venezuelan Equine Encephalomyelitis. Minor Updates: May 2017. https://www.cfsph.iastate.edu/Factsheets/pdfs/easter_wester_venezuelan_equine_encephalomyelitis.pdf. Zugegriffen: 07. Juli 2022
202. Guzman H, Ding X, Xiao SY, Tesh RB (2005) Duration of infectivity and RNA of Venezuelan equine encephalitis, West Nile, and yellow fever viruses dried on filter paper and maintained at room temperature. Am J Trop Med Hyg 72(4):474-477. https://doi.org/10.4269/ajtmh.2005.72.474
203. Bond WW, Favero MS, Petersen NJ, Gravelle CR, Ebert JW, Maynard JE (1981) Survival of hepatitis B virus after drying and storage for one week. Lancet 1(8219):550-551. https://doi.org/10.1016/s0140-6736(81)92877-4
204. Favero MS, Bond WW, Petersen NJ, Berquist KR, Maynard JE (1974) Detection methods for study of the stability of hepatitis B antigen on surfaces. J Infect Dis 129(2):210-212
205. Paintsil E, Binka M, Patel A, Lindenbach BD, Heimer R (2014) Hepatitis C virus maintains infectivity for weeks after drying on inanimate surfaces at room temperature: implications for risks of transmission. J Infect Dis 209(8):1205-1211. https://doi.org/10.1093/infdis/jit648
206. Doerrbecker J, Friesland M, Ciesek S et al (2011) Inactivation and survival of hepatitis C virus on inanimate surfaces. J Infect Dis 204(12):1830-1838. https://doi.org/10.1093/infdis/jir535
207. U.S. Environmental Protection Agency (EPA) (2014) EPA/600/R-14/074, Persistence of Categories A and B Select Agents in Environmental Matrices. EPA, Washington, DC https://cfpub.epa.gov/si/si_public_file_download.cfm?p_download_id=520009&Lab=NHSRC . Zugegriffen: 07. Juli 2022
208. Ward RL, Bernstein DI, Young EC, Sherwood JR, Knowlton DR, Schiff GM (1986) Human rotavirus studies in volunteers: determination of infectious dose and serological response to infection. J Infect Dis 154(5):871-880. https://doi.org/10.1093/infdis/154.5.871
209. Paton JC, Paton AW (1998) Pathogenesis and diagnosis of Shiga toxin-producing Escherichia coli infections. Clin Microbiol Rev 11(3):450-479. https://doi.org/10.1128/cmr.11.3.450
210. Pang XL, Joensuu J, Vesikari T (1999) Human calicivirus-associated sporadic gastroenteritis in Finnish children less than two years of age followed prospectively during a rotavirus vaccine trial. Pediatr Infect Dis J 18(5):420-426. https://doi.org/10.1097/00006454-199905000-00005
211. Lawley TD, Clare S, Deakin LJ et al (2010) Use of purified Clostridium difficile spores to facilitate evaluation of health care disinfection regimens. Appl Environ Microbiol 76(20):6895-6900. https://doi.org/10.1128/aem.00718-10
212. Porter CK, Riddle MS, Tribble DR et al (2011) A systematic review of experimental infections with enterotoxigenic Escherichia coli (ETEC). Vaccine 29(35):5869-5885. https://doi.org/10.1016/j.vaccine.2011.05.021
213. Dancer SJ (2014) Controlling hospital-acquired infection: focus on the role of the environment and new technologies for decontamination. Clin Microbiol Rev 27(4):665-690. https://doi.org/10.1128/CMR.00020-14
214. Kothary MH, Babu US (2001) Infective dose of foodborne pathogens in volunteers: A review. J Food Saf 21(1):49-68. https://doi.org/https://doi.org/10.1111/j.1745-4565.2001.tb00307.x
215. Blaser MJ, Newman LS (1982) A review of human salmonellosis: I. Infective dose. Rev Infect Dis 4(6):1096-1106. https://doi.org/10.1093/clinids/4.6.1096
216. Schmid-Hempel P, Frank SA (2007) Pathogenesis, virulence, and infective dose. PLoS Pathog 3(10):1372-1373. https://doi.org/10.1371/journal.ppat.0030147
217. Kwiecinska-Pirog J, Bogiel T, Skowron K, Wieckowska E, Gospodarek E (2014) Proteus mirabilis biofilm - qualitative and quantitative colorimetric methods-based evaluation. Braz J Microbiol 45(4):1423-1431. https://doi.org/10.1590/s1517-83822014000400037
218. Otter JA, Vickery K, Walker JT et al (2015) Surface-attached cells, biofilms and biocide susceptibility: implications for hospital cleaning and disinfection. J Hosp Infect 89(1):16-27. https://doi.org/10.1016/j.jhin.2014.09.008

219. Costa DM, Johani K, Melo DS et al (2019) Biofilm contamination of high-touched surfaces in intensive care units: epidemiology and potential impacts. Lett Appl Microbiol 68(4):269-276. https://doi.org/10.1111/lam.13127
220. Gause GF (2019) The Struggle for Existence: A Classic of Mathematical Biology and Ecology. Dover Publications, New York
221. Nocker A, Burr M, Camper A (2014) Pathogens in Water and Biofilms. In: Percival SL, Yates MV, Williams DW et al (Hrsg) Microbiology of Waterborne Disease, 2. Aufl. Elsevier, Amsterdam, S 3-32
222. Umweltbundesamt (UBA) (2021) Richtwerte für 2-Propanol in der Innenraumluft. Bundesgesundheitsbl 64(10):1318-1327. https://doi.org/10.1007/s00103-021-03402-0
223. World Health Organization (WHO) (1990) 2-Propanol Environmental Health Criteria 103. https://apps.who.int/iris/bitstream/handle/10665/39144/9241571039-eng.pdf?sequence=1. Zugegriffen: 07. Juli 2022
224. Yoshio Y, Nagata E (2003) Measurement of Odor Threshold by Triangular Odor Bag Method. In: Office of Odor, Noise and Vibration Environmental Management Bureau, Ministry of the Environment (Hrsg) Olfactory Measurement Method in Japan. S 125-134 https://www.env.go.jp/content/900450159.pdf. Zugegriffen: 07. Jul. 2022
225. Kramer A, Assadian O (Hrsg)(2008) Wallhäußers Praxis der Sterilisation, Antiseptik und Konservierung. Thieme, Stuttgart
226. Verordnung (EG) Nr. 1272/2008 des Europäischen Parlaments und des Rates vom 16. Dezember 2008 über die Einstufung, Kennzeichnung und Verpackung von Stoffen und Gemischen, zur Änderung und Aufhebung der Richtlinien 67/548/EWG und 1999/45/EG und zur Änderung der Verordnung (EG) Nr. 1907/2006. Amtsblatt der Europäischen Union 51 (L353):1-1355
227. Carling PC, Parry MF, Von Beheren SM, Healthcare Environmental Hygiene Study G (2008) Identifying opportunities to enhance environmental cleaning in 23 acute care hospitals. Infect Control Hosp Epidemiol 29(1):1-7. https://doi.org/10.1086/524329
228. Jatzwauk L, Emmrich M, Gerth K et al (2018) MIQ 23 Krankenhaushygienische Untersuchungen. Elsevier, München
229. Al-Hamad A, Maxwell S (2008) How clean is clean? Proposed methods for hospital cleaning assessment. J Hosp Infect 70(4):328-334. https://doi.org/10.1016/j.jhin.2008.08.006
230. Dancer SJ (2011) Hospital cleaning in the 21st century. Eur J Clin Microbiol Infect Dis 30(12):1473-1481. https://doi.org/10.1007/s10096-011-1250-x
231. Deshpande A, Donskey CJ (2017) Practical Approaches for Assessment of Daily and Post-discharge Room Disinfection in Healthcare Facilities. Curr Infect Dis Rep 19(9):32. https://doi.org/10.1007/s11908-017-0585-6
232. Mitchell BG, Wilson F, Dancer SJ, McGregor A (2013) Methods to evaluate environmental cleanliness in healthcare facilities. Healthcare infection 18(1):23-30. https://doi.org/https://doi.org/10.1071/HI12047
233. Amodio E, Dino C (2014) Use of ATP bioluminescence for assessing the cleanliness of hospital surfaces: a review of the published literature (1990-2012). J Infect Public Health 7(2):92-98. https://doi.org/10.1016/j.jiph.2013.09.005
234. Nante N, Ceriale E, Messina G, Lenzi D, Manzi P (2017) Effectiveness of ATP bioluminescence to assess hospital cleaning: a review. J Prev Med Hyg 58(2):E177-E183
235. Sherlock O, O'Connell N, Creamer E, Humphreys H (2009) Is it really clean? An evaluation of the efficacy of four methods for determining hospital cleanliness. J Hosp Infect 72(2):140-146. https://doi.org/10.1016/j.jhin.2009.02.013
236. Willis C, Morley R, Westbury J, Greenwood M, Pallett A (2007) Evaluation of ATP bioluminescence swabbing as a monitoring and training tool for effective hospital cleaning. BJIC 8(5):17-21. https://doi.org/10.1177/1469044607083604
237. Casini B, Tuvo B, Totaro M, Aquino F, Baggiani A, Privitera G (2018) Evaluation of the Cleaning Procedure Efficacy in Prevention of Nosocomial Infections in Healthcare Facilities Using

Cultural Method Associated with High Sensitivity Luminometer for ATP Detection. Pathogens 7(3):E71. https://doi.org/10.3390/pathogens7030071
238. Omidbakhsh N, Ahmadpour F, Kenny N (2014) How Reliable Are ATP Bioluminescence Meters in Assessing Decontamination of Environmental Surfaces in Healthcare Settings? PLoS One 9(6):e99951. https://doi.org/10.1371/journal.pone.0099951
239. Brown E, Eder AR, Thompson KM (2010) Do surface and cleaning chemistries interfere with ATP measurement systems for monitoring patient room hygiene? J Hosp Infect 74(2):193-195. https://doi.org/10.1016/j.jhin.2009.10.006
240. Shimoda T, Yano R, Nakamura S et al (2015) ATP bioluminescence values are significantly different depending upon material surface properties of the sampling location in hospitals. BMC Res Notes 8:807. https://doi.org/10.1186/s13104-015-1757-9
241. Shama G, Malik DJ (2013) The uses and abuses of rapid bioluminescence-based ATP assays. Int J Hyg Environ Health 216(2):115-125. https://doi.org/10.1016/j.ijheh.2012.03.009
242. Carling PC, Briggs JL, Perkins J, Highlander D (2006) Improved cleaning of patient rooms using a new targeting method. Clin Infect Dis 42(3):385-388. https://doi.org/10.1086/499361
243. Hausemann A, Grunewald M, Otto U, Heudorf U (2018) Cleaning and disinfection of surfaces in hospitals. Improvement in quality of structure, process and outcome in the hospitals in Frankfurt/Main, Germany, in 2016 compared to 2014. GMS Hyg Infect Control 13:Doc06. https://doi.org/10.3205/dgkh000312
244. Boyce JM, Havill NL, Havill HL, Mangione E, Dumigan DG, Moore BA (2011) Comparison of fluorescent marker systems with 2 quantitative methods of assessing terminal cleaning practices. Infect Control Hosp Epidemiol 32(12):1187-1193. https://doi.org/10.1086/662626
245. Alfa MJ, Dueck C, Olson N et al (2008) UV-visible marker confirms that environmental persistence of Clostridium difficile spores in toilets of patients with C. difficile-associated diarrhea is associated with lack of compliance with cleaning protocol.e. BMC Infect Dis 8:64. https://doi.org/10.1186/1471-2334-8-64
246. Snyder GM, Holyoak AD, Leary KE, Sullivan BF, Davis RB, Wright SB (2013) Effectiveness of visual inspection compared with non-microbiologic methods to determine the thoroughness of post-discharge cleaning. Antimicrob Resist Infect Control 2:26. https://doi.org/10.1186/2047-2994-2-26
247. Carling PC, Parry MM, Rupp ME et al (2008) Improving cleaning of the environment surrounding patients in 36 acute care hospitals. Infect Control Hosp Epidemiol 29(11):1035-1041. https://doi.org/10.1086/591940
248. Goodman ER, Platt R, Bass R, Onderdonk AB, Yokoe DS, Huang SS (2008) Impact of an environmental cleaning intervention on the presence of methicillin-resistant Staphylococcus aureus and vancomycin-resistant enterococci on surfaces in intensive care unit rooms. Infect Control Hosp Epidemiol 29(7):593-599. https://doi.org/10.1086/588566
249. Blue J, O'Neill C, Speziale P, Revill J, Ramage L, Ballantyne L (2008) Use of a fluorescent chemical as a quality indicator for a hospital cleaning program. Can J Infect Control 23(4):216-219
250. Munoz-Price LS, Birnbach DJ, Lubarsky DA et al (2012) Decreasing Operating Room Environmental Pathogen Contamination through Improved Cleaning Practice. Infect Control Hosp Epidemiol 33(9):897-904. https://doi.org/10.1086/667381
251. Sitzlar B, Deshpande A, Fertelli D, Kundrapu S, Sethi AK, Donskey CJ (2013) An Environmental Disinfection Odyssey: Evaluation of Sequential Interventions to Improve Disinfection of Clostridium difficile Isolation Rooms. Infect Control Hosp Epidemiol 34(5):459-465. https://doi.org/10.1086/670217
252. Trajtman AN, Manickam K, Macrae M, Bruning NS, Alfa MJ (2013) Continuing performance feedback and use of the ultraviolet visible marker to assess cleaning compliance in the healthcare environment. J Hosp Infect 84(2):166-172. https://doi.org/10.1016/j.jhin.2013.03.004

253. Woltering R, Hoffmann G, Isermann J, Heudorf U (2016) Flächenreinigung und Desinfektion im Krankenhaus. Verbesserung durch objektive Überwachung und Intervention. Gesundheitswesen 78(11):759-764. https://doi.org/10.1055/s-0035-1545267
254. Hung IC, Chang HY, Cheng A et al (2018) Application of a fluorescent marker with quantitative bioburden methods to assess cleanliness. Infect Control Hosp Epidemiol 39(11):1296-1300. https://doi.org/10.1017/ice.2018.222
255. Smith A, Taggart LR, Lebovic G, Zeynalova N, Khan A, Muller MP (2016) Clostridium difficile infection incidence: impact of audit and feedback programme to improve room cleaning. J Hosp Infect 92(2):161-166. https://doi.org/10.1016/j.jhin.2015.11.001
256. Heudorf U, Gasteyer S, Samoiski Y, Voigt K (2012) Flächenreinigung und -desinfektion in Altenpflegeheimen. Bundesgesundheitsbl 55(8):961-969. https://doi.org/10.1007/s00103-012-1513-4
257. Boyce J (2014) The inanimate environment. In: Jarvis WR (Hrsg) Bennett and Brachman's Hospital Infection, 6. Aufl. Williams und Wilkins, Philadelphia, S 277–292
258. Griffith CJ, Cooper RA, Gilmore J, Davies C, Lewis M (2000) An evaluation of hospital cleaning regimes and standards. J Hosp Infect 45(1):19-28. https://doi.org/10.1053/jhin.1999.0717
259. Malik RE, Cooper RA, Griffith CJ (2003) Use of audit tools to evaluate the efficacy of cleaning systems in hospitals. Am J Infect Control 31(3):181-187. https://doi.org/10.1067/mic.2003.34
260. Mulvey D, Redding P, Robertson C et al (2011) Finding a benchmark for monitoring hospital cleanliness. J Hosp Infect 77(1):25-30. https://doi.org/10.1016/j.jhin.2010.08.006
261. Huang YS, Chen YC, Chen ML et al (2015) Comparing visual inspection, aerobic colony counts, and adenosine triphosphate bioluminescence assay for evaluating surface cleanliness at a medical center. Am J Infect Control 43(8):882-886. https://doi.org/10.1016/j.ajic.2015.03.027
262. Knape L, Hambraeus A, Lytsy B (2015) The adenosine triphosphate method as a quality control tool to assess 'cleanliness' of frequently touched hospital surfaces. J Hosp Infect 91(2):166-170. https://doi.org/10.1016/j.jhin.2015.06.011
263. Verbund für Angewandte Hygiene e.V. (VAH), Desinfektionsmittel-Kommission (2021) Anforderungen und Methoden zur VAH-Zertifizierung chemischer Desinfektionsverfahren. Kapitel 1 bis 4: Stand 1. November 2021. https://vah-online.de/files/download/ebooks/211109-VAH-Methodenbuch-Kapitel-1-4-Gesamt.pdf. Zugegriffen: 07. Juli 2022
264. Verbund für Angewandte Hygiene e.V. (VAH), Desinfektionsmittel-Kommission (2019) Anforderungen und Methoden zur VAH-Zertifizierung chemischer Desinfektionsverfahren. https://vah-online.de/files/download/ebooks/eBook_VAH_Methoden_Anforderungen.pdf. Zugegriffen: 07. Juli 2022
265. Verbund für Angewandte Hygiene (VAH), Desinfektionsmittel-Kommission (2021) Qualitätssicherung der Aussagen zur Viruswirksamkeit von Desinfektionsmitteln: VAH präzisiert Anforderungen an die Viruswirksamkeit in der VAH-Liste. Hyg Med 46(11):243-244
266. Verbund für Angewandte Hygiene e.V. (VAH), Desinfektionsmittel-Kommission (2021) Anforderungen und Methoden zur VAH-Zertifizierung chemischer Desinfektionsverfahren. Anhang V (Anforderungen an die Viruswirksamkeit). https://vah-online.de/files/download/VAH_Methodenbuch_Anhang_Viruswirksamkeit_1Nov2021.pdf. Zugegriffen: 07. Juli 2022
